# Understanding barriers to effective malaria diagnosis and treatment: A mixed-methods study in Uganda, Nigeria, and Ivory Coast

**DOI:** 10.1371/journal.pgph.0006921

**Published:** 2026-08-17

**Authors:** Nekoye Otsyula, Nareen Krishna Polavarapu, Annie Syntosi, Will Bench, Gursimran Kaur, Anna Meadows, Rebecca L. West, Matthew Dewhurst, Pushpendra Kumar Mishra, Irene Olisamedua Obi, Olamide Idayat Adigun, Emmanuel Nespaul Odongo, Peninnah Nanziri, Caroline Boulton

**Affiliations:** 1 Novartis Pharma Services Limited, Nairobi, Kenya; 2 Novartis Health Care Private Limited, Chennai, India; 3 Novartis Pharma AG, Basel, Switzerland; 4 Ipsos, London, United Kingdom; 5 Ipsos, Lagos, Nigeria; 6 Ipsos, Kampala, Uganda; Fundacao Oswaldo Cruz, BRAZIL

## Abstract

Malaria remains a global health challenge despite decades of intervention. We conducted a mixed-methods study using the MAPPS behavioural science framework to identify and explore behaviours relating to timely access and better adherence to antimalarials, and to offer initial ideas for interventions. Research was conducted in Uganda, Nigeria, and Ivory Coast following local ethical approval. First, qualitative face-to-face interviews were conducted with n = 18 adults with experience of malaria, n = 36 caregivers of children with experience of malaria, and n = 36 healthcare professionals (HCPs) with experience treating patients with malaria, to identify and explore the behavioural drivers and barriers to access and adherence. Cross-sectional quantitative surveys were then conducted face-to-face with respondents with experience of malaria (n = 311 adults, n = 458 caregivers, n = 532 HCPs) to quantify the incidence of the different drivers and barriers. The sample included representation from rural and urban areas, and public and private healthcare settings. This research identified key behaviours that delayed access and adherence. One-third (33%) of adults and caregivers reported delayed care-seeking from a healthcare facility when experiencing symptoms. Over a third (36%) reported not being offered a diagnostic test to confirm their diagnosis when they last sought care for malaria. 56% reported prematurely discontinuing their antimalarial medication, with 41% stating that treatment can be stopped once symptoms subside. Despite current interventions, behavioural barriers to effective malaria treatment and prevention persist. This research identified and quantified key barriers, including difficulty in identifying symptoms specific to malaria, lack of specified responsibility in testing and difficulties adhering to treatment. Communication with, and education of, patients and caregivers that emphasise the importance of seeking early care are recommended. In addition, interventions encouraging HCPs to offer, and patients or caregivers to request, appropriate testing should be explored. Finally, the use of social norming interventions to promote completion of treatment courses is also recommended.

## Introduction

Malaria remains a major cause of morbidity and mortality in sub-Saharan Africa and represents one of the most critical public health challenges across the continent [1]. In 2025 the World Health Organisation (WHO) reported 280 million malaria cases and more than 600,000 deaths worldwide; of these, the WHO African Region accounts for 94% [2]. Uganda, Nigeria, and Ivory Coast are among the 11 “High Burden to High Impact” [3] countries which account for around 70% of the global malaria burden [2]. Nigeria individually contributes to approximately 27% of global malaria cases and 23% of deaths, making it the highest-burden country globally [2,4]. In Uganda and Ivory Coast, malaria is suspected to account for between 30–50% and 40% of outpatient visits respectively [1,5]. In addition to high levels of morbidity and mortality, the healthcare costs associated with malaria treatment also hinder economic development in these countries [6–10].

Current malaria control strategies include vector control measures, vaccination and the use of antimalarial drugs in prevention and treatment. Vector control aims to reduce malaria transmission by targeting *Anopheles* mosquitoes that transmit *Plasmodium* parasites, including mass distribution of insecticide-treated nets and indoor residual spraying. In parallel, there has been a large historical focus on improving access to malaria testing and treatment. Artemisinin combination therapies (ACTs) are the primary treatment for malaria and are routinely used in the management of uncomplicated malaria [11]. However, despite these efforts, the burden of malaria persists and partial resistance to artemisinin has emerged in several malaria-endemic regions including Eastern Africa [12–14] and is suspected in Central Africa [15]. The ongoing effectiveness of these medical interventions relies heavily on human behaviour [16]. Prompt access to antimalarial treatment is essential to effectively manage the disease, prevent severe complications, and reduce the potential for onward transmission and drug resistance. Furthermore, the development and spread of partial artemisinin resistance is driven by factors that include adherence challenges; non-adherence to ACTs can directly reduce treatment efficacy and contribute to the selection of artemisinin-resistant strains [12].

Understanding the specific behavioural barriers that influence delayed care-seeking, inconsistent diagnostic testing, and non-adherence to ACTs is critical to optimising current malaria control strategies and preventing the spread of artemisinin resistance. The rationale for this study was to address this gap by systematically diagnosing the behavioural influences that impede effective malaria treatment through delaying access and adherence to treatment. This study aimed to identify and explore behaviours relating to timely access, diagnostic testing, and adherence to antimalarials among adults, caregivers, and healthcare professionals in Uganda, Nigeria, and Côte d’Ivoire, and to offer targeted intervention guidance to improve both access and adherence.

## Methodology

### Ethical statement

Local ethical approvals were obtained in all three study countries: Sokoto and Taraba State Ministry of Health, Health Research Ethics Committee in Nigeria (References - SMH/1580/V.IV and TRSHREC/2023/024), National Health and Life Sciences Ethics Committee (CNESVS) in Ivory Coast (Reference - 244–23/MSHPCMU/CNESVS-km) and The AIDS Support Organisation Research Ethics Committee (TASO) in Uganda (Reference - TASO-2023–322). Informed consent was obtained from all participants prior to their involvement in either the qualitative or quantitative phase. All data were handled in accordance with GDPR and local data protection laws, ensuring participant confidentiality and data security.

### Study design

**Fig 1:**
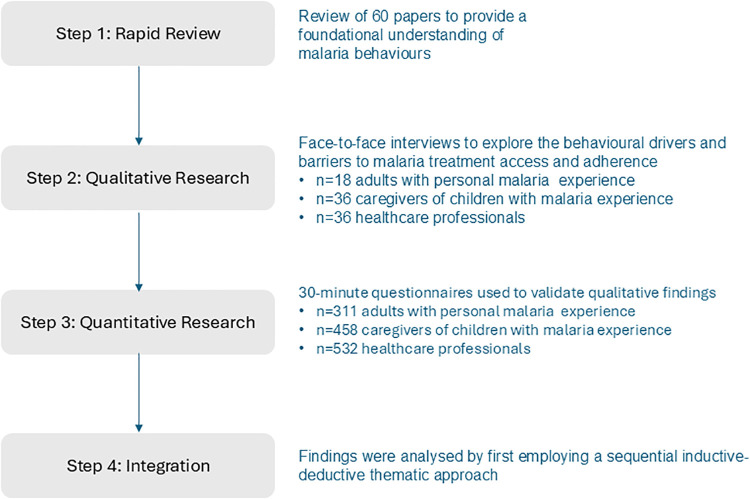
Methodology Overview.

The study was a mixed-methods sequential exploratory study [17] informed by an initial rapid review [18] (Fig 1). The rapid review provided the framing for the research and helped to define key decision points along the malaria patient pathway. The behaviours related to these decision points were then explored during the qualitative data collection and analysed using the MAPPS framework to diagnose their influences. Findings from this phase were used to develop and refine the quantitative survey enabling validation and sizing of the qualitative insights.

### Study conceptual framework: MAPPS

This research was conducted to understand the barriers that prevent appropriate access and adherence to antimalarial treatments to help identify appropriate solutions. As is the case with most behaviour, the influences on access and adherence to antimalarials are multiple and complex. To enable generation of appropriate intervention ideas, a comprehensive and ordered examination of the current situation was necessary: What is currently happening, what relevant behaviours are evident and what needs to change?

**Fig 2:**
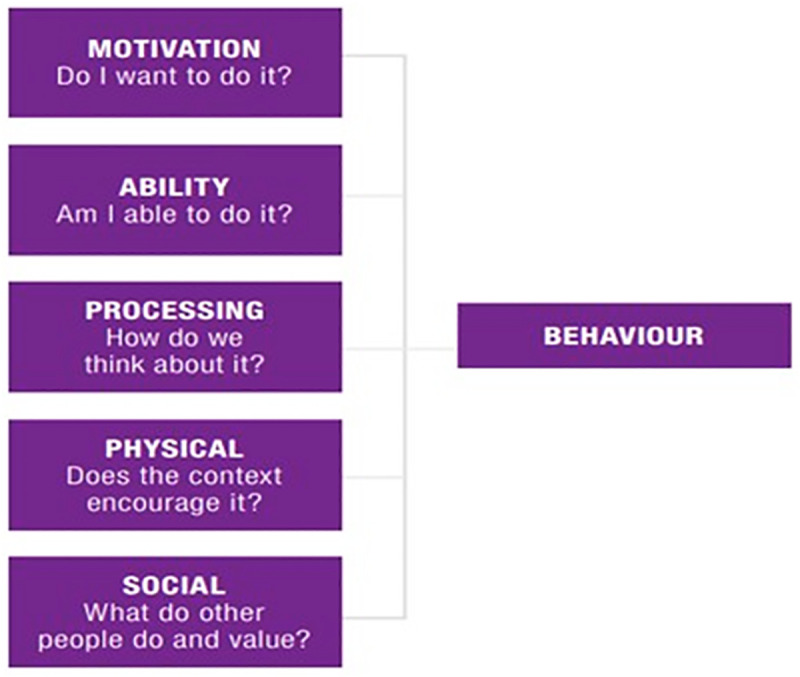
The MAPPS behaviour change framework [19].

To fully explore and understand current behaviour Ipsos’ MAPPS model of behaviour change was applied as a conceptual framework [19]. MAPPS (Fig 2) is based on the premise that behaviour is the product of five distinct dimensions: Motivation, Ability, Processing, Physical and Social. The dimensions of the framework facilitated the diagnosis of the influences involved in producing current behaviours or preventing desired behaviours that relate to effective malaria treatment. This enabled the development of initial intervention ideas tailored to the specific context of malaria treatment in Uganda, Nigeria and Ivory Coast.

### Rapid review

First, a rapid review of the literature available on the subject was conducted to provide a foundational understanding of behaviours related to the malaria treatment journey and identify current intervention strategies in the countries of interest. The publications reviewed were limited to the timeframe 2010/01/01–2023/12/31 to ensure literature was relevant to the current and future malaria landscape. The rapid review consisted of reviewing c.60 papers on PubMed found using one or more of the following search terms, “Malaria”, “Nigeria OR Uganda OR Ivory Coast AND behavioural research”, “health behaviour”, “antimalarial adherence”, “malaria interventions”, “malaria educational programmes”, “patient acceptance of malaria care”. We excluded any papers that focussed only on vector control methods as this research sought to focus on barriers to access and adherence to treatment.

Based on the rapid review, the research team identified three key decision points across the caregiver and adult patient pathway relating to access and adherence behaviours. These decision points were: ‘Shall I seek care?’, ‘Where shall I seek care?’ and ‘Shall I/ child take the full dose of treatment?’. These decision points were used to inform the qualitative and quantitative research materials, which sought to further explore the behaviour resulting from each decision and the influences on those behaviours. Similar decision points were identified amongst healthcare professionals: ‘Shall I test?’, ‘How should I treat this patient?’ and ‘How should I engage with this patient during treatment?’

### Study setting

Two districts in each country were chosen to ensure a comprehensive understanding of different locations within each setting and gain broader perspectives on malaria treatment access and adherence. The selection of these districts was based on two primary factors, representation of both rural and urban populations, and the practical feasibility of conducting research or activities within those areas. The following districts were included: Sokoto and Taraba in Nigeria, Acholi and East-Central Busoga in Uganda, and La Vallee Du Bandaman and Bas-Sassandra in Ivory Coast. Data collection took place in both rural and urban areas, encompassing both public and private healthcare settings.

### Qualitative methodology

Purposive sampling was employed both for adults aged 18 or above and healthcare professionals and was enforced through strict inclusion and exclusion criteria applied via a screening questionnaire. For adults, inclusion criteria required participants to be 18 years or above who either had suffered from malaria themselves in the past five years or were the parents/caregivers of children who had experienced malaria in the past five years. This ensured that participants were able to provide insight into their behaviour in relation to each of the decision points identified in the rapid review. Following findings from the rapid review, we also engaged community leaders (e.g., political, local, religious leaders) in the countries of interest due to their wide influence on health behaviour in the community [4,20,21]. A mix of genders and those in rural and urban settings were also recruited.

For healthcare professionals, inclusion criteria required them to be actively practising nurses, community health workers, or pharmacists with direct experience treating malaria patients and to have been in their current role for at least 2 years. Individuals who did not meet these specific criteria were excluded. Quotas were also set to ensure a diverse mix of respondents across locations and facility types (S3 Table).

Prior to fieldwork, all local recruiters and researchers underwent rigorous training led by researchers at Ipsos. This training involved comprehensive walkthroughs of the screening questionnaire and extensive role-playing exercises simulating various respondent scenarios to ensure accurate participant screening and adherence to the inclusion/exclusion criteria.

All qualitative fieldwork was conducted between 29^th^ January 2024–10^th^ April 2024. Recruitment for adult participants began at health service delivery points where malaria treatment is provided, without the use of formal sampling frames or recruitment lists. HCPs were recruited face-to-face through community health facilities that provided malaria treatment services. Recruiters approached individuals within these health facilities to take part in a screening questionnaire to check eligibility for participation. Participants across all three countries were recontacted after initial recruitment to confirm their participation and scheduling of interviews. Participants received a consent form outlining the study’s purpose, estimated duration, participant responsibilities, and any associated risks or benefits. Informed consent was obtained from all participants prior to data collection. There was only one reported refusal from an adult respondent in Nigeria.

Following this, face-to-face semi-structured interviews were conducted by trained local researchers, allowing for in-depth exploration of behaviours related to malaria treatment access and adherence.

Thematic analysis of the qualitative data was guided by the MAPPS [19] framework. Questions and responses from each interview were organised in Excel to collate the qualitative data. Excel was purposefully selected as the primary analytical tool over dedicated qualitative software to facilitate simultaneous, collaborative coding across our team. A standardised Excel coding matrix was utilised to facilitate collaborative analysis across our research teams. To ensure strict data integrity across multiple locations, the raw interview data was locked to prevent any unauthorised alterations. Researchers inputted their thematic codes into a separate, shared analytical framework, which allowed for secure, concurrent coding. This approach was critical to our methodology, ensuring that every response’s coding could be independently cross-checked by at least one other researcher to reduce individual interpretive bias and establish consensus.

Responses were organised according to three decision points identified in the rapid review, which also informed the discussion guides and survey design. Within each decision point, qualitative data were analysed inductively to identify themes and behavioural patterns. Coding was reviewed by a second researcher, and discrepancies were discussed and resolved. Identified behaviours were then specified in terms of who performed them, what occurred, when and where they took place, and how they were enacted. Behaviours were subsequently coded deductively using the MAPPS framework to diagnose likely behavioural influences. This sequential inductive–deductive approach enabled systematic behavioural analysis to inform the development of targeted interventions.

Interview guides were then developed based on the three identified decision points, to capture and explore the different resulting behaviours from symptom recognition to treatment completion. Full interview guides are available in S1 Text.

### Quantitative methodology

**Fig 3:**
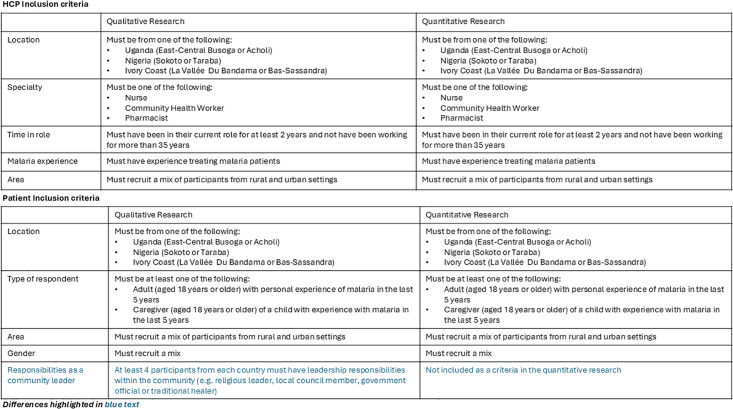
Qualitative and Quantitative Sample Comparison.

Strict inclusion and exclusion criteria, that were similar to the qualitative screening criteria, were also applied for the quantitative survey via a screening questionnaire (Fig 3). For healthcare professionals, inclusion criteria required them to be practising nurses, community health workers, or pharmacists who had been in their current role for at least two years and had experience treating malaria patients. For adults in the community, inclusion criteria required individuals to be adults aged 18 or above who either had suffered from malaria in the past five years, or were the parent/caregiver of a child who had experienced malaria in the past five years. A mix of genders and those in rural and urban settings were also recruited. Community leaders were not specifically recruited as part of the quantitative sample. Sample quotas were set to ensure a mix of respondents included in the sample; these sample quotas can be found in S5 Table.

The quantitative phase used convenience and snowballing sampling design to obtain a non-probability sample within each district (the limitations of use of these methods for a quantitative survey are mentioned in the Discussion section). Quantitative fieldwork was conducted between 15^th^ July 2024–4^th^ September 2024. The sampling frame was designed to ensure inclusion of each sub-group (adults, caregivers, nurses, community health workers, and pharmacists) from each district. The quantitative phase followed, with face-to-face, structured questionnaires taking approximately 30 minutes to complete. Written informed consent was obtained from all participants at the start of each questionnaire, before data collection commenced. The questionnaires were designed to validate and quantify findings from the qualitative phase, measuring the incidence and prevalence of identified barriers to effective malaria treatment. The questionnaire was structured using the three identified decision points to assess behaviours and their influences at each stage (S2 Text). The questionnaire was predominantly consistent for adults and caregivers, with some additional questions for each, to allow for the amalgamation of datasets during analysis. Data were collected using a structured questionnaire programmed onto tablet computers.

All quantitative analyses were conducted using SPSS. Cross-tabulations were performed by country, respondent type (adults, caregivers, HCP), and other relevant demographics to explore variations in responses. To analyse the categorical data and quantify the prevalence of identified barriers, independent samples t-tests were applied to compare proportions. Given the district-level sampling framework, inferential comparisons were specifically made between individual country data and the overall study mean, rather than directly between countries. These analyses aimed to validate the qualitative themes generated in the primary phase of the study. No deviations from the study protocol were made after ethical approvals were obtained.

### Data availability

Due to ethical restrictions, GDPR compliance, and the specific terms of the informed consent approved by our local ethics committees, the raw, individual-level underlying data cannot be shared publicly. The nature of the research concerning participants’ health constitutes sensitive patient information. Participants only provided consent for their data to be shared and published in aggregated, anonymised forms, and all such aggregated data supporting the findings of this study are available within the manuscript. The study received ethical approval under these conditions from the Sokoto and Taraba State Ministry of Health, Health Research Ethics Committee in Nigeria (Ref: SMH/1580/V.IV and TRSHREC/2023/024), the National Health and Life Sciences Ethics Committee (CNESVS) in Ivory Coast (Ref: 244–23/MSHPCMU/CNESVS-km), and The AIDS Support Organisation Research Ethics Committee (TASO) in Uganda (Ref: TASO-2023–322). All such aggregated data supporting the findings of this study are available within the manuscript. Researchers who meet the criteria for access to confidential data may send data access requests to these ethics committees, for which up to date contact information can be found on their websites.

## Results

### Final sample

#### Qualitative.

A total of n = 54 community participants were included in the qualitative study in selected districts in Uganda, Nigeria, and Ivory Coast, with n = 18 participants from each country (S3 Table). Participants comprised adults who had experienced malaria themselves, parents or primary guardians of children (from infant - teen) who had malaria, members of the broader caregiver network (grandparents, aunts, uncles), and community leaders. The sample included a mix of genders and was recruited from both urban and rural settings to ensure diverse representation.

N = 36 healthcare professionals participated in the qualitative study, with n = 12 from each country. Among the healthcare professionals, one third were nurses (n = 12), one third were community health workers (n = 12), and one third were pharmacists (n = 12) (S3 Table). The healthcare professionals were recruited from various health facilities in both urban and rural areas and had direct experience in treating malaria.

#### Quantitative.

A total n = 769 respondents completed the adult/caregiver survey (S4 Table)**.** 40% were recruited as ‘adults’ answering about themselves, and 60% were recruited as ‘caregivers’ answering about their experiences caring for children with malaria. 65% of adult/caregiver respondents were female, 67% lived in a rural setting, and mean age was 32.6 years (SD 10.4).

A total n = 532 healthcare providers completed the survey (S4 Table). 37% were nurses, 36% were community health workers, and 27% were pharmacists. 65% of HCPs were male, 59% practised in a rural setting, and mean years in practice was 7.9 (SD 5.7). All HCPs were required to have seen babies, infants (1–5 years old), and adult patients (18 years or older) in the last three months.

Findings are presented with reference to the MAPPS [19] framework, which distinguishes motivational, ability, processing, physical and social influences on behaviour.

## Theme 1: Barriers to timely access to care

### Decision point: “Shall I seek care?”

#### Qualitative findings.

Many adults and caregivers reported delaying seeking professional medical care upon experiencing symptoms that were indicative of malaria. Instead of promptly visiting a healthcare facility, they often waited to see if the symptoms subsided naturally or attempted self-treatment using over-the-counter medications or traditional remedies. This behaviour was influenced by a lack of familiarity with malaria symptoms, difficulty in recognising early symptoms and associated underestimation of potential severity. There was also evidence for a degree of misplaced self-confidence in managing the illness independently, demonstrated by this adult male respondent from Ivory Coast: *“[Where there] is some general fatigue, I’m going to cut some wood roots and drink them. There you go! It’s not at any time that I go to the hospital to be hospitalised there. "*

Similarly, caregivers, particularly those with multiple or older children, reported delaying seeking care. Physical environmental constraints such as competing work responsibilities, childcare duties, and financial limitations also contributed to delays in seeking specialist medical care. Conversely, first-time parents and caregivers of infants tended to seek professional care promptly. The child’s reaction combined with inability of young children to articulate their symptoms and heightened concern for their vulnerability appeared to influence urgency of immediate action.. A Community Health Worker from Ivory Coast explained: *“Now, since they don’t know how to speak, it’s serious. They’re there, they’re just crying, you don’t know where it hurts. Now, you’re the father, you’re worried, you don’t know what to do. He’s there, he can’t speak, he’s just crying. So if you don’t send him to the hospital, you can’t let anyone know.”*

#### Quantitative findings.

Adults and caregivers were aware of many of the symptoms that indicate malaria with the following symptoms being most frequently associated with the disease (**Table 1**); high fever (97%), vomiting (89%), headaches (89%), and shaking chills (79%). Included in the question were several ‘red herring’ answers to assess participant’s awareness of symptoms that could indicate malaria. These included change in skin tone (45%), loss of sense of smell (39%) and rash (25%) which were less associated with the disease.. 71% of adults and caregivers reported visiting a healthcare facility to receive medication the last time they suspected that their or their child’s symptoms could indicate malaria, albeit sometimes delayed. 57% of adults and caregivers reported going to a healthcare facility to get a malaria test, however, there was notable variation by country: in the selected districts in Uganda, seeking a test was significantly higher (74%, p < .001) while this was significantly lower in Nigeria (40%, p < .001).

**Table 1:** Access to care – Adults and Caregivers.

	Total n = 769	Uganda n = 250	Nigeria n = 252	Ivory Coast n = 267
**Actions taken after last suspected malaria case** ^1^				
Went to a healthcare facility to get medication	547 (71%)	196 (78%)*	166 (66%)	185 (69%)
Went to a healthcare facility to get a test	437 (57%)	184 (74%)***	101 (40%)***	152 (57%)
Treated my symptoms at home with traditional medicine	141 (18%)	10 (4%)***	47 (19%)	84 (31%)***
Treated symptoms at home with paracetamol I already had	112 (15%)	48 (19%)	38 (15%)	26 (10%)*
Treated symptoms at home with antimalarials I already had	71 (9%)	17 (7%)	47 (19%)***	7 (3%)***
Went to traditional healer for advice or treatment	63 (8%)	5 (2%)***	41 (16%)***	17 (6%)
Waited a few days for symptoms to go away	29 (4%)	10 (4%)	15 (6%)	4 (1%)**
Went to community leader for advice or treatment	25 (3%)	17 (7%)**	5 (2%)	3 (1%)*
**The first action prioritised (if performed multiple) after realising symptoms might indicate malaria**				
I waited a few days for the symptoms to go themselves	19 (2%)	8 (3%)	9 (4%)	2 (1%)
I went to a healthcare facility to get medication (including antimalarials)	44 (6%)	8 (3%)	17 (7%)	19 (7%)
I went to a healthcare facility to get a test to confirm malaria	214 (28%)	90 (36%)	49 (19%)	75 (28%)
I went to a traditional healer for advice	8 (1%)	2 (1%)	4 (2%)	2 (1%)
I went to a traditional healer for treatment	15 (2%)	2 (1%)	10 (4%)	3 (1%)
I went to a community leader for advice/treatment	11 (1%)	7 (3%)	3 (1%)	1 (0%)
I treated my symptoms at home with paracetamol I had already	69 (9%)	39 (16%)*	16 (6%)	14 (5%)
I treated my symptoms at home with antimalarials that I had already	26 (3%)	4 (2%)	18 (7%)	4 (1%)
I treated my symptoms at home with traditional medicine	53 (7%)	5 (2%)	18 (7%)	30 (11%)
**Symptoms that may indicate malaria** ^1^				
High fever	747 (97%)	234 (94%)*	252 (100%)*	261 (98%)
Vomiting	685 (89%)	226 (90%)	212 (84%)*	247 (93%)*
Headache	683 (89%)	228 (91%)	225 (89%)	230 (86%)
Loss of sense of taste	654 (85%)	211 (84%)	194 (77%)***	249 (93%)***
Shaking chills	606 (79%)	199 (80%)	169 (67%)***	238 (89%)***
Muscle pain	469 (61%)	183 (73%)***	116 (46%)***	170 (64%)
Nausea	455 (59%)	183 (73%)***	78 (31%)***	194 (73%)***
Profuse sweating	435 (57%)	131 (52%)	132 (52%)	172 (64%)*
Abdominal pain	376 (49%)	137 (55%)*	104 (41%)*	135 (51%)
Diarrhoea	362 (47%)	174 (70%)***	66 (26%)***	122 (46%)
Change in skin tone	347 (45%)	127 (51%)*	53 (21%)***	167 (63%)***
Loss of sense of smell	300 (39%)	127 (51%)***	84 (33%)*	89 (33%)*
Cough	275 (36%)	130 (52%)***	106 (42%)*	39 (15%)***
Rash	190 (25%)	82 (33%)**	41 (16%)**	67 (25%)
**Influences on whether to seek care** ^2^				
It is important to seek care for my malaria symptoms early to avoid complications in the future	694 (90%)	235 (94%)*	220 (87%)	239 (90%)
Seeking care from a healthcare professional will help me recover from malaria	674 (88%)	238 (95%)***	194 (77%)***	242 (91%)
I am confident that a healthcare professional can give me the right advice on what to do to treat malaria	665 (86%)	227 (91%)*	199 (79%)**	239 (90%)*
I feel scared if I get symptoms that could indicate malaria	623 (81%)	209 (84%)	186 (74%)**	228 (85%)
It is easy for me to get professional help for my malaria symptoms	610 (79%)	195 (78%)	180 (71%)**	235 (88%)***
It can be difficult to distinguish the symptoms of malaria from other conditions	384 (50%)	135 (54%)	95 (38%)***	154 (58%)*
In my community, it is more important to seek care for other diseases instead of malaria	330 (43%)	118 (47%)	100 (40%)	112 (42%)
The healthcare facility I would like to go to is too far away for me to consider it	290 (38%)	108 (43%)	94 (37%)	88 (33%)
In my community, seeking health advice from a traditional healer is preferred instead of going to the clinic	284 (37%)	69 (28%)**	100 (40%)	115 (43%)*
Paracetamol gets rid of my malaria symptoms, just as well as a different treatment I could get from a healthcare facility	258 (34%)	54 (22%)***	66 (26%)**	138 (52%)***
My work and/or childcare responsibilities make it difficult for me to go to a healthcare facility quickly	214 (28%)	63 (25%)	55 (22%)*	96 (36%)**

1 Question was ‘multi-select’, therefore % will total over 100%

2 ‘ Top 2 Box ’ agreement shown, e.g., % of respondents selecting 1-2 on a 1-5 scale

* p < .05 **p < .01 ***p < .001

90% of adults and caregivers were motivated to seek care when they suspected malaria symptoms to avoid complications. 88% agreed that seeking care from a healthcare professional would help them recover from malaria, although agreement with this statement was significantly lower in Nigeria districts (70%, p < .001). The two top influences on delays in seeking professional care were ‘it can be difficult to distinguish the symptoms of malaria from other conditions’ (50%), and ‘it is more important to seek care for other diseases instead of malaria’ (43%). 18% of adults and caregivers reported treating symptoms at home with traditional medicine, although this was significantly higher in the Ivory Coast districts (31%, p < .001) and significantly lower in the Uganda districts (4%, p < .001). Approximately 15% of adults and caregivers reported treating symptoms at home with paracetamol, and 34% agreed that paracetamol could get rid of malaria symptoms just as well as different treatments they could get from a healthcare facility. Agreement with the latter statement was significantly higher in Ivory Coast districts (52%, p < .001). Out of all respondents who did visit a healthcare facility when they suspected malaria, only 28% said that this was the first action they took.

Taken together, the qualitative and quantitative findings suggest that delayed care-seeking is influenced by incomplete symptom recognition, misplaced confidence in self-management, perceived adequacy of home remedies or paracetamol, and practical and social constraints on accessing facilities. In our MAPPS-based coding, these influences were classified as ability, motivation, physical and social barriers to timely access to care (Table 7).

### Decision point: “Where shall I seek care?”

#### Qualitative findings.

It was reported that a common cultural practice was to seek advice from trusted community or religious leaders in the first instance which might delay appropriate medical treatment.. Some individuals preferred to seek care from pharmacies rather than visiting hospitals or clinics. Pharmacies were often considered more accessible and affordable, offering quick solutions without the need for formal medical appointments, which might involve more time and unpredictable costs. An adult male from Nigeria highlighted this preference: *“If you go to the hospital, the doctor will diagnose and run some tests on you and prescribe injections for you, but when you go to a pharmacist, he will only ask you to explain what is wrong with you; then he will prescribe drugs based on experience, and you just buy the right drugs.”*

On the other hand, those who considered the symptoms to be especially severe, choose to visit hospitals or clinics directly, recognising the importance of professional diagnosis and comprehensive treatment. This decision is often associated with having positive experiences with healthcare facilities previously.

#### Quantitative findings.

86% of the adults and caregivers agreed that they were confident that going to a qualified HCP for advice was the best option when experiencing malaria symptoms and 82% believed that it was worth the time and cost to travel to a hospital or clinic to get treatment (Table 2). However, agreement with both statements was significantly lower in the selected districts in Nigeria (78%, p < .01 and 69%, p < .05, respectively). 74% stated that community leaders encourage them to seek professional care immediately if they suspect malaria. However, 54% agreed that most people in the community self-treated rather than going to a hospital or clinic for malaria treatment. This was significantly lower in the districts in Uganda (47%, p < .05) and higher in those in Nigeria (60%, p < .05). Viewed together, these findings suggest that place of care is determined not only by formal preferences for professional advice but also by the influence of local gatekeepers, the norms of self treatment and the greater proximity and affordability of pharmacies. In our MAPPS coding, these influences were treated as part of the wider pattern of social and physical barriers and enablers to timely access shown in Table 7.

**Table 2:** Where to seek care – Adults and caregivers.

	Total n = 769	Uganda n = 250	Nigeria n = 252	Ivory Coast n = 267
**Influences on where to seek care** ^1^				
I am confident that going to a qualified HCP for advice is the best option when experiencing malaria symptoms	661 (86%)	229 (92%)**	197 (78%)**	235 (88%)
I believe it is worth the time and cost to travel to the hospital or clinic to get treated for malaria	629 (82%)	217 (87%)*	175 (69%)***	237 (89%)**
There is an accessible hospital/clinic close to my home that I can go to if experiencing malaria symptoms	590 (77%)	184 (74%)	174 (69%)**	232 (87%)***
Community leaders encourage us to seek professional medical care immediately if we suspect malaria rather than waiting	567 (74%)	201 (80%)*	165 (65%)**	201 (75%)
There are transport options available to me to go to the hospital or clinic if I/my child develop malaria symptoms	495 (64%)	152 (61%)	139 (55%)**	204 (76%)***
I set aside money in case I need to cover the cost of treatment if I/my child does get sick	433 (56%)	125 (50%)*	121 (48%)*	187 (70%)***
Most people in my community self-treat themselves or their children rather than going to a hospital or clinic for malaria treatment	417 (54%)	117 (47%)*	150 (60%)*	150 (56%)
Pharmacies are much closer to my home than the nearest hospital or clinic	382 (50%)	81 (32%)***	174 (69%)***	127 (48%)
It’s quicker to go to the pharmacy than a hospital or clinic when experiencing malaria symptoms	365 (47%)	70 (28%)***	155 (62%)***	140 (52%)
It’s cheaper to go to the pharmacy than a hospital or clinic when experiencing malaria symptoms	325 (42%)	58 (23%)***	126 (50%)**	141 (53%)***
I am used to describing malaria symptoms to the pharmacist and purchasing treatment without getting tested first	317 (41%)	56 (22%)***	137 (54%)***	124 (46%)
I trust the pharmacist’s advice on which malaria medication to take so I don’t feel the need to see a doctor first	304 (40%)	50 (20%)***	113 (45%)	141 (53%)***
I can get malaria medication at a lower cost from the pharmacy compared to the consultation and prescription fees at a hospital or clinic	299 (39%)	52 (21%)***	111 (44%)	136 (51%)***
I prefer going to the pharmacy for malaria because I can get the medication right away without a prescription	281 (37%)	48 (19%)***	128 (51%)***	105 (39%)
I prefer to seek care from a community leader first, before seeking care from a trained HCP	249 (32%)	83 (33%)	75 (30%)	91 (34%)
I believe a pharmacist can treat malaria symptoms better than an HCP at a hospital or clinic	231 (30%)	30 (12%)***	87 (35%)	114 (43%)***

1 ‘ Top 2 Box ’ agreement shown, e.g., % of respondents selecting 1-2 on a 1-5 scale

* p < .05 **p < .01 ***p < .001

## Theme 2: Inconsistent diagnostic testing

### Decision point: “Shall I get tested?” / “Shall I test this patient?”

#### Qualitative findings.

Most adults and caregivers who visited healthcare facilities readily accepted the advice of the HCP and underwent diagnostic testing for malaria when this was defined as the best course of action. Most understood that testing is essential for accurate diagnosis and appropriate treatment. This awareness reflected a recognition of the importance of confirming malaria rather than relying solely on symptom presentation. A male caregiver from Ivory Coast reflected on advice he had previously received from an HCP: *“He said that the fact that I vomit or I have a strong migraine doesn’t necessarily mean that I have malaria because perhaps there are diseases that can have the same symptoms as malaria. So, the best solution is to do the test.”*

However, some individuals declined testing for several reasons. Confidence in self-diagnosis led some to believe they could accurately identify the illness without a test, often based on past experiences with malaria. Financial barriers also played a role; testing may have involved additional expenses, the affordability of which could outweigh the importance of testing. Additionally, many pharmacies and informal healthcare providers often did not offer testing services which precluded testing through these providers. Therefore, individuals may have requested treatment or purchased medication over the counter without confirmation of diagnosis.

Most healthcare professionals reported conducting malaria tests when they suspected malaria based on known symptoms such as high fever, joint aches, abdominal pain, and vomiting. Rapid Diagnosis Tests (RDTs) were commonly used given their availability and quick results. Microscopy, considered the gold standard, was less available and thus used less frequently. However, a minority of healthcare professionals stated they prescribed antimalarials without testing, despite having tests available. Some preferred to rely on their clinical judgement, particularly when they believed the signs of malaria were obvious. Some also questioned the reliability of RDTs.

In some instances, healthcare professionals did not test patients because RDTs were out of stock or not accessible. As a result, patients were sometimes treated based on clinical symptoms without confirmation. Most healthcare professionals reported prescribing antimalarials promptly following a positive test. However, some healthcare professionals administered antimalarials even after negative test results, driven by lack of confidence in test accuracy or strong clinical suspicion that overruled the test result. A female pharmacist from Ivory Coast explained: “*When the test is negative and we strongly suspect a case of malaria, we still prescribe malaria treatment, as the test is not 100% reliable. And we advise going to the hospital after three days if the disease doesn’t go away.*”

In rare situations, healthcare professionals gave intermittent treatment before referral after a positive test due to resource limitations or referral protocols. A female pharmacist from Nigeria explained: *“If the malaria is severe, we give them pre-referral medication then we refer them to the appropriate health facility.”*

#### Quantitative findings.

64% of adults and caregivers reported that their healthcare professional offered them a test the last time they had malaria, though this was significantly higher in the Ivory Coast districts (85%, p < .001) and significantly lower in the Nigerian districts (37%, p < .001) (Table 3). 19% reported having to ask a healthcare professional for a test themselves (significantly higher in Nigeria at 33%, p < .001, and lower in Ivory Coast at 5%, p < .001) and 17% reported not receiving a test at all for their last suspected malaria case (significantly higher in Nigeria at 31%, p < .001

**Table 3:** Testing – adults and caregivers.

	Total n = 769	Uganda n = 250	Nigeria n = 252	Ivory Coast n = 267
**Experience with testing / diagnosis at last malaria case**				
My HCP offered a test to confirm my/my child’s diagnosis	491 (64%)	170 (68%)	93 (37%)***	228 (85%)***
I asked an HCP for a test to confirm my/ my child’s diagnosis	149 (19%)	54 (22%)	82 (33%)***	13 (5%)***
My/my child’s diagnosis was not confirmed by a test	129 (17%)	26 (10%)**	77 (31%)***	26 (10%)**
**Attitudes towards testing** ^1^				
I know where to go to get a malaria test	694 (90%)	241 (96%)**	213 (85%)*	240 (90%)
I am confident getting myself/my child tested will help receive the treatment	683 (89%)	243 (97%)***	189 (75%)***	251 (94%)**
It is important to get tested to confirm malaria diagnosis	679 (88%)	240 (96%)***	190 (75%)***	249 (93%)*
I believe that getting myself/my child tested will save me time in the future	623 (81%)	221 (88%)**	163 (65%)***	239 (90%)***
I believe getting myself/my child testing is the only way to confirm malaria	622 (81%)	228 (91%)***	154 (61%)***	240 (90%)***
Malaria testing is easy to get at a healthcare facility near me	595 (77%)	204 (82%)*	149 (59%)***	242 (91%)***
I believe that getting myself/my child tested will save me money in the future	592 (77%)	215 (86%)**	141 (56%)***	236 (88%)***
The process of getting tested for malaria is quick	578 (75%)	191 (76%)	145 (58%)***	242 (91%)***
I can afford the cost of a malaria test	522 (68%)	145 (58%) **	143 (57%)***	234 (88%)***

1 ‘ Top 2 Box ’ agreement shown, e.g., % of respondents selecting 1-2 on a 1-5 scale

* p < .05 **p < .01 ***p < .001

90% of adults and caregivers knew where to get a malaria test and 89% were confident that getting a test would ensure they received the right treatment (however, agreement with the latter was significantly lower in Nigeria districts at 75%, p < .001). 88% agreed that getting tested was important in confirming a malaria diagnosis and 81% agreed that getting tested would save them time in the future. Again, agreement with both statements was significantly lower in Nigerian districts (75%, p < .001 and 65%, p < .001, respectively). Approximately two-thirds (68%) agreed that they could afford the cost of a malaria test.

86% of HCPs stated it was important to act quickly if they suspected a patient had malaria, only 67% stated that administering RDTs was something they always do with their patients who are experiencing malaria symptoms (Table 4). This was significantly higher in districts in Uganda (78%, p < .01) and lower in Nigeria (60%, p < .05). 66% agreed that if they were unable to confirm their patient’s diagnosis with a test that they may end up spending more on their treatment and 61% agreed that if they were unable to confirm their patient’s diagnosis with a test, their health might be negatively impacted. 28% reported that their practice had run out of RDTs on occasion in the past. Overall, 23% stated that other HCPs in their facility do not conduct RDTs before prescribing treatment; this was significantly higher in districts in Nigeria (42%, p < .001). 20% agreed that they do not need an RDT before treating a patient to know they are giving them the right treatment, again this was significantly higher in Nigeria (28%, p < .001). 63% of healthcare professionals agreed that it was both theirs and the adult/caregiver’s responsibility to ensure a patient receives a test to diagnose malaria; significantly higher in Nigeria districts at 70% (p < .05). 27% of HCPs believed it was their sole responsibility to ensure patients received a malaria test; this was significantly higher in districts in Uganda at 41% (p < .001).

**Table 4:** Testing – HCPs.

	Total n = 532	Uganda n = 120	Nigeria n = 200	Ivory Coast n = 212
**Attitudes towards testing before treating** ^1^				
It is important to act quickly if I suspect a patient has malaria	455 (86%)	106 (88%)	172 (86%)	177 (83%)
Administering RDTs is something I always do with all my patients who are experiencing malaria symptoms	358 (67%)	93 (78%)**	119 (60%)*	146 (69%)
If I am unable to confirm my patient’s diagnosis with a test, they might end up spending more on their treatment	349 (66%)	90 (75%)*	121 (60%)	138 (65%)
Diagnostic tests conducted in the lab are more reliable than RDTs	345 (65%)	81 (68%)	115 (58%)*	149 (70%)
If I am unable to confirm my patient’s diagnosis with a test, their health might be negatively impacted	323 (61%)	86 (72%)*	116 (58%)	121 (57%)
If I am unable to confirm my patient’s diagnosis with a test, it is more difficult for me know what treatment to give them	308 (58%)	83 (69%)*	104 (52%)	121 (57%)
Patients in my community do not expect to be tested for malaria when presenting with symptoms	188 (35%)	24 (20%)***	96 (48%)***	68 (32%)
I try to follow suggested guidelines, but it is not always possible	151 (28%)	36 (30%)	66 (33%)	49 (23%)
My practice has run out of RDTs on occasion in the past	150 (28%)	29 (24%)	52 (26%)	69 (33%)
Other HCPs in my facility do not conduct RDTs before prescribing treatment	120 (23%)	17 (14%)*	84 (42%)***	19 (9%)***
I don’t need an RDT before treating a patient to know I am giving them the right treatment	108 (20%)	7 (6%)***	56 (28%)**	45 (21%)
I do not discuss testing decisions with my colleagues	108 (20%)	19 (16%)	34 (17%)	55 (26%)*
I need some support in interpreting the results of malaria RDTs	99 (19%)	14 (12%)*	60 (30%)***	25 (12%)**
I am not familiar with the different malaria test types	91 (17%)	36 (30%)***	28 (14%)	27 (13%)
It can be difficult to identify when an RDT is inaccurate or inconclusive	91 (17%)	24 (20%)	28 (14%)	39 (18%)
RDTs for malaria are unreliable	85 (16%)	17 (14%)	23 (12%)	45 (21%)
I don’t always trust the quality and reliability of the RDT kits supplied to my facility	84 (16%)	22 (18%)	24 (12%)	38 (18%)
When the clinic is busy, it is difficult to test before diagnosis	83 (16%)	13 (11%)	43 (22%)*	27 (13%)
The RDTs are not stored properly at my facility which means their accuracy and performance are not maintained	79 (15%)	14 (12%)	32 (16%)	33 (16%)
The instructions on RDTs are not always clear and easy to follow	76 (14%)	14 (12%)	28 (14%)	34 (16%)
I find it challenging to administer malaria RDTs to patients	53 (10%)	6 (5%)*	26 (13%)	21 (10%)
I have not received training on the procedures for administering malaria RDTs	47 (9%)	9 (8%)	20 (10%)	18 (8%)
I sometimes forget to test	37 (7%)	5 (4%)***	26 (13%)**	6 (3%)*
**Perceived responsibility to ensure patients receive a diagnostic test**				
It is my responsibility to ensure patients receive a test to diagnose malaria	145 (27%)	49 (41%)**	29 (14%)***	67 (32%)
It is both the patient/caregiver and my responsibility as a HCP to ensure the patient receives a test to diagnose malaria	333 (63%)	65 (54%)*	140 (70%)*	128 (60%)
It is solely the patient/caregiver’s responsibility to ensure the patient receives a test to diagnose malaria	54 (10%)	6 (5%)*	31 (16%)*	17 (8%)
*Base: all HCPs who prescribe antimalarials*	**Total n = 491**	**Uganda n = 120**	**Nigeria n = 199**	**Ivory Coast n = 172**
**Most recent experience testing and treating a patient**				
***Infants and neonates <5 kg***				
I gave them antimalarial treatment after they received a positive test result	293 (60%)	86 (72%)**	118 (59%)	89 (52%)*
I gave them antimalarial treatment after they received a negative test result	90 (18%)	11 (9%)**	39 (20%)	40 (23%)
I gave them antimalarial treatment even though they did not do a test	80 (16%)	11 (9%)*	33 (17%)	36 (21%)
Other	28 (6%)	12 (10%)	9 (5%)	7 (4%)
***Infants and neonates 5 kg or more***				
I gave them antimalarial treatment after they received a positive test result	303 (62%)	96 (80%)***	120 (60%)	87 (51%)**
I gave them antimalarial treatment after they received a negative test result	83 (17%)	9 (8%)**	37 (19%)	37 (22%)
I gave them antimalarial treatment even though they did not do a test	89 (18%)	15 (12%)	34 (17%)	40 (23%)
Other	16 (3%)	0 (0%)	8 (4%)	8 (4%)
***Children 1–5 years***				
I gave them antimalarial treatment after they received a positive test result	318 (65%)	97 (81%)***	111 (56%)*	110 (64%)
I gave them antimalarial treatment after they received a negative test result	63 (13%)	5 (4%)**	37 (19%)*	21 (12%)
I gave them antimalarial treatment even though they did not do a test	74 (15%)	17 (14%)	33 (17%)	24 (14%)
Other	36 (7%)	1 (1%)**	18 (9%)	17 (10%)
***Children 6–12 years***				
I gave them antimalarial treatment after they received a positive test result	299 (61%)	93 (78%)***	109 (55%)	97 (56%)
I gave them antimalarial treatment after they received a negative test result	59 (12%)	6 (5%)*	30 (15%)	23 (13%)
I gave them antimalarial treatment even though they did not do a test	91 (19%)	13 (11%)*	41 (21%)	37 (22%)
Other	42 (9%)	8 (7%)	19 (10%)	15 (9%)
***Teenagers 13–17 years***				
I gave them antimalarial treatment after they received a positive test result	288 (59%)	80 (67%)	104 (52%)*	104 (60%)
I gave them antimalarial treatment after they received a negative test result	74 (15%)	12 (10%)	33 (17%)	29 (17%)
I gave them antimalarial treatment even though they did not do a test	95 (19%)	14 (12%)*	54 (27%)**	27 (16%)
Other	34 (7%)	14 (12%)*	8 (4%)	12 (7%)
***Adults >18 years***				
I gave them antimalarial treatment after they received a positive test result	273 (56%)	82 (68%)**	99 (50%)	92 (53%)
I gave them antimalarial treatment after they received a negative test result	66 (13%)	8 (7%)*	30 (15%)	28 (16%)
I gave them antimalarial treatment even though they did not do a test	114 (23%)	13 (11%)**	59 (30%)*	42 (24%)
Other	38 (8%)	17 (14%)*	11 (6%)	10 (6%)

1 ‘ Top 2 Box ’ agreement shown, e.g., % of respondents selecting 1-2 on a 1-5 scale

* p < .05 **p < .01 ***p < .001

Over half of HCPs stated that during their most recent experience with a patient, they tested their patients with an RDT before offering an antimalarial treatment. Patterns of testing were generally claimed to be consistent across age groups (for example, testing with an RDT before offering antimalarials was 60% amongst infants and neonates weighing <5 kg patients, 62% amongst infants and neonates weighing >5 kg, 65% amongst children 1–5 years, 61% children 6–12 years, 59% amongst teenagers 13–17 years, 56% for adults >18 years).

Taken as a whole, the qualitative and survey data suggest that inconsistent use of diagnostic testing reflects distinct but related influences on adults/caregivers and on HCPs behaviour. For adults and caregivers, the key barriers related to concerns (real or anticipated) about cost of tests, limited understanding of why an objective test is necessary, and reluctance to ask for a test from a trained professional who had not actively suggested testing; in our MAPPS-based coding these were classified as processing, physical, ability and social influences on consistent diagnostic testing (Table 7). For HCPs, inconsistency in testing was driven more by reliance on clinical experience over diagnostic tests, constraints of time and test availability, and, in some cases, doubts about the added value of testing; these were coded as processing and physical influences in MAPPS, reflecting both decision-making preferences and structural limitations on routine test use (Table 7).Survey results by HCP type are provided as S1 Table.

## Theme 3: Non-adherence to treatment

### Decision point: “Shall I take the full dose?”/ “How should I engage with this patient? "

#### Qualitative findings.

Many adults and caregivers reported not completing the full prescribed course of antimalarial medication. They reported often discontinuing treatment once symptoms improved, believing that they are fully recovered, or they saved the remaining medication for future use to reduce costs. Many understood the importance of finishing treatment but prioritised the material need of saving treatment for another time. Caregivers also faced challenges in ensuring that children completed their treatment, particularly with adolescents who may resist taking medication consistently. A female caregiver from Uganda shared: *“Sometimes [teenagers] refuse to take in medicine, or sometimes they don’t finish doses by maybe taking half, so malaria hides in their bodies, and when it comes back, it becomes stronger which makes it severe.”*

Several factors influenced this incomplete adherence. Amongst some, there was a belief that symptom resolution equates to full recovery, making further medication seem unnecessary. This misconception led patients and caregivers to discontinue treatment prematurely. In addition, the unpleasant taste or large size of tablets made them difficult to ingest, especially for children, discouraging consistent use. Financial constraints also led individuals to ration medication, either by skipping doses or saving some for future use. Forgetfulness also played a role in non-adherence. Difficulty incorporating medication schedules into daily routines due to work, school, or other commitments reportedly resulted in missed doses. Finally, social influence was shown to affect adherence as some reported observing others who did not complete treatment without apparent adverse effects. This reinforced a belief that non-adherence may not impact treatment outcomes making incomplete adherence seem acceptable within the community.

HCPs reported providing instructions on dosage and emphasising the importance of completing the medication course. They also advised on preventive measures to reduce the risk of reinfection. A female nurse from Nigeria stated: *“There are three key things we tell our patients: to be compliant with the drugs, to sleep under mosquito-treated nets, and to clean all stagnant water around your environment to stop it coming back.”* Despite these efforts, there was a disconnection between what HCPs claimed they were communicating and what patients recalled or implemented. This gap indicated that current communication strategies are not always effective in supporting adherence.

HCPs often assumed patient compliance without additional reinforcement. As one male nurse from Nigeria observed: “*When they get better, we know they took their medication and we have followed up appointment with them*,” implying that feeling better is proof of adherence. Another female community healthcare worker from Nigeria mentioned: “*when you a give patient drugs and he goes to take them and does not come back you will assume he has finished taking them and he is okay, and if he is not okay you will be waiting for him to come back*,” which also assumed adherence despite no proven evidence of adherence.

HCPs generally provided patients with basic instructions on how to take their medication. For example, a female nurse from nurse from Uganda stated: *“I will tell that these are the prescribed drugs...Use them how they have been prescribed to you.”* However, as some patients also acknowledged, conditions for communications could be difficult, particularly considering high patient volumes and constrained resources, as illustrated by an adult male from Uganda: *“While in busy health centres, healthcare providers may not have as much time for extensive discussions due to the high volume of patients”.*

#### Quantitative findings.

85% of adults and caregivers stated that they were aware of the reasons for needing to take the full course of treatment and 84% agreed they knew exactly how often and when they should take their treatment (Table 5). However, this was significantly lower in districts in Nigeria (73%, p < .001 and 74%, p < .001, respectively). 75% agreed that they knew they should take their antimalarial treatment even when their symptoms disappear (again significantly lower in Nigeria at 69%, p < .05). 33% agreed that it was fine to stop treatment when symptoms are gone as this meant the disease is gone; this was significantly lower in districts in Uganda at 15% (p < .001) and higher in districts in Ivory Coast (47%, p < .001). 30% also agreed that stopping malaria treatment early meant they could save money for treatment next time.

**Table 5:** Adherence – Adults/caregivers.

	Total n = 769	Uganda n = 250	Nigeria n = 252	Ivory Coast n = 267
**Reasons for adherence/non-adherence to antimalarials** ^1^				
I know the reasons for needing to take the full course of treatment	657 (85%)	225 (90%)*	183 (73%)***	249 (93%)***
I know exactly how often and when I should take my antimalarial treatment	649 (84%)	225 (90%)**	187 (74%)***	237 (89%)*
People I know believe one should take all of their antimalarial treatment, even when their symptoms disappear	573 (75%)	186 (74%)	174 (69%)*	213 (80%)
The taste of antimalarial pills makes them difficult to take	515 (67%)	165 (66%)	133 (53%)***	217 (81%)***
Malaria treatment is expensive	466 (61%)	185 (74%)***	131 (52%)**	150 (56%)
People I know have stopped taking antimalarial treatment early and still recovered	336 (44%)	88 (35%)**	111 (44%)	137 (51%)*
Once my malaria symptoms are gone, there is nothing left for the treatment to do	317 (41%)	53 (21%)***	113 (45%)	151 (57%)***
It can be difficult to remember to take all of the doses of antimalarial treatment	317 (41%)	97 (39%)	76 (30%)***	144 (54%)
It is fine to stop treatment when my symptoms are gone, because it means that the disease is gone	255 (33%)	38 (15%)***	91 (36%)	126 (47%)***
Stopping malaria treatment early means that I can save money for treatment next time	232 (30%)	49 (20%)**	72 (29%)	111 (42%)***
Stopping malaria treatment early means that I don’t have to see an HCP the next time I have malaria	221 (29%)	40 (16%)***	71 (28%)	110 (41%)***
**How treatment was taken during last case of malaria**				
I took all of my antimalarials	608 (79%)	226 (90%)***	176 (70%)**	206 (77%)
I took some of my antimalarials	89 (12%)	19 (8%)*	34 (13%)	36 (13%)
I did not take any antimalarials	66 (9%)	4 (2%)***	40 (16%)***	22 (8%)
Don’t know	6 (1%)	0 (0%)	2 (1%)	3 (1%)
*Base: those who only took some of antimalarials last time they had malaria*	**Total n = 89**	**Uganda n = 19**	**Nigeria n = 34**	**Ivory Coast n = 36**
**Which of the following best describes why you did not take all your medication?** ^2^				
I stopped taking it/giving it to my child when my/my child’s symptoms disappeared	50 (56%)	13 (68%)	22 (65%)	15 (42%)
I/my child stopped taking it because I/my child did not like the taste	21 (24%)	3 (16%)	10 (29%)	8 (22%)
I/my child forgot to take it	11 (12%)	2 (11%)	2 (6%)	7 (19%)
I wanted to save some treatment for next time	9 (10%)	3 (16%)	3 (9%)	3 (8%)
I/my child stopped taking it because it made me/my child feel unwell	8 (9%)	4 (21%)	0 (0%)	4 (11%)
I stopped taking it/giving to my child because it did not help relieve my/my child’s symptoms	3 (3%)	1 (5%)	1 (3%)	1 (3%)

1 ‘ Top 2 Box ’ agreement shown, e.g., % of respondents selecting 1-2 on a 1-5 scale

2 Question was ‘multi-select’, therefore % will total over 100%

* p < .05 **p < .01 ***p < .001

When asked how treatment was taken during their most recent bout of malaria, 79% of adults and caregivers reported taking all of their antimalarials; this was significantly higher in districts in Uganda (90%, p < .001) and significantly lower in districts in Nigeria (70%, p < .01). Of those who did not take all their medication, 56% reported that they/their child stopped taking their medication when their/their child’s symptoms disappeared and 24% stated they/their child stopped taking it because of the taste, with no significant differences observed across the three countries.

75% of HCPs agreed that it was both their and the patient/caregiver’s responsibility to ensure the patient took the full course of treatment, whilst 18% believed it was solely the patient/caregiver’s responsibility (Table 6). 94% of healthcare professionals felt confident in their ability to explain reasons for completing treatment to their patients and 91% believed that having a discussion with a patient about their treatment made a difference to whether they take it correctly. However, 18% agreed they lacked the time to have conversations with their patients about the importance of taking all their treatment.

**Table 6:** Adherence – HCPs.

	Total n = 532	Uganda n = 120	Nigeria n = 200	Ivory Coast n = 212
**Perceived responsibility of patient in adherence to treatment**				
It is solely the patient/their caregiver’s responsibility to ensure they take the full course of treatment	97 (18%)	18 (15%)	50 (25%)*	29 (14%)
It is both the patient/their caregiver’s and my responsibility as their HCP to ensure they take the full course of treatment	400 (75%)	98 (82%)	144 (72%)	158 (75%)
It is solely my responsibility to ensure patients take their full course of treatment	35 (7%)	4 (3%)	6 (3%)*	25 (12%)*
**Attitudes toward discussing adherence with patients** ^1^				
I know the reasons for needing to complete the full course of treatment	502 (94%)	116 (97%)	183 (92%)	203 (96%)
I am confident in my ability to explain reasons for completing treatment to my patients	501 (94%)	116 (97%)	183 (92%)	202 (95%)
I have seen my patients fully recover after giving them or prescribing treatment	496 (93%)	113 (94%)	176 (88%)*	207 (98%)**
I am confident in my ability to explain treatment dosage to my patients	494 (93%)	111 (92%)	180 (90%)	203 (96%)
My discussion with the patient about their treatment makes a difference to whether they take it correctly	482 (91%)	109 (91%)	170 (85%)**	203 (96%)**
My colleagues and I discuss ways to encourage more patients to adhere to treatment	473 (89%)	98 (82%)*	169 (84%)*	206 (97%)***
The HCPs I know have conversations about treatment dosage with patients	470 (88%)	105 (88%)	164 (82%)*	201 (95%)**
The HCPs I know have conversations about completing treatment with patients	465 (87%)	91 (76%)**	172 (86%)	202 (95%)***
I find it easy to respond to most patient questions and concerns on taking or completing treatment	463 (87%)	109 (91%)	151 (76%)***	203 (96%)***
It is solely my role to encourage patients to adhere to treatment	440 (83%)	66 (55%)***	171 (86%)	203 (96%)***
I use supporting materials (e.g., leaflets) to support my conversations on adherence to treatment with patients	339 (64%)	66 (55%)*	125 (62%)	148 (70%)
It can be difficult to explain the importance of adherence to treatment to patients	164 (31%)	28 (23%)*	50 (25%)	86 (41%)**
I lack the time to have conversations with my patients about the importance of taking all of their treatment	95 (18%)	17 (14%)	25 (12%)*	53 (25%)*

1 ‘ Top 2 Box ’ agreement shown, e.g., % of respondents selecting 1-2 on a 1-5 scale

* p < .05 **p < .01 ***p < .001

Across both data sources, non-adherence among adults and caregivers appeared to be driven less by lack of information and more by expectations that treatment can safely be stopped once symptoms resolve, practical difficulties in taking unpleasant medication and fitting dosing into daily routines, and the perception that stopping early is common and acceptable. In our MAPPS-based coding for ‘correct adherence to treatment’, these patient-side influences were classified under motivation - outcome expectations, physical, processing and ability - routines, with social norms also reinforcing early cessation (Table 7). The HCP findings suggest that providers are aware they can influence adherence through communication and follow‑up, but that their ability to do so is constrained by limited consultation time, assumptions of compliance, and a social expectation of shared responsibility between provider and patient; we interpret these HCP-related factors as part of the wider context that can either support or undermine the patient adherence behaviours summarised in Table 7.

Survey results by HCP table are available in S2 Table.

## Discussion

Despite significant efforts over the past decades to control malaria through the widespread use of ACTs, malaria remains a pervasive health challenge in sub-Saharan Africa. This study explored the behavioural barriers to effective malaria treatment in select districts of Uganda, Nigeria, and Ivory Coast, identifying influences that impede timely access to care and adherence to antimalarial medication. By applying the MAPPS [19] behavioural science framework, which considered Motivation, Ability, Physical, Processing and Social dimensions, we identified specific influences on malaria treatment behaviours. This framework allowed us to move beyond a description of attitudes and practices to a structured diagnosis of where, and in what form, the behavioural barriers occur across the treatment pathway. This further enabled us to identify behaviours that, if addressed through targeted interventions, could lead to more timely access and adherence to antimalarial treatment. Below we describe each of the key behaviours in detail, and present suggested relevant intervention guidance (**Table 7**).

**Table 7:** Behaviours identified in research, MAPPS dimension and relevant intervention guidance.

Behaviour	Group	Barrier to behaviour	Relevant MAPPS dimension / sub-dimension	Intervention guidance
Timely access to care	Adults / caregivers	Familiarity with disease, misplaced self-efficacy and confidence in ability to manage	Motivation – self efficacy	Communication of risk associated with delayed care
		Difficulty recognising range and combination of symptoms that may indicate malaria	Ability	Clarification/checklist of the most common symptoms as a prompt to seek care and testing without delay
		Social norm to delay access	Social	Encouraging people to take immediate action by emphasising that prompt action is socially expected and supported.
		Access to affordable and geographically convenient care	Physical	Likely to require systemic intervention
Consistent diagnostic testing	HCPs	Confidence in clinical experience over diagnostic tests	Processing	Emphasising potential negative consequences of not testing
		Time to test and availability of tests	Physical	Assisting with strengthening the supply chain, emphasis of rapid nature of test vs consequences of unconfirmed diagnosis
	Adults / caregivers	Expectation of high cost of test	Processing / Physical	Clear communication of cost and value of test
		Unaware of importance of objective test	Ability	Communication of certainty provided by objective test
		Feel unable to proactively request / demand a test from a trained professional	Social	Promote social acceptability of asking for a test
Correct adherence to treatment	Adults / caregivers	Belief that symptom resolution equates to full recovery, making further medication seem unnecessary	Motivation – Outcome Expectations	Educating on the importance of completing treatment courses; address misconceptions about recovery upon symptom resolution.
		Unpleasant taste or large size of tablets, making them difficult to ingest, especially for children	Physical	Improve medication palatability and formulation.
		Forgetfulness and difficulty incorporating medication schedules into daily routines	Processing & Ability – Routines	Simplify treatment regimens; implement reminders and support tools for adults/parents to aid adherence (e.g., follow-up messages, community health worker visits).
		Observing others who do not complete treatment without adverse effects, reinforcing belief that non-adherence is acceptable	Social	Social strategies to shift community perceptions about adherence; reinforce the importance of completing treatment.

### Barriers to timely access to care

Prompt access to antimalarial treatment is essential because it allows for effective management of malaria possibly preventing progression to severe and potentially life-threatening stages of the disease, as well as reducing potential for resistance and onward transmission [22,23]. In our study, one-third of respondents reported not immediately seeking care from a healthcare facility when experiencing symptoms. Our data suggested that the delay in seeking care from a medical facility is influenced by a number of interconnected factors including previous experience and what might be considered misplaced self-efficacy.

Self-efficacy, defined as “an individual’s belief in their capacity to execute behaviours necessary to produce specific outcomes”, [24] is important to understand in the context of any behaviour change process, including accessing and adhering to malaria treatment. As many individuals have experienced malaria many times over their life, this may lead to a high level of self-efficacy and overconfidence bias due to familiarity with, and confidence in, self-managing symptoms. In this study, some respondents articulated a belief in their ability to treat suspected symptoms of malaria effectively with paracetamol or alternative remedies. This misplaced self-efficacy limited motivation to seek medical care sooner and was coded as a motivational barrier using the MAPPS framework.

The early symptoms of malaria can be non-specific and easily mistaken for other common illnesses. This misunderstanding, owing to lack of full knowledge of the range and possible combination of symptoms also appeared to limit the timely seeking of professional care. This was coded as an ability barrier using the MAPPS framework. Indeed, a gap in full symptom recognition was evident. These findings align with recent evidence from the Horn of Africa showing that approximately half of caregivers’ delay seeking treatment despite awareness of malaria risks, often due to uncertainty about illness severity [25].

There was also evidence to suggest that delaying immediate access to care was a social norm within many communities. People take their cues to act from those around them and confidence in the experience of their immediate social group, albeit perhaps misplaced, was prevalent. This was coded as a social barrier to more timely access to care using the MAPPS framework. This again aligns with recent literature that states ideational factors such as perceived community norms and interpersonal communication are critical determinants of care-seeking; caregivers are significantly more likely to seek prompt care when they believe the behaviour is socially normative and endorsed by their community [26]. It demonstrates how knowledge alone is insufficient to drive prompt care-seeking and instead trust in the health system, interpersonal communication, and perceived community approval are critical determinants of whether caregivers seek care within 24 hours of fever onset [26]**.** Recommended strategies to address these challenges include focused educational initiatives to enhance comprehensive symptom recognition and a heightened awareness of the risks linked to delayed care. In our analysis, mapping these beliefs and norms to MAPPS motivation, ability and social dimensions help to differentiate behaviour patterns and guide distinct intervention types (e.g., risk communication versus norm-shifting)..

Physical barriers to timely access to care were also identified as limiting timely care-seeking behaviour. Distance to healthcare facilities, cost of transportation and competing responsibilities, such as work and childcare, made it difficult for individuals to seek care promptly. This was particularly acute in rural areas with limited healthcare infrastructure, as noted in previous research highlighting systemic challenges in sub-Saharan Africa. Some studies have observed that in rural communities, reliance on self-medication and traditional remedies is prevalent due to the scarcity of accessible healthcare facilities and diagnostic services [23,27]. Financial barriers, such as the cost of treatment and transportation, further discourage timely care-seeking behaviour [16]. Overcoming these physical barriers is more challenging as these often require systemic changes within whole healthcare systems to be made, for example increasing number of HCPs and facilities and universal funding of treatment. Recent research also demonstrates that distance to facilities, transport costs, perceived expensiveness of care, and fear of drug side effects act as key behavioural deterrents to timely treatment. Conversely, higher education and middle-income status serve as protective factors, highlighting how behavioural responses to affordability and access dictate treatment timelines [25].

### Inconsistent diagnostic testing

The second key barrier to timely access to care was inconsistent testing practices among healthcare professionals (HCPs) and patients. Over a third of adults and caregivers reported not being offered a diagnostic test to confirm their diagnosis when they last sought care for malaria. Additionally, 23% of HCPs stated a belief that their colleagues do not always conduct rapid diagnostic tests (RDTs) before prescribing treatment, and 35% stated that they believed patients do not expect to be tested. Only 27% saw it as their responsibility to ensure patients received a test.

HCPs recognised different barriers to testing that they experienced, for example not having the time to test or simply not having tests available. However, HCPs simultaneously reported the importance of testing and also their reliance on symptom presentation and clinical experience instead of diagnostic tests, demonstrating the possible existence of overconfidence bias. These were coded as physical and processing barriers in the MAPPS framework. Encouraging HCPs to adhere to testing protocols and implementing practical steps to facilitate this could help increase consistency in their approach. Training programmes that reinforce the importance, and potential negative consequences, of not seeking diagnostic confirmation could influence the way in which HCPs think about testing. Patients’ acceptance of non-testing practices also contributed to the lack of universal testing on symptom presentation. Patients reported concern for the possible cost of tests and therefore were willing to forgo testing in some cases. It is important to note that not all testing and treatments are charged for at healthcare facilities, but uncertainty as to possible charge can lead to the expectation of having to pay. Some patients reported not being offered a test and not actively asking for a test from the HCP. They were either not aware of the importance of testing or did not feel it was their place to do so given the professional status of the HCP. These barriers were coded as physical, ability and social using the MAPPS framework. Interventions to make the importance of testing salient amongst patients and interventions that target the social acceptability of patients’ proactive requests of the HCP may enable individuals to advocate for appropriate care and have the potential to overcome barriers to testing.. The broader literature supports that the behavioural acceptance of malaria rapid diagnostic tests (mRDTs) plays a critical role in treatment quality. While mRDTs can improve prescribing behaviour, their effectiveness ultimately depends on health worker adherence to the results and patient acceptance [28]. Furthermore, while not explicitly demonstrated in our study, in regions where pfhrp2/3 gene deletions or antigen persistence are prevalent, variable diagnostic performance can undermine trust in mRDTs among both providers and patients, leading to empiric treatment practices that bypass diagnostics entirely [29].

### Non-adherence to treatment

Non-adherence to ACTs can reduce treatment efficacy [30], and may contribute to the selection of artemisinin-resistant strains [31,32]. In our study, whilst 85% of adults and caregivers claimed to know the reasons for needing to take the full course of medication, over half reported prematurely discontinuing their medication, and 41% believed that treatment could be stopped once symptoms subsided. Previous research has found that adherence to antimalarial treatment regimens is influenced by several factors, including misconceptions about the necessity of completing medication once symptoms improve [23,33]. Furthermore, studies have shown caregivers commonly discontinue treatment prematurely due to misunderstandings of dosing schedules or the belief that the resolution of symptoms indicates full recovery, even though incomplete treatment contributes to the persistence of parasitaemia and potential development of drug resistance [27,34].

In this study we found that the expectation of a good outcome regardless of adherence was a motivational barrier to adherent behaviour. It was also reported that unpleasant taste and size of pills(We did not ask specifically about treatments given (e.g., when children complained about bitter-tasting pills) so miss specific data on this point.) can make medications difficult to ingest, particularly for children and adolescents, as seen in previous research [33]. This was coded as a physical barrier to adherence behaviour on behalf of the patients. Integrating medication schedules into daily lives was also reported to be challenging. Non-adherent behaviours were reportedly influenced by busy schedules and inconsistencies in routines, in addition to forgetfulness. The explicit classification of these behavioural influences within MAPPS made it clear that non-adherence in this context is not primarily a problem of information deficit, but of outcome expectations, routines and treatment design, pointing towards intervention options such as regimen simplification and routine support tools.

There was also evidence of a social dimension to adherence. Non-adherence was observed to be common within the community and 44% of respondents knew of people who had recovered despite not having completed their full course of treatment. Thus, non-adherence may be considered as an appropriate and socially acceptable approach. Simplifying treatment regimens and addressing physical barriers such as medication taste and pill size where possible could also enhance adherence. Implementing reminders and support tools, such as follow-up messages or community health worker visits, can address challenges in maintaining medication routines. HCPs also play a vital role in promoting adherence, as inadequate communication from healthcare providers regarding dosage instructions and the importance of completing the treatment course can contribute to confusion and non-adherence [35]. However, our study found that there was often a disconnection between what HCPs claim to communicate, and their confidence in their ability to communicate, and what patients understand or implement. Supporting HCPs to communicate the importance of adherence simply and clearly to patients, perhaps using visual materials, could address this issue. Within MAPPS, these findings map onto social and processing dimensions on the provider side as well, highlighting that strengthening adherence will require changes in both patient routines and provider communication practices.

Across the three behavioural domains examined (timely care seeking, diagnostic testing and adherence), the application of a behavioural framework provided a coherent way to integrate diverse qualitative narratives and survey findings and to link them systematically to intervention guidance. While many of the barriers identified, such as distance to care, cost or misconceptions about cure, have been reported previously, the MAPPS framework helped to clarify how these barriers operate across different cognitive and contextual dimensions and by making visible some less frequently articulated influences such as norms around delaying care or tacit assumptions of adherence by HCPs.

While this study focused primarily on the behavioural drivers of malaria treatment, it is important to acknowledge that prevention behaviours directly shape malaria incidence and subsequent treatment demand. Similar behavioural parallels are evident in prevention strategies. For example, despite widespread ownership of insecticide-treated nets (ITNs), adequate access remains inequitable across sub-Saharan Africa [36], and usage can paradoxically decline with increasing income and education in some rural settings [37]. However, exposure to targeted behavioural communication has been shown to successfully increase ITN use [38], reinforcing our conclusion that targeted behavioural communication is critical to reducing the overall malaria burden across both prevention and treatment paradigms.

### Country differences

It is also important to note country-level differences between respondents from the selected districts in Nigeria, Uganda, and Ivory Coast. Uganda demonstrated the most positive survey results, with significantly higher test-seeking behaviour among adults and caregivers, better understanding of antimalarial use, and higher likelihood of completing the full treatment course. HCPs were also more likely to offer RDTs and viewed testing as their responsibility. In contrast, in Nigeria, adults and caregivers showed significantly lower test-seeking behaviour, less trust in HCPs and their role in treating malaria and were less likely to view clinic visits as worthwhile. They also demonstrated lower awareness of the importance of testing and completing the full treatment course. Nigerian HCPs were also less likely to offer RDTs consistently and felt that it was more of a shared responsibility to test between patient and provider. Ivory Coast presented a unique profile, with significantly higher use of traditional medicine and greater belief in the efficacy of paracetamol compared to prescribed antimalarials. While HCPs in Ivory Coast were more likely to offer tests, patients showed significantly lower knowledge about the importance of completing the full antimalarial treatment course. While our research did not explore the reasons for differences across the three countries, these findings further highlight the need for locally tailored interventions to address specific behavioural barriers to effective malaria treatment.

### Limitations

There are some limitations to this study which should be considered when interpreting the data. Firstly, as with any self-reported data, respondents’ answers to questions may have been influenced by recall and/or social desirability bias. Secondly, this research was cross-sectional so only presents data from one period in time. Thirdly, the research employed non-probability sampling methods to meet sampling quotas; as a result, the findings cannot be considered generalisable to the populations in which the research was conducted. Additionally, we only conducted research in two districts per country, so the findings are also not nationally representative. To account for this limitation during our statistical analysis, we compared country-level data to the overall study mean rather than comparing countries directly to one another, as direct comparisons would imply a level of national representativeness that our sample does not possess.

Furthermore, the foundational literature review used to inform the study design and define key decision points was a rapid review rather than a systematic review. While this approach facilitated a timely synthesis of relevant behavioural literature, it did not employ the exhaustive search strategies and rigid inclusion/exclusion criteria characteristic of a systematic review, meaning some relevant prior research may not have been captured.

As previously mentioned in the discussion, we did not ask specific questions regarding the community health strategy (instead primarily focussing on questions regarding access at healthcare facilities and pharmacies), therefore there is a risk that insights regarding their role in access and adherence are missed. Furthermore, participants were not asked to identify the specific antimalarial treatments used in their responses, as the focus was on their general experiences and perceptions of malaria treatment. Despite these limitations, this study still provides comprehensive insights into behavioural barriers to malaria treatment, using the MAPPS framework to analyse the findings.

Because this study was funded by Novartis, strict measures were implemented to safeguard analytical and interpretive independence given the funder’s potential interest in antimalarial treatment outcomes. Data collection, thematic coding, and statistical analyses were conducted exclusively by independent researchers at Ipsos. Novartis-affiliated authors contributed to the study’s conceptual framing and manuscript review but did not participate in raw data collection or primary analysis, ensuring the findings objectively reflect the data gathered from participants.

## Conclusion

From a behavioural science perspective, our research findings suggest that a significant proportion of adults and caregivers delay seeking care due, in part, to difficulties in recognising malaria given non-specificity of symptoms and overconfidence in self-management. Inconsistent diagnostic testing practices, influenced by both healthcare providers’ clinical environment and patients’ lack of awareness or expectations regarding testing and treatment, further impede appropriate treatment. Additionally, many patients discontinue medication once symptoms subside, stemming from misconceptions about recovery and challenges with adherence. Addressing these behavioural barriers requires targeted interventions. Education and guidance designed to improve symptom recognition and emphasise the importance of completing treatment courses could enhance adherence. Training healthcare professionals to encourage consistent diagnostic testing and utilising social strategies to shift community perceptions should also be considered. By structuring our analysis around the MAPPS behavioural framework, we were able to locate these barriers within specific dimensions, thereby distinguishing barriers that can be addressed through targeted changes in practice or communication from those which are likely to require broader organisational or system-level adaptation. By applying the MAPPS behavioural framework, stakeholders can start to develop a range of targeted intervention strategies to improve access to care and adherence to antimalarial treatment. In this sense, MAPPS served not only as a descriptive tool but also as a pragmatic bridge between empirical finding and intervention design. Such interventions should be tested for their ability to relieve the malaria burden and advance effective malaria control in sub-Saharan Africa.

## Supporting information

S1 Text Qualitative interview guides. Full interview guides based on the three identified decision points.
(DOCX)

S2 Text Quantitative questionnaire. The structured questionnaire designed to assess behaviours and their influences at each stage.
(DOCX)

S1 Table Survey results by HCP type. Quantitative survey findings broken down by the type of healthcare professional.
(DOCX)

S2 Table Survey results by HCP type. Quantitative survey findings broken down by the type of healthcare professional.
(DOCX)

S3 Table Qualitative sample characteristics and quotas. Details of the participant breakdown, including the diverse mix of community respondents and healthcare professionals across locations and facility types.
(DOCX)

S4 Table Quantitative sample overview. Details of the final respondent breakdown for the 769 adult/caregivers and 532 healthcare providers who completed the survey.
(DOCX)

S5 Table Quantitative sample quotas. Details of the sample quotas set to ensure a mix of respondents for the quantitative phase.
(DOCX)
