# Supplementary material for: Understanding barriers to effective malaria diagnosis and treatment: A mixed-methods study in Uganda, Nigeria, and Ivory Coast
Source: PLOS Glob Public Health. 2026 Aug 17;6(8):e0006921. doi: 10.1371/journal.pgph.0006921 (PMC13480639; doi:10.1371/journal.pgph.0006921)
Supplement: Supplementary file 1 [file pgph.0006921.s001.docx]

**S1 Text: Interview guides**

**Behavioural Science Malaria Pathway Discussion Guide**

**Adults Without Care Responsibilities**

**60-minute in-depth interview**

Version 3

Internal / client use only

Healthcare Service Line, Ipsos UK

Ipsos MORI UK Ltd, 3 Thomas More Square, London, E1W 1WY, UK

Tel: +44 203 059 5000 Fax: +44 203 059 4998

Please note:

- **All instructions to the moderator are in bold**

**Materials for the interview:**

- **STIMULUS 1: RISK SCALES**
- **STIMULUS 2: SEVEREITY SCALES**
- **STIMULUS 3: DISEASE RANKING**

A. **Introduction (2 mins)**

**Moderator: Introduce yourself and Ipsos UK’s Healthcare Service Line.**

Thank you for agreeing to participate in this study. The purpose of this study is to understand your experiences with malaria. The interview will take approximately 60 minutes of your time.

**ADVERSE EVENTS**

If you mention any side effects or product complaints about the sponsoring company’s treatments, we will need to collect some additional details from you and pass them on to the sponsoring client. This is to help them learn more about the safety of their products. This will have no impact on your confidentiality during the interview itself.

Are you happy to participate with the interview on this basis?

Yes – **Continue**

No – **Thank you and close**

B. **Warm-up and introductions (10 mins) – 12 min total**

**Objective:** To build rapport and ascertain context for the respondent, by understanding their motivations and background. (Key MAPPS dimensions covered: Motivation, Physical, Social)

**Moderator say:** I'd like to start by asking you to briefly introduce yourself to me.

- What is your first name?
- How old are you?
- ***Motivation:*** What would you say are three most important things to you?
- ***Social:*** Who do you live with in your household?

***Ability:* Moderator say:** What does a typical day look like for you?

- What do you do?

- ***Physical:*** Where do you go? **Moderator to let respondent answer spontaneously without probing on specific places.**
  - How often do you go to these places generally?
  - How do you get around between these places? **Listen for and probe on transportation method e.g., walking, public transport, private transportation**
- ***Social:*** Who do you spend time with? **For questions below, listen out for and probe on family members, friends, community members.**
  - Who is the one person you spend the most time with?

**Moderator say:** I would like to now focus on health and how you feel about your health.

- ***Motivation, Processing:*** What do you think of when you think of someone who is ‘healthy’?
- ***Motivation:*** What, if anything, do you do to stay healthy?

**COMMUNITY LEADERS ONLY:**

- ***Motivation:*** You mentioned that you hold a community leadership role. Could you tell me more about it? What is the role specifically?
  - What sorts of things does your role entail – can you give some examples. **Probe: advising community members on different aspects of their lives**
- ***Social:*** What, if any, are your responsibilities related to the **health** of your community?
- ***Social:*** How often, if at all, do you talk to people in your community about their health and wellbeing?

C. **Framing of Malaria (10 mins) – 22 min total**

**Objective:** To understand attitudes and perceptions of malaria. (Key MAPPS dimensions covered: Motivation, Ability, Processing, Social)

**Moderator say: We are now going to focus the discussion specifically on Malaria.**

- ***Motivation:*** When I say malaria – what are the first things that come to mind?
- Pretend I do not know what malaria is. How would you describe it to me?
  - Where have you gained this knowledge of malaria?
    - **Probe on personal or family experience, community activities, media**
  - ***Social:*** Do you ever discuss malaria with your friends, family, community?
    - What sort of things do you talk about?

**Moderator show STIMULUS 1: RISK SCALES**

Here are a couple of different groups that could get malaria:

- ***Motivation:*** How likely is that these groups of people would get malaria on a scale of 1 to 5 with 1 being not at all likely and 5 being very likely?
  - Adults (18+ years)
  - Babies (under 5kg)
  - Infants (6 months – 6 years)
  - Children (7 years – 13 years)
  - Teenagers (13 years - 17 years)
  - **If answers differ for each question probe to understand why there are differences in risk / susceptibility**

**Moderator show STIMULUS 2: SEVERITY SCALES**

- ***Motivation:*** Let’s say, people from each of these groups end up getting malaria. How severe do you believe their sickness would be in each of the following cases? Please rate each group from 1 to 5, with 1 being not severe at all and 5 being very severe.
  - Adults (18+ years)
  - Babies (under 5kg)
  - Infants (6 months – 6 years)
  - Children (7 years – 13 years)
  - Teenagers (13 years - 17 years)
  - **If answers differ for each question probe to understand why there are differences in risk / susceptibility**
- ***Processing, Ability:*** How, if at all, do you protect yourself from malaria?
  - **Probe on bed nets, repellents, lifestyle, medication, self-medication**

**Moderator show STIMULUS 3: DISEASE RANKING**

***Motivation***, ***Processing***: **Moderator say:** Here you can see a list of diseases. Please rank these diseases according to which worries you the most to the least.

- Please think aloud as you make your choice / narrate what you are thinking
  - **Probe fully to understand positioning of malaria compared to other diseases**

**If not mentioned already, moderator say:** Just before we move on, what is your personal experience of malaria?

- When have you had malaria? How many times have you had malaria?
- ***Social:*** Do you know of others who have had malaria before?
  - What was their relationship to you?
  - What happened to them?

D. **Decisions involved in accessing malaria treatment (20 mins) – 42 min total**

**Objective:** To identify what drives behaviours when it comes to seeking care for malaria. (All MAPPS dimensions – but mainly focusing on Motivation, Physical, Social)

**DECISION 1: WHETHER TO SEEK CARE**

**Moderator say:** For the next set of questions, I will be asking you to think back to your (last or most recent) experience with malaria and what you did in certain situations.

- Firstly, I would like you to think back to the time when you started experiencing symptoms that made you feel unwell. What symptoms did you experience?
- ***Ability, Processing:*** Which symptoms made you think that you might have had malaria?
  - **Probe on headaches, tiredness, fever and chills**
- ***Motivation, Ability:*** Which of these symptoms, if any, made you start to worry?
  - **If any symptom(s) made them worry:** Why did these symptoms make you worry?
  - **For symptom(s) that did not make them worry:** Why was it that you weren’t worried about these symptoms?
  - What did you think was causing these symptoms?
- ***Motivation:*** Did you take any medication or use any treatments that you had at home?
  - **If yes:** Could you describe what you used i.e. how it looks, how to use/take it?
    - What was the medication meant to do? How did you know that it was working?
  - **If no:** Why not?
- ***Social:*** Did you ask anyone for advice?
  - **If yes:** Who did you ask for advice?
    - Why them?
    - What did they say to do?
    - **If not mentioned:** What about **[MODERATOR TO REFER BACK TO FRIENDS/FAMILY MENTIONED IN SECTION B]**?
  - **If no**: Why not?
  - Did you visit a traditional healer?
    - Why / why not?
- What do you think would’ve happened if you ignored those symptoms?
  - **Probe on headaches, tiredness, fever and chills**
  - Why do you think this?

**DECISION 2: WHERE TO SEEK CARE**

- ***MAPPS:*** Did you decide to see a medical professional about these symptoms?
  - **If they didn’t seek help at all:** Were there any reasons that stopped you from visiting a medical professional?
    - **Probe on ineffective medicines, counterfeit medicines, fear of stock-outs at clinic, lack of serious symptoms, cost, access, self-medication alternatives, distance, lack of awareness / education**
  - Was there anything that delayed your visit to a medical professional?
    - Why did this delay your visit?
  - **If they did seek help:** What type of medical professional did you see about these symptoms?
    - Why them?
    - ***Ability:*** How did you know who to see/where to go for help?
      - ***Social:*** Did you talk to anyone else about what to do?
    - Who else could you have gone and see if the medical professional you mentioned was not available? **Probe on pharmacist/nurse/community health worker/traditional healer if not mentioned**
      - Why?
        - **Probe on if certain types of healthcare professionals are easier to see than others**

**Moderator to be flexible depending on who respondent says is the first point of contact vs second point of contact:**

- ***MAPPS:*** What’s the difference between going to see [first point of contact] vs [second point of contact]?
  - ***Physical:*** How easy is it for you to see each one?
    - How long would you expect to wait until you see each one?
      - How do each of them they tend to you or serve you?
  - ***Motivation:*** Is there a difference in what you'd expect them to tell you?
- ***Physical:*** Where did you go to see this medical professional?
  - **Probe to understand whether they go to a public or private setting and whether it is a pharmacist, clinic or hospital**
  - Why did you go here?
    - ***MAPPS:*** What, if any, is the difference between what the public setting offers and what the private setting offers?
  - How did you get there?
    - How long did it take to get there?
    - Did you experience any issues with getting there?
      - **If so:** What were they?
- ***Physical:*** When you went to see that medical professional, how much did it cost you?
  - **Probe on travel fees, cost of appointment, cost associated with treatment**
  - Do you think it was worth the cost?
    - Why / why not?

**DECISION 3: ACCEPTING A RECOMMENDATION FOR TESTING/TREATMENT**

**If they did see a medical professional, moderator say:** So you went to discuss your condition with **[mention healthcare provider respondent mentioned in previous section].**

**If they didn’t see a medical professional, moderator say:** So from your last experience you didn’t see a medical professional.

**To all:** Let’s do some role play, where I would like you to pretend that I am a **[moderator** **to say healthcare professional that respondent considers a first point of contact]** that you, as a patient, could go to see about your symptoms.

(**If they did see a medical professional:** please draw from the interaction that you had with **[healthcare provider respondent mentioned in previous section]**)

- ***MAPPS:*** **Moderator to pretend they are a healthcare professional:** Hello ________, thank you for coming to see me today. Please explain your symptoms to me and why you are visiting me today. **Allow the conversation to unfold before moving on.**

**Moderator to say as healthcare professional:** So it seems you might have malaria. Before I give you any medicine I would first recommend that you take a diagnostic test to see whether or not you have malaria.

**Moderator to ask:**

- What is your initial reaction to being told you should test for malaria first?
- ***Motivation, Processing:*** What, if any, questions would you have about the test?
- ***Motivation, Processing:*** What, if any, value does testing have?
  - Why?

**Moderator say:** Okay. Now let’s say that you have tested positive for malaria.

- ***Motivation:*** What would you expect me as a medical professional to do next? **Probe provide medication, send me somewhere to get medication**
  - Do you usually receive a treatment on the same day?
  - Has there ever been a time where you were unable to get malaria treatment on the same day?
    - Why?
    - What happened next? How did you get your treatment?
- ***Motivation:*** What would the medication be like for malaria?
  - How are you suppose to take it? **Probe on amount, frequency and formulation**
  - How easy is it to take it?

**Moderator to say:** Ok thank you, we will stop the role play now and go back to questions about your own experience.

E. **Decisions involved in adhering to malaria treatment (10 mins – total 52 mins) for ADULTS who are not community leaders**

**SKIP SECTION FOR COMMUNITY LEADERS**

**Objective:** To identify key behaviours that lead to non-adherence to malaria treatment and what drives them. (All MAPPS dimensions – but mainly focusing on Motivation, Ability)

**SKIP THIS SECTION FOR COMMUNITY LEADERS**

**Moderator say:** I’d like you to think back again to the last time you had malaria.

- ***Ability:*** How did you know what to do with the treatment you have been given?
- ***Ability, Social:*** What did the HCP tell you - how do they explain what to do? like for malaria?
  - How are you supposed to take it? **Probe on amount, frequency and formulation**
  - How did you take them?
  - ***MAPPS:*** Did you experience any challenges when taking the medication?
  - Were you able to take the medication every day? At the same time every day?
    - **If yes:** What helped you remember?
    - **If no:** Why not? **(Probe: difficulty/barriers).** What would have helped you remember?
  - Was there anything that you used or did to take care of yourself in addition to this treatment?
    - **Probe on self-medication, alternative medicines**
    - Did you do/use this at the same time?
    - What impact did it have on your symptoms?
- ***Motivation:*** How did you know if the medication was working?
  - What signs would suggest that it was working?
  - What signs would suggest that it was not working?
- On a scale of 1 to 5 where 1 is not at all satisfied and 5 is very satisfied, how satisfied are you with the treatments available for malaria?
  - Why?
- ***MAPPS:*** From our research, we know that sometimes patients with malaria do not always finish all of the medicine that their **[mention healthcare provider respondent mentioned in previous section]** gives them. Why do you think this is?
  - Have you ever done this?
    - Why / why not?
  - Did your **[mention healthcare provider respondent mentioned in previous section]** say anything about this when giving you the medication?
    - What did they say?

F. **COMMUNITY LEADERS ONLY: Community leader experience (10 mins – total 52mins) for ADULTS who are community leaders**

**Objective:** To understand the community leader’s role in community member’s healthcare decisions and involvement in malaria care. (MAPPS Dimensions: Motivation, Social)

**SKIP THIS SECTION FOR ADULTS WHO ARE NOT COMMUNITY LEADERS**

**Moderator say:** Before we finish the interview I would like to learn a bit more about your role as a community leader. Earlier you mentioned you are **[x].**

- ***Social:*** Who, if anyone, seeks your guidance / support?
  - Why do they think you can help and support them?
  - **IF NOT MENTIONED:** Do adults consult you regarding their own challenges?
  - **IF NOT MENTIONED:** Do mothers of young children also consult you?
- ***Motivation, Social:*** What help and support do you offer?
  - How often do people follow your advice / recommendations?
  - Where does the advice you share with your community come from?
    - **Probe on generational wisdom, whether they have a healthcare care background**
- ***Social:*** Who are the people in your community who play a role in helping people to be healthy?
  - **Probe if needed: family, neighbours, doctors, nurses, traditional healers, religious leaders**
  - What are the things they offer to help people be healthy?
  - How often, if at all, do you work with these people to keep the community healthy? How are their roles similar to yours? How are their roles different?
- ***Social:*** Have any adults in your community ever approached you for advice on malaria for themselves?
  - **If so:** What advice did you give them and why?
  - **If so:** To what extent did they follow this advice?
  - **If so:** How did this impact their experience with malaria?
- ***Social:*** Have any adults in your community ever approached you for advice on malaria for their children?
  - **If so:** What advice did you give them and why?
  - **If so:** To what extent did they follow this advice?
  - **If so:** How did this impact their experience with malaria?
- Can you think of any opportunities for you to talk to people about seeking help from a healthcare professional for a malaria diagnosis?
  - Where/ when would this be?
  - What would you say to them?
  - Are there any reasons/ circumstances why it would not be appropriate for you to talk to people about malaria?

**G.	Close (3 mins – total 60 mins)**

**Objective:** To understand what messages around malaria are currently being socialised within the community and what is driving this. (All MAPPS Dimensions – understanding existing interventions in place)

**Moderator say:** Before we finish the interview, I would like to ask you some final questions about what is happening in your community when it comes to malaria.

- ***MAPPS:*** Have you seen any campaigns or adverts about malaria in your community?
  - Can you remember what they said?
  - Who were the messages from?
- ***MAPPS:*** Which of the diseases we mentioned earlier do you hear about the most? **Moderator refer back to ranking from STIMULUS 3: DISEASE RANKING**

**H.	At the end of the interview**

**Text if no Adverse Event/Product Complaint identified**

- Thank and close

**Text if respondent raises an Adverse Event/Product Complaint**

**INTERVIEWER SAY:**

Thanks for participating in and completing our market research project.

You mentioned an adverse event or product complaint during the interview which must be reported to the sponsoring company. The sponsoring company of this market research project has an obligation to continuously monitor the safety of their products. Although rare, their Product Safety Department may want to speak with you to further investigate those issues you may have experienced.

Are you willing to assist with the reporting of this adverse event/product complaint by providing further information to the sponsoring company?

- Yes, I am willing to provide more information regarding the adverse event/product complaint – **[CONTINUE READING]**
- No, I am not willing to provide more information regarding the adverse event/product compliant. – **[CONTINUE READING]**

**IF NO; INTERVIEWER SAY:** Thank you. I will file this report without giving any of your contact details. **[FINISH INTERVIEW]**

**IF YES; INTERVIEWER SAY:** Would you be willing to have your personal data passed on to the sponsoring company so they can contact you directly about any queries? If so, you have the right to know who the sponsoring company is, we will reveal the name to you.

☐ Yes, I agree **[RECORD RESPONDENT’S DETAILS WITHIN THE PV REPORT]**

☐ No, I do not agree**. [RECORD AS NO ON AER FORM, END OF SURVEY]**

**IF YES;** Please note that you have consented to have your personal data passed on to the sponsoring company, **[INSERT CLIENT NAME],** such data will be controlled and processed by their Product Safety Department as outlined in their privacy notice that can be reviewed at this link **[PROVIDE LINK TO CLIENT PRIVACY NOTICE/PROVIDE COPY OF PRIVACY NOTICE].**

**AT END OF INTERVIEW**

**[QCLIENT IF APPLICABLE, READ]:** As mentioned at the beginning of the survey we can now reveal that this market research project is sponsored by Novartis. You can find more information on how the sponsoring company processes your data on their privacy notice.

**Behavioural Science Malaria Pathway Discussion Guide**

**Caregivers**

**60-minute in-depth interview**

Version 3

Internal / client use only

Healthcare Service Line, Ipsos UK

Ipsos MORI UK Ltd, 3 Thomas More Square, London, E1W 1WY, UK

Tel: +44 203 059 5000 Fax: +44 203 059 4998

Please note:

- **All instructions to the moderator are in bold**

**Materials for the interview:**

- **STIMULUS 1: RISK SCALES**
- **STIMULUS 2: SEVEREITY SCALES**
- **STIMULUS 3: DISEASE RANKING**

**A.	Introduction (2 mins)**

**Moderator: Introduce yourself and Ipsos UK’s Healthcare Service Line.**

Thank you for agreeing to participate in this study. The purpose of this study is to understand your experiences with malaria. The interview will take approximately 60 minutes of your time.

**ADVERSE EVENTS**

If you mention any side effects or product complaints about the sponsoring company’s treatments, we will need to collect some additional details from you and pass them on to the sponsoring client. This is to help them learn more about the safety of their products. This will have no impact on your confidentiality during the interview itself.

Are you happy to participate with the interview on this basis?

Yes – **Continue**

No – **Thank you and close**

**B.	Warm-up and introductions (10 mins – total 12 mins)**

**Objective:** To build rapport and ascertain context for the respondent, by understanding their motivations and background. (Key MAPPS dimensions covered: Motivation, Physical, Social)

**Moderator say:** I'd like to start by asking you to briefly introduce yourself to me.

- What is your first name?
- How old are you?
- ***Motivation:*** What would you say are three most important things to you?
- ***Social:*** Who do you live with in your household?

***Ability:* Moderator say:** What does a typical day look like for you?

- What do you do?
- ***Physical:*** Where do you go?
  - How often do you go to these places generally?
  - How do you get around between these places? **Listen for and probe on transportation method e.g., walking, public transport, private transportation**
- ***Social:*** Who do you spend time with? **For questions below, listen out for and probe on family members, friends, community members.**
  - Who is the one person you spend the most time with?
- Do you have children or look after someone else’s children regularly?
  - How many?
  - Please describe each one to me including
    - How old they are
    - Their gender
    - If you are their main caregiver? If not, who is?
  - How would you describe your responsibilities as a caregiver for them?
    - Where do you care for them?

**Note for moderator: Throughout moderator will need to adapt style of questioning to suit whether respondents have their own child or care for a child that is not theirs.**

**COMMUNITY LEADERS ONLY:**

- ***Motivation:*** You mentioned that you hold a community leadership role. Could you tell me more about it? What is the role specifically?
  - What sorts of things does your role entail – can you give some examples. **Probe: advising community members on different aspects of their lives**
- ***Social:*** What, if any, are your responsibilities in the **healthcare** of your community?
  - Are you involved in any decisions about how community funds are spent on healthcare?
    - **If so:** Please describe how you are involved in these decisions.
- ***Social:*** How often, if at all, do you talk to people in your community about their health and wellbeing?

**C.	Framing of Malaria (10 mins – total 22 mins)**

**Objective:** To understand attitudes and perceptions of malaria. (Key MAPPS dimensions covered: Motivation, Ability, Processing, Social)

**Moderator say:** I would now like to focus on your experiences of malaria.

- ***Motivation:*** When I say malaria – what are the first things that come to mind?
- Pretend I do not know what malaria is. How would you describe it to me?
  - Where have you gained this knowledge of malaria?
    - **Probe on personal or family experience, community activities, media**
  - ***Social:*** Do you ever discuss malaria with your friends, family, community
    - What sort of things do you talk about?
    - Do the sort of things you discuss change when talking about infants/ children with malaria rather than adults?
      - Why / why not?
- What, if anything, do you know about malaria in infants (6 months – 6 years)?
  - How, if at all, does it differ to malaria in children (7-13 years)?
  - How, if at all, does it compare to malaria in adults?

**STIMULUS 1: RISK SCALES**

Here are a couple of different groups that could get malaria:

- ***Motivation:*** How likely do think it would be that people in this group would get malaria on a scale of 1 to 5, with 1 being not at all likely and 5 being very likely?
  - Adults (18+ years)
  - Babies (under 5kg)
  - Infants (6 months – 6 years)
  - Children (7 years – 13 years)
  - Teenagers (13 years - 17 years)
  - **If answers differ for each question probe to understand why there are differences in risk / susceptibility**

**STIMULUS 2: SEVERITY SCALES**

- ***Motivation:*** Let’s say, all of them end up getting malaria. How severe do you believe the illness would be in each of the following cases? Please rate each group from 1 to 5, with 1 being not severe at all and 5 being very severe.
  - Adults (18+ years)
  - Babies (under 5kg)
  - Infants (6 months – 6 years)
  - Children (7 years – 13 years)
  - Teenagers (13 years - 17 years)
  - **If answers differ for each question probe to understand why there are differences in risk / susceptibility**
- ***Processing, Ability:*** How, if at all, do you protect yourself from malaria?
  - **Probe on bed nets, repellents, lifestyle, medication, self-medication**
- ***Processing, Ability:*** How, if at all, do you protect your child/children you look after from malaria?
  - Does this differ depending on the age of the child you are caring for?
    - **Probe:** Why / why not?
  - **Probe on bed nets, repellents, lifestyle, medication, self-medication**
  - **Probe on differences between protection methods for adults vs. children, or children of different ages**

**STIMULUS 3: DISEASE RANKING**

**Moderator say:** Here you can see a list of diseases. Please rank these diseases according to which worries you the most to the least

- Please think aloud as you make your choice / narrate what you are thinking
  - **Probe fully to understand positioning of malaria compared to other diseases**

**If not mentioned already, moderator say:** Just before we move on, what is your personal experience of malaria?

- When have you had malaria? How many times have you had malaria in your life?
- ***Social:*** Do you know of others who have had malaria before?
  - What was their relationship to you?
    - **If not mentioned: probe on their children/children that they looked after**
  - What happened to them?

**D.	Decisions involved in accessing malaria treatment for children (15 mins – total 37 mins)
(COMMUNITY LEADERS: 15 mins – 37 mins)**

**Objective:** To identify what drives differing behaviours when it comes to seeking care for malaria. (All MAPPS dimensions – but mainly focusing on Motivation, Physical, Social)

**DECISION 1: WHETHER TO SEEK CARE**

**Moderator say:** For the next set of questions, I will be asking you think back to the experience of when your child/one of the children you care for had malaria, and what you did in certain situations.

- Firstly, I would like you to think back to the time that your child/the child you look after experienced symptoms that make them feel unwell. What symptoms did they have?
- ***Ability, Processing:*** Which symptoms made you think that they might have malaria?
  - **Probe on headaches, tiredness, difficulty or refusing feeding, fever and chills**
  - **If talking about a baby:** Were there any particularly unusual symptoms?
    - **If so:** What?
- ***Motivation, Ability:*** Which of these symptoms made you start to worry?
  - Why did these symptoms make you start to worry?
  - What did you think was causing these symptoms?
- ***Processing:*** What do you think would happen if you ignored their symptoms?
  - **Probe on headaches, tiredness, difficulty feeding, fever and chills**
  - Why do you think this?
- ***Motivation:*** Did you give them any medications or treatments that you had at home?
  - **If yes:** Could you describe what you gave to the child i.e. how it looks, how to use/take it?
    - What was the medication meant to do? How did you know that it was working?
  - **If no:** why not?
- ***Social:*** Did you ask anybody for advice on what to do?
  - **If yes:** Who did you ask for advice?
    - Why them?
    - What did they say to do?
    - **If not mentioned:** What about **[MODERATOR TO REFER BACK TO FRIENDS/FAMILY/COMMUNITY HEALER/ RELIGIOUS LEADER MENTIONED IN SECTION B]**?
  - **If no**: Why not?
  - Did you visit a traditional healer?
    - Why / why not?

**Moderator say:** Now I would like you to imagine it was you who was experiencing these same symptoms.

- ***Ability:*** Which of these symptoms, if any, do you think you would experience if you had malaria?
  - Why?
- ***Motivation:*** Which, if any, of these symptoms make you worry?
- ***Social, Physical:*** At what point would you seek some help for yourself – what would your symptoms need to be like? What would you do first?
- How does this compare to what you said you would do if it was your child/the child you look after experiencing these symptoms?
  - **Probe on any differences**
  - Would you do anything differently depending on if the child was a girl/boy?

**DECISION 2: WHERE TO SEEK CARE**

**Moderator say:** Let’s focus on your child/the child you look after again.

- ***MAPPS:*** At what point did you see a medical professional about these symptoms?
  - **If they didn’t seek help at all:** Were there any reasons that stopped you from visiting a medical professional?
    - **Probe on perceived ineffective medicines, counterfeit medicines, lack of serious symptoms, stock-outs, cost, access, self-medication alternatives, distance, lack of trust in healthcare provider/system**
  - Was there anything that delayed your visit to a medical professional?
    - Why did this delay your visit?
  - **If they did seek help:** What type of medical professional did you see about these symptoms?
    - Why them?
    - ***Ability:*** How did you know who to see/where to go for help?
      - ***Social:*** Did you talk to anyone else about what to do?
    - Who else could you have gone and see if the medical professional you mentioned was not available? **Probe on pharmacist/nurse/community health worker/traditional healer if not mentioned**
      - Why?
        - **Probe on if certain types of healthcare professionals are easier to see than others**

**Moderator to be flexible depending on who respondent says is the first point of contact vs second point of contact:**

- ***MAPPS:*** What’s the difference between going to see [first point of contact] vs [second point of contact]?
  - ***Physical:*** How easy is it for you to see each one?
    - How long would you expect to wait until you see each one?
      - How do each of them they tend to you or serve you?
  - ***Motivation:*** Is there a difference in what you'd expect them to tell you?
- ***Physical:*** Where did you go to see this medical professional?
  - **Probe to understand whether they go to a public or private setting and whether it is a clinic or hospital**
  - Why did you go here?
    - ***MAPPS:*** What, if any, is the difference between what the public setting offers and what the private setting offers?
  - How did you get there?
    - How long did it take to get there?
    - Did you experience any issues with getting there?
      - **If so:** What were they?

**Moderator to amend question depending on age of child/children of respondent to ensure all ages are covered by questioning.**

- ***MAPPS:*** What would you do if it was a baby experiencing these symptoms, what would you tell their caregiver to do? What should they do differently to what you mentioned?
  - Why?
- ***MAPPS:*** What about if a teenager was experiencing these symptoms, what would you tell their caregiver to do? What should they do differently to what you mentioned?
  - Why?
  - **If responses are different for each age group probe to understand rationale behind differences**
- Would you do anything differently depending on if the child was a girl/boy?
  - Why / why not?
- ***MAPPS:*** What about if it was you experiencing these symptoms, what would you do differently to what you did with your child/the child you care for?
  - Why?
  - **If responses are different, probe to understand rationale behind differences**

**DECISION 3: ACCEPTING A RECOMMENDATION FOR TESTING/TREATMENT**

**If they did take the child to see a medical professional, moderator say:** So you took your child/the child you care for to see **[mention healthcare provider respondent mentioned in previous section].**

**If they didn’t take the child to see a medical professional, moderator say:** So from your last experience you didn’t take your child/the child you care for to a medical professional.

**To all:** Let’s do some role play, where I would like you to pretend that I am a medical professional that you took your child/the child you care for to see me about their symptoms.

(**If they did take the child to see a medical professional:** please draw from the interaction that you had with **[healthcare provider respondent mentioned in previous section]**)

- ***MAPPS:*** **Moderator to act as healthcare professional:** Thank you for coming to see me today. Please explain the child’s symptoms to me and why you are visiting me. **Allow the conversation to unfold before moving on.**

**Moderator say:** Now imagine that I tell you, it seems that the child might have malaria, but before I prescribe any treatment first the child must take a diagnostic test to see whether or not they have malaria.

- What is your initial reaction?
- ***Motivation, Processing:*** What, if any, questions would you have about the test?
- ***Motivation , Processing:*** What do you think is the importance of testing, if any?
  - Why?
- How much do you think a test would cost?
- Would you be willing to pay for a test for a baby or infant?
  - Why?
- Would you be willing to pay for a test for a child?
  - Why?
- Would you be willing to pay for a test for an adult?
  - Why?
- If your child was not offered a test, would you ask for one?
  - Why?
- Have you ever been offered a test for your child and refused it?
  - Why did you refuse the test?

**Moderator say:** Okay. Let’s say that the child has tested positive for malaria.

- What would you expect me as a medical professional to do next? **Probe provide medication, send me somewhere to get medication**
  - Do you usually receive a treatment on the same day?
  - Has there ever been a time where you were unable to get malaria treatment on the same day?
    - Why?
    - What happened next? How did you get your treatment?
- ***Motivation:*** What would the medication be like for malaria?
  - How are you suppose to take it? **Probe on amount, frequency and formulation**
  - How easy is it to take it?
    - Do you think it would be easier or more difficult if a baby or infant had to take this treatment compared to a child?
      - Why?
- ***MAPPS:*** If a friend/family member had a child (with different age to the participant’s child/the child they care for) suffering from these symptoms, what advice would you give them? What would you tell them to be mindful or aware of when seeing a medical professional?
  - Why?
  - **Probe on any differences between advice here and answers to previous questions**
- ***MAPPS:*** If an adult friend/family member **[moderator to refer to friend/family member mentioned in the introduction]** was themselves suffering from these symptoms, what advice would you give them? What would you tell them to be mindful or aware of when seeing a medical professional?
  - Why?
  - **Probe on any differences between advice here and answers to previous questions**

**Moderator to say:** Thank you, now we’ll stop the role play and I am going to ask you some questions about your real-life experience again.

**E.	Decisions involved in adhering to malaria treatment (10 mins – total 47 mins)**

**Objective:** To identify key behaviours that lead to non-adherence to malaria treatment and what drives them. (All MAPPS dimensions – but mainly focusing on Motivation, Ability)

**SKIP SECTION FOR COMMUNITY LEADERS**

- Now thinking back to when your child/the child you look after had malaria, what was the medication you were given?
- ***Ability:*** How did you know what to do with the treatment you have been given for your child/the child you care for?
  - ***Ability***, ***Social***: What did the HCP tell you - how do they explain what to do?
  - Would the instructions be similar for adults as well? **Probe on difference in frequency and formulation in adults vs children**
  - How did you give the treatment to the child/how did they take them?
  - ***MAPPS:*** Did the child experience any challenges when taking the medication?
  - Was the child able to take the medication every day? At the same time every day?
    - **If yes:** What, if anything, helped you remember to give it to them?
    - **If no:** Why not? **(Probe: difficulty/barriers).** What would have helped you remember?
    - **If no:** What, if anything, did you do when they didn’t take their medication?
  - Was there anything that you gave the child or did to take care of the child in addition to this treatment?
    - **Probe on self-medication, alternative medicines**
    - Did you do/use this at the same time?
    - What impact did it have on their symptoms?
  - Was this experience the same as when you have had to take medication in the past? Why or why not?
  - Was your child breastfeed?
    - What, if anything, are the benefits of breastfeeding? **Moderator to allow respondent to list as many benefits as possible**
      - **If not mentioned:** Have you ever heard about the benefits of breastfeeding and child immunity to disease?
        - **If so:** What have you heard?
- ***Motivation:*** How did you know if the medication is working?
  - What signs suggest that it is working?
  - What signs suggest that it is not working?
  - Was this the same if you were taking the medication yourself, from your own experience with malaria? Are these the signs you look for yourself?
- ***MAPPS:*** From our research, we know that sometimes adults looking after children with malaria do not always finish all of the medicine that their **[mention medical professional respondent mentioned in previous section]** gives them. Why do you think this is?
  - Have you ever done this?
    - Why / why not?
  - Did your **[mention medical professional respondent mentioned in previous section]** say anything about this when giving you the medication?
    - What did they say?
  - What about when receiving malaria medication yourself? Have you ever not finished all of your medication?
    - Why / why not?
    - **Probe for any differences between adult and child**

**F.	COMMUNITY LEADERS ONLY: Community leader experience (10 mins – total 57 mins)**

**Objective:** To understand the community leader’s role in community member’s healthcare decisions and involvement in malaria care. (MAPPS Dimensions: Motivation, Social)

**SKIP SECTION FOR CAREGIVERS WHO ARE NOT COMMUNITY LEADERS**

**Moderator say:** Before we finish the interview I would like to learn a bit more about your role as a community leader. Earlier you mentioned you are **[x].**

- ***Motivation:*** How would people describe what you do in the community?
  - What is expected of you?
  - What do you enjoy about your role in the community?
  - And what do you not enjoy about it?
  - What are things you want to achieve for the community?
  - Who seeks your guidance / support?
    - Why do they think you can help and support them?
    - **IF NOT MENTIONED:** Do adults consult you regarding their own challenges?
    - **IF NOT MENTIONED:** Do mothers of young children also consult you?
  - What help and support do you offer?
    - How often do people follow your advice / recommendations?
- ***Social:*** Who are the people in your community who play a role in helping people to be healthy?
  - **Probe if needed: family, neighbours, doctors, nurses, traditional healers, religious leaders**
  - What are the things they offer to help people be healthy?
  - To what extent, if at all, do you work with these people in the community to keep the community healthy? How are their roles similar to yours? How are their roles different?
- ***Social:*** Have any adults in your community ever approached you for advice on malaria for themselves?
  - **If so:** What advice did you give them and why?
  - **If so:** To what extent did they follow this advice?
  - **If so:** How did this impact their experience with malaria?
- ***Social:*** Have any adults in your community ever approached you for advice on malaria for their children?
  - **If so:** What advice did you give them and why?
  - **If so:** To what extent did they follow this advice?
  - **If so:** How did this impact their experience with malaria?
- Can you think of any opportunities for you to talk to people about seeking help from a medical professional for a malaria diagnosis?
  - Where/ when would this be?
  - What would you say to them?
  - Are there any reasons/ circumstances why it would not be appropriate for you to talk to people about malaria?

**G.	Close (3 mins – total 60 mins)**

**Objective:** To understand what messages around malaria are currently being socialised within the community and what is driving this. (All MAPPS Dimensions – understanding existing interventions in place)

**Moderator say:** Before we finish the interview, I would like to ask you some final questions about what is happening in your community when it comes to malaria.

- ***MAPPS:*** Have you seen any campaigns or adverts about malaria in your community?
  - **If so:** What did they say?
  - **If so:** Where did you see these campaigns?
    - **Probe on pharmacy, public health facilities, community health worker activities**
    - What, if anything, did these teach you?
  - **If not:** Why do you think this is?
- ***MAPPS:*** **If not mentioned:** Have you heard anything about babies with malaria in particular?
  - **Probe on babies already having protection against malaria**
  - **Probe on babies requiring bed nets**
- ***MAPPS:*** Which of the diseases we mentioned earlier do you hear about the most? **Moderator refer back to ranking from STIMULUS 3: DISEASE RANKING**

**H.	At the end of the interview**

**Text if no Adverse Event/Product Complaint identified**

- Thank and close

**Text if respondent raises an Adverse Event/Product Complaint**

**INTERVIEWER SAY:**

Thanks for participating in and completing our market research project.

You mentioned an adverse event or product complaint during the interview which must be reported to the sponsoring company. The sponsoring company of this market research project has an obligation to continuously monitor the safety of their products. Although rare, their Product Safety Department may want to speak with you to further investigate those issues you may have experienced.

Are you willing to assist with the reporting of this adverse event/product complaint by providing further information to the sponsoring company?

- Yes, I am willing to provide more information regarding the adverse event/product complaint – **[CONTINUE READING]**
- No, I am not willing to provide more information regarding the adverse event/product compliant. – **[CONTINUE READING]**

**IF NO; INTERVIEWER SAY:** Thank you. I will file this report without giving any of your contact details. **[FINISH INTERVIEW]**

**IF YES; INTERVIEWER SAY:** Would you be willing to have your personal data passed on to the sponsoring company so they can contact you directly about any queries? If so, you have the right to know who the sponsoring company is, we will reveal the name to you.

☐ Yes, I agree **[RECORD RESPONDENT’S DETAILS WITHIN THE PV REPORT]**

☐ No, I do not agree**. [RECORD AS NO ON AER FORM, END OF SURVEY]**

**IF YES;** Please note that you have consented to have your personal data passed on to the sponsoring company, **[INSERT CLIENT NAME],** such data will be controlled and processed by their Product Safety Department as outlined in their privacy notice that can be reviewed at this link **[PROVIDE LINK TO CLIENT PRIVACY NOTICE/PROVIDE COPY OF PRIVACY NOTICE].**

**AT END OF INTERVIEW**

**[QCLIENT IF APPLICABLE, READ]:** As mentioned at the beginning of the survey we can now reveal that this market research project is sponsored by Novartis. You can find more information on how the sponsoring company processes your data on their privacy notice.

**Behavioural Science Malaria Pathway Discussion Guide
Healthcare Professional:**

**60 minutes**

Version 3

Internal / client use only

Ipsos Healthcare

3 Thomas More Square, London, E1W 1YW, UK

Tel: +44 203 059 5000 Fax: +44 203 059 4998

**Research objectives:**

- Identify attitudes, beliefs and behaviours relating to malaria
- Explore the diagnosis and treatment experiences of adult and childhood malaria from the perspective of healthcare professionals who treat malaria
- Understand the role the different types of healthcare professional play in diagnosis, treatment and patient adherence to treatment
- Understand what drives their decision making relating to diagnosis, treatment and management whilst on treatment
- Identify the barriers to optimum HCP diagnosis and treatment behaviour and the mechanisms underpinning those barriers

Please note:

- **All instructions to the moderator are in bold**

**Materials for the interview:**

- **STIMULUS 1: PATIENTS DIAGRAM**
- **STIMULUS 2: SEVERITY RANKING**
- **STIMULUS 3: RISK SCALES**
- **STIMULUS 4: SEVERITY SCALES**
- **STIMULUS 5: TREATMENTS BUBBLE DIAGRAM**

**A.	Introduction (2 mins)**

**Moderator: Introduce yourself and Ipsos Healthcare**

Thank you for agreeing to participate in this research. The reason why we’re talking to healthcare professionals like yourself is to understand how malaria in adults and children is managed. We’ll be talking for around 60 minutes.

Before we start, I would like to discuss adverse events. Different patients sometimes respond in different ways to the same medicine, and some side effects/ problems may not be discovered until many people have used that medicine over a period of time. For this reason, we are required to pass on to our client who is sponsoring the research and is a manufacturer of medicines, details of any side effects/problems related to their own product(s) that are mentioned during this research, within one business day of their occurrence. Although what you say will, of course, be treated in confidence, if you say that your patient experienced a side effect or became ill after taking one of our client’s medicines, we will need to report this, so that they can learn more about the safety of their medicines.

Are you happy to participate with the interview on this basis?

Yes – **Continue**

No – **Thank you and close**

**B: Introduction and background (8 minutes – total 10 mins)**

**Objective:** To build rapport and ascertain context for the HCP, by understanding their roles and responsibilities and patients (Key MAPPS Dimensions: Motivation, Physical, Social)

**Moderator say:** I'd like to start by asking you to briefly introduce yourself to me.

- What is your first name?
- ***Physical:*** Tell me about the place that you work in – what is it like?
  - **Probe on location, rural/urban, hospital/clinic type, private/public**
- ***Motivation:*** What are your specific roles and responsibilities there?
  - ***Ability:*** How long have you been doing this role for?
    - **ONLY FOR PHARMACISTS:** Are you also the business owner?
  - ***Social:*** Who else do you work with? What is their role?
- ***Motivation:*** What is your favourite thing about being a **[insert healthcare provider role]**?
  - What is your main goal as a **[insert healthcare provider role]**?
- ***Motivation:*** What are the most challenging things about being a **[insert healthcare provider role]**?
  - What makes this so challenging?
    - **Ask for a specific example of challenge**
- How would you describe the patients you see?
  - **Probe on gender, socioeconomic status, age**

**Moderator to show STIMULUS 1: PATIENTS DIAGRAM**

**Moderator say and complete worksheet as the respondent is talking:** Now I would like us to complete this diagram.

- What proportion of your patients are each of the following?
  - Adults (18+ years)
  - Babies (under 5kg)
  - Infants (6 months – 6 years)
  - Children (7 years – 13 years)
  - Teenagers (13 years - 17 years)
- ***Motivation***, ***Processing***: What are the conditions that you tend to see / have to deal with most frequently?
  - Do the types of conditions that you treat differ by each of these groups?
    - **If so:** How?

**SECTION 2 – Framing of Malaria (10 minutes)**

**Objective:** To understand attitudes, perceptions and experience with managing malaria. (Key MAPPS Dimensions: Motivation, Ability, Processing)

**Moderator say:** I would now like to focus on malaria.

- ***Motivation:*** What comes to mind when someone says the word ‘malaria’ to you?
- ***Ability:*** Pretend I do not know what malaria is. How would you describe it to me?
  - Where did you learn that?
    - **Probe for: personal experience vs different kinds of information sources**

**Moderator show STIMULUS 2: SEVERITY RANKING**

Here you can see a list of diseases [see workbook]. Please rank these diseases according to which worries you the most to the least.

- Please think aloud as you make your choice / narrate what you are thinking
  - **Probe fully to understand positioning of malaria compared to other diseases**

So here are some different groups that can be affected by malaria:

**Moderator show STIMULUS 3: RISK SCALES**

- ***Motivation:*** How likely is that these groups of people would get malaria on a scale of 1 to 5, with 1 being not at all likely and 5 being very likely?
  - Adults (18+ years)
  - Babies (under 5kg)
  - Infants (6 months – 6 years)
  - Children (7 years – 13 years)
  - Teenagers (13 years - 17 years)
  - **If answers differ for each question probe to understand why there are differences in risk / susceptibility**

**Moderator show STIMULUS 4: SEVERITY SCALES**

- ***Motivation:*** Let’s say, people from each of these groups end up getting malaria. How bad do you believe their sickness would be in each of the following cases? Please rate each group from 1 to 5, with 1 being not severe at all and 5 being very severe.
  - Adults (18+ years)
  - Babies (under 5kg)
  - Infants (6 months – 6 years)
  - Children (7 years – 13 years)
  - Teenagers (13 years - 17 years)
  - **If answers differ for each question probe to understand why there are differences in risk / severity**

**Moderator say:** Let’s talk about your specific experience of Malaria.

- What proportion of patients that you have seen in the last malaria season had malaria? Is this normal or specific to the time of year?
  - What proportion of those patients with malaria were adults?
  - What proportion were children or teenagers (7 – 18 years)
  - What proportion were infants (6 months – 6 years)?
  - And what proportion were babies weighing less than 5kg?
- ***Motivation:*** What is your role when it comes to seeing patients with malaria?
  - ***If not mentioned probe on role in* diagnosis, testing and treatment**
  - Does your role change depending on the age of the patients you see?
    - **If so:** How?
- ***Motivation***, ***Processing***: How, if at all, does managing malaria compare to the other disease / conditions you diagnose and treat?
  - **Probe on comparisons with diseases mentioned earlier**
    - **If not answered:** How does it compare in terms of severity compared to other diseases?
  - How do you feel treating different types of patients with malaria? Are some harder to care for than others?
    - Which ones?
      - Why?
- ***Motivation:*** Have you ever had malaria yourself?
  - **If yes:** When did you have it?
  - **If yes:** What did you do?
    - Why did you do this?

**SECTION 3 – Drivers of HCP behaviour across the adult pathway (20 minutes)**

**Objective:** To understand what drives HCP behaviours relating to diagnosis, treatment and encouragement of adherence for adult patients (All MAPPS dimensions)

***MAPPS:*** **Moderator say:** For the next set of questions, I will be asking you to tell me the story of the last interaction you had with an **adult** who had malaria.

- Where were you when you saw them?
  - **Probe: in usual clinical setting vs other e.g., home**
- How old were they?
- What was their gender?
- What symptoms were they showing?
- What questions did you ask?
  - Why did you ask this question?
- What questions did they ask?
  - Why do you think they wanted to know this?
- What happened next?
  - **Moderator to ask all of these probes:** Did you diagnose them?
    - **If so:** Did you use any specific diagnostic tests to diagnose them? **Probe: blood slide/ smear versus rapid diagnostic test**
      - **If so:** Which do you use?
        - Why did you use this test?
        - What did you see in this patient that made you think you should do this test?
      - **If so:** How did the patient react?
        - Did they have any questions about testing?

**If so:** What were they?

- **If not:** Why not?
- **Moderator to ask all of these probes:** Did you give them any treatments?
  - **If so:** What treatment did you give them?
  - How did you choose this treatment?
  - Did the patient ask for a specific treatment?
    - **If so:** What?
  - How did this patient respond to you giving them this medication?
- Did you give the patient any additional malaria-specific information or counselling, aside from treatment, if any?
- Did you discuss what to do with this patient with any of your colleagues?
  - **If so:** How, if at all, was this helpful?
- How typical is this experience of what you usually do when an adult patient comes to you with symptoms of malaria?

**Moderator say:** Thanks for telling me that. Now we’re going to talk a bit more about the decisions you have to make for an adult patient with malaria.

**Decision to test**

- How frequently, if at all, do you give an adult patient a test if they are experiencing symptoms of malaria?
  - What is the value of conducting a malaria diagnostic test?
  - How accurate do you think they are?
    - Why do you think this?
  - Are tests always available?
    - Why / why not?
  - ***Ability:*** How do you test? Talk me through what you have to do.
  - How did you learn what to do?
    - **Probe for: experience, information sources, guidelines**
  - What do you do if a test comes back negative?
- ***MAPPS:*** What, if at all, do you think the benefits of testing for malaria are?
- **MAPPS:** What if at all, do you think the drawbacks are?
- ***MAPPS:*** Think of a time you used a diagnostic test to test a patient for malaria.
  - Why did you give them this test?
  - Why was it important to give them this test?
    - What impact, if any, did this have on their treatment?
- ***MAPPS:*** Think of a time you did not offer a diagnostic test on a patient who was showing signs or symptoms of malaria.
  - Why didn’t you offer them a diagnostic test?
    - What impact, if any, did this have on their treatment?

**Decision to treat and recommend/prescribe**

**STIMULUS 5: TREATMENTS BUBBLE DIAGRAM**

- ***Ability:*** I would now like you to look at the diagram in front of you. Imagine that the person in the middle of the diagram is a 35 year old patient who is experiencing symptoms such as fever, chills and headaches. You conduct a test and it displays a positive result for malaria. **Moderator to write down** all of treatments the patient could have to treat these symptoms.
  - Which of these treatments are you most likely to use?
    - Why ?
  - Why wouldn’t you use the other treatments you have listed?
  - Do you know of any treatments that come in different formulations?
    - **Probe on IV, injectable, tablets, soluble tablets**
      - What, if anything, would be the benefits of using these different formulations?
- ***MAPPS:*** For this next exercise I’m going to say a category and you have to select one treatment that fit that category best.
  - Which is the treatment that…
    - You know best
    - You’re most comfortable with
    - Is easiest for you (as an HCP) to get hold of
    - Works the best
    - Patients are happiest with
    - Is most affordable for your patient group
    - You are keen to try if you could
- **If not mentioned:**
  - What about home remedies? What, if anything, do you suggest to patients as to what they can take or do / not do at home **Probe: sleep, hydrate, warm water etc.**

**Engaging patients with adherence**

- ***MAPPS:*** For this next task, I would like to do a role playing activity. Imagine that I’m a patient who has confirmed to have malaria and you have prescribed a treatment you typically choose to me. What do you say to me next?

**Ask HCP to start off with giving instructions on their typical treatment choice.**

**Moderator then asks these questions as a patient:**

- ***Motivation:*** Why should I follow your instructions?
  - What will happen if I don’t take all the treatment?
  - **Probe on treatment resistance if not mentioned**
- ***Ability:*** If I forget to take one, what do I do?
- ***Motivation:*** How do I know if the treatment has worked?
- ***Motivation:*** My friend said I should stop taking them if I feel better so I can save some, is this true?
- ***Motivation***, ***Processing***: I’m a little confused as that was a lot of information to take in. What are the 3 most important things I should know or do?

**Moderator say:** Ok thank you, we will stop the role play now. Reflecting on the role play:

- Are there any other questions that patients typically ask?
- How often, if at all, do you have conversations with your patients about the importance of taking their treatment everyday?
  - Why?
  - When are you most likely to have a conversation with a patient about making sure they adhere to their medication?
  - When are you least likely to have a conversation with a patient about making sure they adhere to their medication?
- ***Ability,*** ***Social***: If a patient asks a question that you can’t answer, what do you do?
  - **Probe: asking other HCPs, look at information resources, direct patients to resources**
- Of course, our roleplay was just between you and me (patient and HCP). But does anyone else play a part in ensuring that a patient takes their medication?
  - **Probe: other HCPs, caregiver’s family and/or friends, community leaders**
- ***Motivation:*** Do you ever know if the patient has completed their full course?
  - How do you know?
    - **Probe: follow up appointments/interactions, reporting**
  - How important, if at all, is it for you to know if a patient has completed their full course?
    - Why?
- ***Motivation*, *Ability*: If not mentioned:** Just before we move on, we’ve spoken to some other **[moderator say specialty]** who have mentioned anti-malarial resistance. Can you tell me what you know about it?

**SECTION 4 – Drivers of HCP behaviour across the patient pathway (15 minutes)**

**Objective:** To understand the differences in influences on HCP behaviours relating to diagnosis, treatment and encouragement of adherence for infant/child patients and their caregivers. (All MAPPS dimensions)

**Moderator say:** So you mentioned earlier that you’ve seen [insert proportion mentioned in section 2] babies and [insert proportion mentioned in section 2] children with malaria in the last malaria season

**Throughout this section, start with whichever age group they see more of. Use this as a reference point and ask about other age groups.**

***MAPPS:*** So you’ve told me about the last appointment/interaction with an adult patient with malaria. Now I’d like you to tell me about the last experience you had with a **[moderator to choose age group they see most of amongst babies/infants]**.

- Where were you when you saw them?
  - **Probe: in usual clinical setting vs other e.g., home**
- How old were they?
- What was their gender?
- Was anybody with them?
  - Who?
- How do you think they / their caregiver was feeling?
- What symptoms were they showing?
- What questions did you ask?
  - Why did you ask this question?
- What questions did they ask?
  - Why do you think they wanted to know this?
- What happened next?
  - **Moderator to ask all of these probes:** Did you diagnose them?
  - **If not mentioned:** Did you use any tests?
    - **If so:** Which do you use?
      - Why did you use this test?
      - What did you see in this patient that made you think you should do this test?
    - **If so:** How did the patient react?
      - Did they have any questions about testing?

**If so:** What were they?

- **If not:** Why not?
- **Moderator to ask all of these probes:** Did you give them any treatments?
  - **If so:** What treatment did you give them?
  - How did you choose this treatment?
  - How did you explain this treatment to the patient?
    - **Probe: dosage/timing, finishing full treatment**
  - How, if at all, was this different to what you did with the adult you mentioned earlier?

**Moderator to compare throughout and bring up what HCPs said for the adult earlier if there are any differences to understand why these differences exist.**

**Decision to test**

- You said earlier you would **[moderator to recall what respondent said earlier about testing** test all adults / only test some adults / not test any**]**, would you do the same here with **[moderator to choose age group they see most of amongst babies/infants first]**?
  - **Moderator to tailor probes depending on which group they have already asked about**
    - Would this be the same for babies under 5kg?
      - Why?
    - What about infants 1-6 years old?
      - Why?
    - And children 7-12 years old?
      - Why?
    - And finally, what about teenagers how would this differ?
      - Why?
- Thinking back to what you said the benefits are for testing. **[Moderator to list benefits that the respondent mentioned earlier.]**
  - Do the same apply to **[moderator to choose age group they see most of amongst babies/infants first]?**
    - Why / why not?
  - Is there anything you would like to add or remove from this list?
    - **If so:** Why?
    - Would this be the same for babies under 5kg?
    - What about infants 1-6 years old?
    - And children 7-12 years old?
    - And finally, what about teenagers how would this differ?

**Decision to treat and prescribe**

- ***MAPPS:*** What treatments would you consider for **[moderator to choose age group they see most of amongst babies/infants first]?**
  - Why would you consider these?
- Are there any different formulations that you would consider?
  - **Probe on tablets, soluble, IV to understand why they would or wouldn’t consider each one**
  - **Moderator to tailor probes depending on which group they have already asked about**
    - Would this be the same for babies under 5kg?
      - Do you discuss potential safety issues with the caregivers due to dose adjustments of antimalarials for infants weighing less than 4.5kg?
        - **If yes:** What do you say?
    - What about infants 1-6 years old?
    - And children 7-12 years old?
    - And finally, what about teenagers how would this differ?
- Which, if any, treatments do you not recommend?
  - **Moderator to tailor probes depending on which group they have already asked about**
    - Would this be the same for babies under 5kg?
    - What about infants 1-6 years old?
    - And children 7-12 years old?
    - And finally, what about teenagers how would this differ?

**Engaging patients with adherence**

- ***MAPPS:*** How would you describe your recommended treatment to a caregiver?
  - What, if anything, would you tell them about it?
  - How important is it, if at all, for the caregiver to make sure the child completes the full course of medication?
    - Why?
  - How, if at all, would you know if the child had taken all of the medication?
- Earlier we spoke about what you would tell an adult if they were the one taking malaria medication. How, if at all, is the conversation different when you’re talking to the caregiver?
  - What, if any, types of questions do they ask?
    - How, if at all, are these different to the questions adults ask when they are experiencing symptoms themselves?

**SECTION 4 – Close (2 minutes)**

**Objective:** To capture HCP suggestions as to what might help them in their role and to bring the discussion to a close

**Moderator say:** Just before we finish, if you had a magic wand that could grant you 1 wish**,** what would you wish to make your life easier when it comes to your practise in relation to malaria?

- How would that make your life easier?

**Thank and close**
