# Supplementary material for: Understanding barriers to effective malaria diagnosis and treatment: A mixed-methods study in Uganda, Nigeria, and Ivory Coast
Source: PLOS Glob Public Health. 2026 Aug 17;6(8):e0006921. doi: 10.1371/journal.pgph.0006921 (PMC13480639; doi:10.1371/journal.pgph.0006921)
Supplement: Supplementary file 2 [file pgph.0006921.s002.docx]

**S2 Text: Survey instruments**

Ipsos

3 Thomas More Square, London, E1W 1YW, UK

Tel: + 44 20 3059 5000 Fax: +44 20 3059 4998

Internal / client use only

# BeSci Malaria Pathway

# Caregivers Questionnaire

**Online survey**

**30 minutes**

**Sample**

|  | **Uganda** | **Ivory Coast** | **Nigeria** |
| --- | --- | --- | --- |
| **Parents/Caregivers** | **150** | **150** | **150** |

**Quotas**

| **SDistrict** | **Uganda** | **Ivory Coast** | **Nigeria** |
| --- | --- | --- | --- |
| **Sokoto** | **N/A** | **N/A** | **75** |
| **Taraba** | **N/A** | **N/A** | **75** |
| **Acholi** | **75** | **N/A** | **N/A** |
| **East-Central Busoga** | **75** | **N/A** | **N/A** |
| **Bas-Sassandra** | **N/A** | **75** | **N/A** |
| **La Vallee Du Bandaman** | **N/A** | **75** | **N/A** |

| **SSetting** | **Uganda** | **Ivory Coast** | **Nigeria** |
| --- | --- | --- | --- |
| **Urban** | **50** | **50** | **50** |
| **Rural** | **100** | **100** | **100** |

| **SAgeOfChildren** | **Uganda** | **Ivory Coast** | **Nigeria** |
| --- | --- | --- | --- |
| **0-3 months** | **Minimum 30** | **Minimum 30** | **Minimum 30** |
| **4-6 months** | **Minimum 30** | **Minimum 30** | **Minimum 30** |
| **7-12 months** | **20** | **20** | **20** |
| **1-4 years** | **20** | **20** | **20** |
| **5-7 years** | **Minimum 15**  **Maximum 25** | **Minimum 15**  **Maximum 25** | **Minimum 15**  **Maximum 25** |
| **8+ years** | **Minimum 15**  **Maximum 25** | **Minimum 15**  **Maximum 25** | **Minimum 15**  **Maximum 25** |

| **Signed off for scripting / translation by:** | |
| --- | --- |
| **AD/D** |  |
| **PM** |  |

# Screener

S0. What is your interviewer ID number?

_____________________________________

[PN: OPEN NUMERIC]

SCountry. In which country does the respondent live?

(Please select one answer only)

- Uganda
- Nigeria
- Ivory Coast
- None of these

[PN: SINGLE CODE. CLOSE IF ‘NONE OF THESE’ SELECTED. DO NOT SHOW TO RESPONDENTS]

SIntro. This survey is being conducted by Ipsos, a research agency, on behalf of a pharmaceutical company. We are conducting research to understand the access and adherence to malaria treatment. Your data will be analysed only when aggregated with other respondents’ data and will not be identifiable to you. This analysis will be used to understand more about healthcare practices in your region and to inform potential healthcare interventions. We would like to ask you some questions on this topic. The survey will take approximately 30 minutes of your time.

This research is intended to understand your experiences with and in relation to malaria. We will also ask you about treatments that may become available in the future. Please note that your participation is voluntary and you may stop the survey at any time and we will destroy the data we collect.

With your consent, your information will only be collected and used for social research and not given to anyone else**.** Any information you give will be treated with care to make sure nobody can see the responses you gave came from you. When we share information with the company who has asked for this research, we will remove any data which could identify you. We may need to send some of your personal data outside of your country of residence. We will make sure that it is always kept secure.

As a member of the Market Research Society (MRS), Ipsos is bound by the MRS Code of Conduct and all applicable laws protecting your personal data and responses. The study is conducted in compliance with local guidelines and codes of conduct. You can decide not to take part in the study at any time.

If you mention any side effects with a medicine that our client makes or any product complaints, we must report this to the client. This is to help them learn more about the safety of their products. If this happens, we will need to collect details and report the side effects or product complaint.

You will be asked if you give your permission for us to pass your details to the company’s drug safety department, but you can also choose to remain anonymous if you wish. Even if you choose to give your name to the company related to an issue with your medicine, the rest of your interview will remain confidential and anonymous.

Are you happy to participate in this research as described?

(Please select one answer only)

- Yes, I wish to continue
- No, I do not wish to continue

[PN: SINGLE CODE. CLOSE IF ‘NO’ SELECTED]

SSpecial. Do you consent to providing information relating to malaria as described above?

- Yes, I consent to providing information as described above.
- No, I do not consent to providing information as described above.

[PN: SINGLE CODE. CLOSE IF ‘NO’ SELECTED]

SDistrict. In which district do you spend most of your time?

(Please select one answer only)

**Recruit to soft quotas**

**[PN: Show if SCountry = NIGERIA]**

- Sokoto
- Taraba
- Other

**[PN: Show if SCountry = UGANDA]**

- Acholi
- East-Central Busoga
- Other

**[PN: Show if SCountry = IVORY COAST]**

- Bas-Sassandra
- La Vallee Du Bandaman
- Other

**[PN: SINGLE CODE. CLOSE IF OTHER SELECTED]**

SArea. Which of the following best describes the area where you live?

(Please select one answer only)

- Urban
- Rural
- Not sure **[ASSIGN RANDOMLY TO URBAN/RURAL SOFT QUOTA]**

[PN: RANDOMISE, RECRUIT 30% URBAN AND 70% RURAL]

SAge. What was your age at your last birthday?

(Please type in respondent’s answer)

- Range 0-100

[PN: OPEN NUMERIC. 0-100. CLOSE IF <18]

SChildren. How many children do you have in your permanent care?

Please think only of children for whom you are involved in healthcare decisions.

(Please type in your answer)

- Open numeric 1-30
- I do not have any children
- Prefer not to say [PN: EXCLUSIVE, SINGLE CODE]

[PN: OPEN NUMERIC, CLOSE IF I DO NOT HAVE ANY CHILDREN OR PREFER NOT TO SAY IS SELECTED]

SAgeOfChildren. Thinking about the children you have in your care, how old is the child that most recently experienced malaria within the last 5 years?.

Please state the answer in either years or months only.

- Open numeric 1-17 years OR Open numeric 0-12 months
- 18+ years
- Prefer not to say [PN: EXCLUSIVE]
- None of the children in my care have experienced malaria in the last 5 years

[PN: OPEN NUMERIC, CLOSE IF 18 YEARS OR ABOVE SELECTED OR PREFER NOT TO SAY IS SELECTED OR ‘NONE OF MY CHILDREN’ IS SELECTED. ASSIGN RESPONDENT TO ONE AGE GROUP AND RECRUIT PER SAGE CHILDREN QUOTA]

SRoleInCare. For this child, please select the option that best describes your relationship to them.

- I am their parent
- I am their aunt or uncle
- I am their grandparent
- I am their step-parent or adoptive parent
- Other
- Prefer not to say

[PN: SINGLE CODE, CLOSE IF SELECTING ‘Other’ OR ‘PREFER NOT TO SAY’]

**SResearch.** Have you taken part in any market or social research about malaria in the last month?

- Yes
- No

**[PN: SINGLE CODE, CLOSE IF YES SELECTED]**

# Section A: Demographics

**A1.** Which of the following best describes your gender?

- Man
- Woman
- In another way
- Prefer not to say

**[PN: SINGLE CODE]**

A2. Thinking about the child in your care who most recently experienced malaria , which of the following best describes their gender?

- Boy
- Girl
- In another way
- Prefer not to say

[PN: SINGLE CODE]

A3. What best describes your employment status?

(Please select one answer only)

- Full time employment (30+ hours per week)
- Part time employment (8-29 hours per week)
- Student
- Unemployed
- Retired
- Other [PN: EXCLUSIVE]
- Prefer not to say [PN: EXCLUSIVE]

[PN: SINGLE CODE]

A4. [UGANDA AND NIGERIA ONLY] What is your usual **annual** household income taken home (e.g after tax)?

[IVORY COAST ONLY] What is your usual **monthly** household income taken home (e.g after tax)?

(Please select one answer only)

[PN: SHOW IN UGANDA ONLY]

- Below UGX 500,000
- UGX 500,001 - UGX 1,000,000
- UGX 1,000,001 - UGX 1,500,000
- UGX 1,500,001 - UGX 2,000,000
- Above UGX 2,000,001
- Prefer not to say
- Don’t know

[PN: SHOW IN IVORY COAST ONLY]

- Rien/aucun revenu
- Less than 20 000 FCFA
- 20 000 - 39 999 FCFA
- 40 000 - 59 999 FCFA
- 60 000 - 99 999 FCFA
- 100 000 - 299 999 FCFA
- 300 000 - 449 999 FCFA
- 450 000 -799 999 FCFA
- 800000 FCFA and above
- Prefer not to say
- Don’t know

[PN: SHOW IN NIGERIA ONLY]

- Less than N360, 000
- N360,000 - N1,500,000
- N1,500,001 - N3,000,000
- N3,000,001 - N6,000,000
- More than N6,000,001
- Prefer not to say
- Don’t know

[PN: SINGLE CODE]

A4. Which of the following best describes your highest level of education?

- No formal education
- Completed primary education
- Completed secondary education
- Completed college or technical school
- Completed undergraduate degree
- Completed postgraduate degree (Master's, PhD, etc.)
- Prefer not to answer

[PN: SINGLE CODE]

A5. Which of the following best describes your marital status?

- Single, never married
- Married
- Living with partner
- Separated
- Divorced
- Widowed
- Prefer not to answer

[PN: SINGLE CODE]

**A6.** Do any of the following roles apply to you?

- Religious Leader
- Local Council Member
- Government Official
- Other **– OPEN END**
- None of these roles apply to me **[EXCLUSIVE]**

**[PN: MULTICODE]**

**A7.** Have you yourself had malaria within the past 5 years?

- Yes
- No
- I don’t know **[EXCLUSIVE]**

**[PN: SINGLE CODE]**

# Section B: Malaria

For the next set of questions, I would like you to think of the **child in your care who most recently experienced malaria**.

**B1.** When was their last experience with malaria?

- Less than a month ago
- 1-5 months ago
- 6-12 months ago
- 1-5 years ago

**[PN: SINGLE CODE]**

**B2.** Thinking about the last time this child had malaria, which of the following did you do after realising their symptoms might indicate malaria?

(Please select all that apply)

- I waited a few days for the symptoms to go themselves
- I went to a healthcare facility to get medication (including antimalarials)
- I went to a healthcare facility to get a test to confirm malaria
- I went to a traditional healer for advice
- I went to a traditional healer for treatment
- I went to a community leader for advice
- I went to a community leader for treatment
- I treated their symptoms at home with paracetamol I had already
- I treated their symptoms at home with antimalarials that I had already
- I treated their symptoms at home with traditional medicine
- Other **- FIXED**
- Don’t know **- FIXED**

**[PN: MULTICODE, RANDOMISE]**

B3. [ASK MORE THAN 1 SELECTED AT B2] Please rank the following actions in the order of which you did them.

- [PIPE IN ANSWERS FROM B2]

[PN: MULTICODE, RANDOMISE]

B4. [IF WENT TO A HEALTHCARE FACILITY AT B2] When they last had malaria, which of the following healthcare facilities did you visit?

- Pharmacy
- Public clinic
- Private clinic
- Public hospital
- Private hospital
- I did not visit any of the facilities above [EXCLUSIVE]

[PN: MULTICODE, RANDOMISE]

B5. [IF WENT TO A HEALTHCARE FACILITY AT B2] For the healthcare facility that you visited, how long did it take you to get to there by your usual means of travel?

[PN: TOP, RESPONSES]

- [PIPE IN ANSWERS FROM B4]

[PN: SIDE, STATEMENTS]

- <30 minutes
- 30 minutes – 1 hour
- 1-2 hours
- 3-5 hours
- >5 hours
- Don’t know

[PN: SINGLE CODE]

B6. Thinking about the child in your care who most recently experienced malaria, which of the following best describes their experience?

(Please select one answer only)

- Their **healthcare professional offered** to test them to confirm their diagnosis
- I **asked a healthcare professional** for a test to confirm **their** diagnosis
- Their diagnosis **was not** confirmed by a test

[PN: SINGLE CODE]

**B7.** How often, if at all, do you worry about the possibility of this child catching malaria?

- Almost always
- Often
- Sometimes
- Never or rarely
- Don’t know

**[PN: SINGLE CODE]**

**B8.** In the last 6 months, which of the following have you done in the last 6 months to prevent malaria, **for the child in your care who has most recently had malaria**?

[PN: TOP, RESPONSES]

- Always
- Sometimes
- Rarely
- Never
- Don’t know

[PN: SIDE, STATEMENTS]

- I ensure the child I care for uses mosquito nets
- I ensure the child I care for uses insect repellent
- I regularly clean the surroundings to prevent mosquito breeding
- I ensure there is air conditioning or fans are working in our residence
- I make sure the child I care for does not come into contact with stagnant water
- I make sure the child I care for wears long sleeves and trousers
- Other (please specify) **– OPEN END**

[PN: SINGLE CODE PER STATEMENT]

# Section C: Access

**C1.** Please indicate whether the following symptoms may or may not indicate malaria.

**[PN: TOP, RESPONSES]**

- May indicate malaria
- Does not indicate malaria
- Don’t know

**[PN: SIDE, STATEMENTS]**

- High fever
- Shaking chills
- Profuse sweating
- Headache
- Abdominal pain
- Muscle pain
- Nausea
- Vomiting
- Diarrhoea
- Rash
- Cough
- Change in skin tone
- Loss of sense of taste
- Loss of sense of smell
- Other (please specify) **[OPEN TEXT]**

[PN: MULTICODE, RANDOMISE STATEMENTS]

**C2. [ONLY IF MORE THAN 3 DO INDICATE MALARIA AT C1]** Of those that you said may indicate malaria, which three symptoms are most likely to indicate malaria to you?**[PIPE IN RESPONSES FROM C1 THAT DO INDICATE MALARIA]**

[PN: MULTICODE, MUST SELECT 3, RANDOMISE STATEMENTS]

**SEEKING CARE**

**C3.** There are several reasons why people **may or may not seek care**, advice or treatment for their children when they first present symptoms that might suggest malaria. To understand your opinions and attitudes about this, please state the extent to which you agree or disagree with the following statements.

Please think about the child in your care who most recently experienced malaria when answering these questions.

[PN: GRID, USE QUESTION LIBRARY FI011]

[PN: TOP, RESPONSES]

- Strongly Agree
- Agree
- Neither agree nor disagree
- Disagree
- Strongly Disagree
- Don’t know

[PN: DO NOT SHOW TITLE] MOTIVATION – EMOTION

- I get scared if the child gets symptoms that could indicate malaria

[PN: DO NOT SHOW TITLE] MOTIVATION – OUTCOME EXPECTATIONS

- Paracetamol gets rid of the child’s malaria symptoms, just as well as a different treatment they could get from the clinic or pharmacy
- Seeking care from a healthcare professional will help the child recover from malaria
- It’s easy for me to get professional help for the child’s malaria symptoms
- I am confident that a healthcare professional can give me the right advice on what to do to treat the child’s malaria
- Their malaria symptoms will resolve by themselves

**[PN: RANDOMISE WITHIN SUBGROUP]**

**[PN: DO NOT SHOW TITLE]** **ABILITY**

- I can easily identify early symptoms of malaria in the child
- It can be difficult to distinguish the symptoms of malaria from other conditions
- I know where to go for healthcare advice when the child is experiencing malaria symptoms
- I know which healthcare facility to go to when the child is experiencing malaria symptoms
- It is important to seek care for the child’s malaria symptoms early to avoid complications in the future

**[PN: RANDOMISE WITHIN SUBGROUP]**

**[PN: DO NOT SHOW TITLE]** **PHYSICAL**

- The healthcare facility I would like to go to is too far away for me to consider it
- My work and/or childcare responsibilities make it difficult for me to take the child to a healthcare facility quickly

**[PN: DO NOT SHOW TITLE]** **SOCIAL**

- Other people around me do not prioritize going to the clinic for advice when their children experience malaria symptoms
- In my community, it is more important to seek care for other diseases instead of malaria
- In my community, seeking health advice from a traditional healer is preferred instead of going to the clinic

**[PN: RANDOMISE WITHIN SUBGROUP]**

**WHERE TO SEEK CARE**

**C4.** We’d like to know a bit more about how you feel when you’re deciding **where to seek care** when the child in your care who most recently experienced malaria is experiencing symptoms that might be from malaria. Please state the extent to which you agree or disagree with the following statements.

Again, please think about the child in your care who most recently experienced malaria when answering these questions.

**[PN: TOP, RESPONSES]**

- Strongly agree
- Somewhat agree
- Neither agree nor disagree
- Somewhat disagree
- Strongly disagree
- Don’t know

**[PN: SIDE, STATEMENTS]**

**[PN: DO NOT SHOW TITLE] MOTIVATION**

- I believe the pharmacist can treat the child’s malaria symptoms better than a healthcare professional at a hospital or clinic
- I am confident that going to a qualified healthcare professional for advice is the best option when the child is experiencing malaria symptoms
- I believe it's worth the time and cost to travel to the hospital or clinic to get tested for malaria before being treated
- I believe it's worth the time and cost to travel to the hospital or clinic to get treated for malaria
- I prefer going to the pharmacy for malaria because I can get the medication thechild needs right away without a prescription
- I trust the pharmacist's advice on which malaria medication to give the child, so I don't feel the need to see a doctor first

**[PN: DO NOT SHOW TITLE] ABILITY**

- I'm used to describing my child's malaria symptoms to the pharmacist and purchasing treatment medication for them without getting them tested first
- I’m used to describing my child’s malaria symptoms to the pharmacist
- I’m used to purchasing treatment medication for them without getting them tested first

**[PN: DO NOT SHOW TITLE] PHYSICAL**

- It's quicker for me to take the child to a pharmacy than a hospital or clinic when they are experiencing malaria symptoms
- It's cheaper for me to take the child to a pharmacy than a hospital or clinic when they are experiencing malaria symptoms
- There is an accessible hospital/clinic close to my home that I can take the child to if they are experiencing malaria symptoms
- There are transport options available for me to take the child to the hospital or clinic if they develop malaria symptoms
- Pharmacies are much closer to my home than the nearest hospital or clinic
- I can get malaria medication at a lower cost from the pharmacy compared to the consultation and prescription fees at a hospital or clinic
- **[PN: ONLY SHOW IF SCHILDREN > 1]** It can be difficult to take my child to a healthcare facility because I need to spend time looking after my other children
- I set aside money in case I need to cover the cost of travel or treatment if the child does get sick

**[PN: DO NOT SHOW TITLE] SOCIAL**

- Most people in my community self-treat their children at home rather than going to a hospital or clinic for malaria treatment
- I prefer to seek care for the child from a community leader first, before seeking care from a trained healthcare professional
- Community leaders encourage us to seek professional medical care immediately for the children we care for if we suspect malaria rather than waiting

**GETTING TESTED**

**C5.** We’d like to know a bit more about your attitudes and beliefs when it comes to the child in your care who most recently experienced malaria receiving a diagnostic test for malaria. Please state the extent to which you agree or disagree with the following statements.

**[PN: TOP, RESPONSES]**

- Strongly agree
- Somewhat agree
- Neither agree nor disagree
- Somewhat disagree
- Strongly disagree
- Don’t know

**[PN: SIDE, STATEMENTS]**

**[PN: DO NOT SHOW TITLE] MOTIVATION**

- I believe getting the child tested is the only way to confirm if they really have malaria
- I am confident that getting my child tested will help them receive the right treatment for their condition
- I believe that getting the child tested for malaria will save me time in the future
- I believe that getting the child tested for malaria will save me money in the future
- I believe getting my child tested for malaria helps to restrict the spread of the disease within the community

**[PN: RANDOMISE CODES WITHIN SUBGROUP]**

**[PN: DO NOT SHOW TITLE] ABILITY**

- I know where to take my child to get a malaria test
- It is important to get tested to confirm the diagnosis
- I know when to take my child to get a malaria test, if necessary

**[PN: RANDOMISE CODES WITHIN SUBGROUP]**

**[PN: DO NOT SHOW TITLE] PHYSICAL**

- I can afford the cost of a malaria test for the child
- Malaria testing is easy to get at a healthcare facility near me
- The process of getting tested for malaria is quick

**[PN: RANDOMISE CODES WITHIN SUBGROUP]**

# Section D: Adherence

The next few questions will focus on treatments for malaria. Again, please think about the child in your care who most recently experienced malaria when answering these questions.

D1. Thinking about the child in your care who most recently experienced malaria, which of the following treatments did they take?

[PN: SIDE, STATEMENTS]

- Traditional treatments
- Antimalarial pills prescribed by a doctor
- Antimalarial pills I was given by a friend or a family member
- Antimalarial pills I already had in my possession
- Antimalarials pills I bought from a shop
- Antimalarial injection
- Paracetamol
- Iron tablets
- Other

[PN: SINGLECODE PER LINE, RANDOMISE STATEMENTS]

D2. Thinking about the the child in your care who most recently experienced malaria and took antimalarial treatment, which of the following best describes how they took the treatment?

- The child took all of their antimalarial treatment
- The child took some of their antimalarial treatment
- The child did not take their antimalarial treatment
- Don’t know

[PN: SINGLECODE, RANDOMISE STATEMENTS]

D3. [IF D2 = I TOOK SOME OF MY ANTIMALARIALS] Thinking about the child in your care who most recently experienced malaria and took antimalarial treatment, which of the following best describes why they stopped taking their medication early?

- They forgot to take the rest of their medication
- They didn’t want to take the medication because of the taste
- I stopped giving it to them when their symptoms disappeared
- I stopped giving it to them because they did not help relieve their symptoms
- I stopped giving it to them because the medication made them feel unwell
- I wanted them to save some treatment for next time
- Don’t know

[PN: SINGLECODE, RANDOMISE STATEMENTS]

D4. There are several reasons why children may or may not take all their malaria treatment. To understand your attitudes and opinions about this, please state the extent to which you agree or disagree with the following statements. When answering these questions, please think about antimalarial medication the child in your care who most recently experienced malaria may take to treat their malaria.

[PN: GRID, USE QUESTION LIBRARY FI011]

[PN: TOP, RESPONSES]

- Strongly Agree
- Agree
- Neither agree nor disagree
- Disagree
- Strongly Disagree
- Don’t know

[PN: SIDE, STATEMENTS]

[PN: DO NOT SHOW TITLE] MOTIVATION – OUTCOME EXPECTATIONS

- Once the child’s malaria symptoms are gone, there is nothing left for the treatment to do

[PN: DO NOT SHOW TITLE] MOTIVATION – EMOTION

- I find having to force the child to take their malaria treatment difficult when I know they don’t want to take it

[PN: DO NOT SHOW TITLE] ABILITY – KNOWLEDGE

- It is fine to stop the child's treatment when their symptoms are gone, because it means that the disease is gone
- I know the reasons why the child needs to take the full course of treatment
- I know exactly how often and when my child should take their antimalarial treatment

[PN: DO NOT SHOW TITLE] ABILITY – ROUTINE

- It can be difficult to remember to give the child all of the doses of antimalarial treatment
- I can’t always give the child all of the doses at the right time if they are out of the house

[PN: DO NOT SHOW TITLE] PHYSICAL – ENVIRONMENTAL

- The taste of antimalarial pills makes them difficult for the child to take
- My child finds antimalarial pills difficult to swallow because they are so large
- Malaria treatment is expensive
- Stopping the child's malaria treatment early means that I can save the treatment for next time, which saves me money
- Stopping the child's malaria treatment early means that I don’t have to see a healthcare professional next time they have malaria
- I find it difficult to trust the child to take their medication without me reminding them

[PN: DO NOT SHOW TITLE] SOCIAL – SOCIAL

- People I know have stopped giving their child their antimalarial treatment early and the child still recovered
- People I know believe one should give their child all of the antimalarial treatment, even when the child's symptoms disappear

[PN: MULTICODE, RANDOMISE STATEMENTS]

# Section F: Pharmacovigilance Re-contact

F1. The sponsoring company of this research has an obligation to continuously monitor the safety of their products. Although rare, their Product Safety Department may want to further investigate any issues you may have raised in this survey. If we identify any adverse event/product complaint mentioned by you during this research, are you willing to be contacted by us to provide more information regarding the adverse event/product complaint?

(Please select one answer only)

- Yes, I am willing to be contacted to provide more information regarding the adverse event/product complaint
- No, I am not willing to be contacted to provide more information regarding the adverse event/product complaint

[PN: SINGLE CODE. SHOW IF CLOSED-END AE TRIGGERED WITHIN SURVEY OR IF OPEN-ENDED QUESTIONS IN QUESTIONNAIRE. FOR OPEN-ENDED QUESTIONS WHICH REQUIRE MANUAL REVIEW FOR AEs, USE F2 FOR OFF-LINE RE-CONTACT OF RESPONDENTS TO ASK FOR CONSENT TO PASS ON PERSONAL DATA TO CLIENT IN ALL MARKETS]

F2. Would you be willing to have your personal data passed on to the sponsoring company for the purpose of obtaining more information regarding any adverse event / product complaints mentioned in this research? Please note that if you were to consent to having your personal data passed on to the sponsoring company, Novartis, such personal data will be controlled and processed by their Product Safety Department. Their privacy notice that can be reviewed at this link LINK.

(Please select one answer only)

- Yes, I consent to my personal data being passed on to Novartis.
- No, I do not consent to my personal data being passed on to Novartis.

[PN: SINGLE CODE. SHOW ONLY IF CLOSED-END AE TRIGGERED WITHIN SURVEY AND ‘YES’ AT F1. IF NO CLOSED-END AE TRIGGER BUT WITH OPEN-ENDED QUESTIONS WHICH REQUIRE MANUAL REVIEW FOR AEs, USE FOR OFF-LINE RE-CONTACT OF RESPONDENTS TO ASK FOR CONSENT TO PASS ON PERSONAL DATA TO CLIENT AS REQUIRED]

Ipsos

3 Thomas More Square, London, E1W 1YW, UK

Tel: + 44 20 3059 5000 Fax: +44 20 3059 4998

Internal / client use only

# BeSci Malaria Pathway

# HCP Questionnaire

**Online survey**

**30 minutes**

**Sample**

**HCPs split 50:50 between districts in each country**

|  | **Uganda** | **Ivory Coast** | **Nigeria** |
| --- | --- | --- | --- |
| **Nurse** | **50** | **50** | **50** |
| **Community health worker** | **50** | **50** | **50** |
| **Pharmacist** | **100** | **100** | **100** |

**Quotas**

| **SDistrict** | **Uganda** | **Ivory Coast** | **Nigeria** |
| --- | --- | --- | --- |
| **Sokoto** | **N/A** | **N/A** | **100** |
| **Taraba** | **N/A** | **N/A** | **100** |
| **Acholi** | **100** | **N/A** | **N/A** |
| **East-Central Busoga** | **100** | **N/A** | **N/A** |
| **Bas-Sassandra** | **N/A** | **100** | **N/A** |
| **La Vallee Du Bandaman** | **N/A** | **100** | **N/A** |

| **SDistrict** | **Uganda** | **Ivory Coast** | **Nigeria** |
| --- | --- | --- | --- |
| **Urban** | **60** | **60** | **60** |
| **Rural** | **140** | **140** | **140** |

| **Signed off for scripting / translation by:** | |
| --- | --- |
| **AD/D** |  |
| **PM** |  |

# Screener

S0. What is your interviewer ID number?

_____________________________________

[PN: OPEN NUMERIC]

SCountry. In which country does the respondent live?

(Please select one answer only)

- Uganda
- Nigeria
- Ivory Coast
- None of these

[PN: SINGLE CODE. CLOSE IF ‘NONE OF THESE’ SELECTED. DO NOT SHOW TO RESPONDENTS]

SIntro. This survey is being conducted by Ipsos, a research agency, on behalf of a pharmaceutical company. We are conducting research to understand the access and adherence to malaria treatment. We would like to ask you some questions on this topic. The survey will take approximately 30 minutes of your time.

This research is intended to understand your experiences with and in relation to malaria. We will also ask you about treatments that may become available in the future. Please note that your participation is voluntary and you may stop the survey at any time and we will destroy the data we collect from you.

With your consent, your information will only be collected and used for social research and not given to anyone else**.** Any information you give will be treated with care to make sure nobody can see the responses you gave came from you. When we share information with the company who has asked for this research, we will remove any data which could identify you. We may need to send some of your personal data outside of your country of residence. We will make sure that it is always kept secure.

As a member of the Market Research Society (MRS), Ipsos is bound by the MRS Code of Conduct and all applicable laws protecting your personal data and responses. The study is conducted in compliance with local guidelines and codes of conduct. You can decide not to take part in the study at any time.

If you mention any side effects with a medicine that our client makes or any product complaints, we must report this to the client. This is to help them learn more about the safety of their products. If this happens, we will need to collect details and report the side effects or product complaint.

You will be asked if you give your permission for us to pass your details to the company’s drug safety department, but you can also choose to remain anonymous if you wish. Even if you choose to give your name to the company related to an issue with your medicine, the rest of your interview will remain confidential and anonymous.

Are you happy to participate in this research as described?

(Please select one answer only)

- Yes, I wish to continue
- No, I do not wish to continue

[PN: SINGLE CODE. CLOSE IF ‘NO’ SELECTED]

SSpecial. Do you consent to providing information relating to malaria as described above?

- Yes, I consent to providing information as described above
- No, I do not consent to providing information as described above

[PN: RECORD]

# Section A: Screener

SDistrict. In which district do you spend most of your time?

(Please select one answer only)

**Recruit to soft quotas**

**[PN: Show if SCountry = NIGERIA]**

- Sokoto
- Taraba
- Other

**[PN: Show if SCountry = UGANDA]**

- Acholi
- East-Central Busoga
- Other

**[PN: Show if SCountry = IVORY COAST]**

- Bas-Sassandra
- La Vallee Du Bandaman
- Other

**[PN: SINGLE CODE. CLOSE IF OTHER SELECTED]**

SSpecialty. Which of the following best describes your role as a healthcare provider?

(Please select one answer only)

- Nurse
  - A healthcare professional providing direct patient care, administering treatments, and educating patients. May have expanded roles in primary care, especially in rural areas.
- Community Health Worker (CHW)
  - A trusted community member trained to provide basic health services, education, and referrals. Bridges the gap between communities and formal healthcare.
- Pharmacist
  - A professional working in a pharmacy either in a private or public hospital or stand alone pharmacy, responsible for safe and effective medication use. Dispenses prescriptions, provides counselling, and manages supply chains. May have expanded roles in disease management.
- Other [PN: FIXED]

[PN: RECRUIT TO QUOTA, CLOSE IF OTHER SELECTED]

SYears. Approximately how many years have you been in this role?

(Please type in answer below)

- Range 0-100

[PN: OPEN NUMERIC. CLOSE IF <2/>35 YEARS]

SArea. Which of the following best describes the area where you work?

(Please select one answer only)

- Urban
- Rural
- Not sure **[ASSIGN RANDOMLY TO URBAN/RURAL QUOTA]**

[PN: RANDOMISE, RECRUIT 30% URBAN AND 70% RURAL]

SResponsibility. Which of the following responsibilities, if any, do you have?

(Please select all that apply)

- Discussing malaria symptoms with patients
- Diagnosing patients with malaria
- Recommending/prescribing medication to patients with malaria
- Follow up care for patients with malaria
- I do not have any of the responsibilities listed [EXCLUSIVE, FIXED]

[PN: MULTICODE. CLOSE IF I DO NOT HAVE ANY OF THE RESPONSIBILITIES LISTED SELECTED]

SPatients. In the last month, thinking of all the patients you have seen who have had malaria, approximately what percentage have been each of the following patient groups.

(Please type in the approximate percentage of each patient type you have)

- Infants and neonates (<12 months) <5kg: **[INSERT PERCENTAGE 0-100] %**
- Infants and neonates (<12 months) 5kg or more: **[INSERT PERCENTAGE 0-100] %**
- Children (1-5 years): **[INSERT PERCENTAGE 0-100] %**
- Children (6-12 years): **[INSERT PERCENTAGE 0-100] %**
- Teenagers (13-17 years): **[INSERT PERCENTAGE 0-100] %**
- Adults (>18 years): **[INSERT PERCENTAGE 0-100] %**

[PN: CLOSE IF CODES 1 AND 2 ARE 0, ENSURE SUMS TO 100]

SAffiliation. Are you currently affiliated with any pharmaceutical company, government regulatory agency, market research agency and/or advertising company?

(Please select one answer only)

- Yes
- No

[PN: SINGLE CODE, CLOSE IF YES]

# Section B: Demographics

A1. What was your age at your last birthday?

(Please type in respondent’s answer)

- Range 0-100

[PN: OPENT NUMERIC]

**A2.** Which of the following best describes your gender?

- Man
- Woman
- In another way
- Prefer not to say

**[PN: SINGLE CODE]**

A3. [NURSES ONLY] In what type of healthcare setting do you primarily work?

- Public clinic
- Private clinic
- Public hospital
- Private hospital
- Other

[PN: SINGLE CODE]

A4. [PHARMACISTS ONLY] In what type of healthcare setting do you primarily work?

- Pharmacy
- Drug store
- Public clinic
- Private clinic
- Public hospital
- Private hospital
- Other (please specify)

[PN: SINGLE CODE]

A5. On average, approximately how many total patients do you personally see per day?

- [PN: OPEN NUMERIC] patients

[PN: OPEN NUMERIC, ALLOW 10-300]

A6. And of those [ANSWER AT A5], approximately how many of those patients do you see for malaria / malaria related symptoms?

- [PN: OPEN NUMERIC] patients

[PN: OPEN NUMERIC, DO NOT ALLOW MORE THAN ANSWER GIVEN AT A5]

Section B: Malaria

B1. How worried, if at all, are you about the consequences of each of the following patient types catching malaria?

- [PN: TOP, RESPONSES]
- 1 – I’m not at all worried
- 2
- 3
- 4
- 5 – I’m very worried

[PN: SIDE, STATEMENTS]

- Infants and neonates (<12 months) <5kg
- Infants and neonates (<12 months) 5kg or more
- Children (1-5 years)
- Children (6-12 years)
- Teenagers (13-17 years)
- Adults (>18 years)

**[PN: SINGLE CODE PER ROW]**

B2. Please rank the following conditions from 1 to 5 based upon their disease burden on the community where you work, where 1 represents the lowest burden on the community, and 5 the highest burden. .

When answering this question please assess the impact of these diseases on the community as a whole, independently of any comorbidities a particular individual might have.

- ______ Malaria
- ______ HIV/AIDS
- ______ Tuberculosis
- ______ Diarrhoeal diseases
- ______ Respiratory infections

[PN: RESPONDENT MUST ASSIGN A UNIQUE RANK TO EACH CONDITION. NO TIES/DUPLICATES ALLOWED]

# Section C: Testing practices

C1. When it comes to [PN: INSERT PATIENT GROUP FROM BELOW], indicate which of the following symptoms would prompt you to conduct a diagnostic test to confirm a diagnosis for malaria.

[PN: TOP, RESPONSES]

- Would prompt me to test a patient for malaria
- Would not prompt me to test a patient for malaria
- Don’t know

[PN: SIDE, SYMPTOMS]

- High fever
- Shaking chills
- Profuse sweating
- Headache
- Abdominal pain
- Muscle pain
- Nausea
- Vomiting
- Rash
- Cough
- Change in skin tone
- Loss of sense of taste
- Loss of sense of smell

[PN: SINGLE RESPONSE PER SYMPTOMS, RANDOMISE, PLEASE LOOP THIS QUESTION FOR EACH OF THE FOLLOWING PATIENT GROUPS, INSERTING EACH INTO QUESTION TEXT – ADULTS 18 +, BABIES UNDER 5 KG, INFANTS 6 MONTHS – 6 YEARS, CHILDREN 7-17 YEARS]

We would like to learn a little bit more about your past experiences with patients.

C2. Please think of the last time you saw a patient who was presenting symptoms of malaria from each of the following age groups. During your consultation, which of the following, if any, did you do?

[TOP, RESPONSES]

- Infants and neonates (<12 months) <5kg
- Infants and neonates (<12 months) 5kg or more
- Children (1-5 years)
- Children (6-12 years)
- Teenagers (13-17 years)
- Adults (>18 years)

[SIDE, STATEMENTS]

- I offered the patient or their caregiver a rapid diagnostic test that confirmed a positive result for malaria
- I offered the patient or their caregiver a rapid diagnostic test that confirmed a negative result for malaria
- I offered the patient or their caregiver a microscopy test that confirmed a positive result for malaria
- I offered the patient or their caregiver a microscopy test that confirmed a negative result for malaria
- I offered the patient or their caregiver a rapid diagnostic test but the caregiver refused it
- I offered the patient or their caregiver a microscopy test but the caregiver refused it
- The patient or their caregiver asked me for a rapid diagnostic test to confirm their diagnosis
- The patient or their caregiver asked me for a microscopy test to confirm their diagnosis
- I did not offer the patient or their caregiver either a rapid diagnostic or microscopy test and so their diagnosis was not confirmed
- I am not able to test patients myself but I recommended they get a diagnostic test from another healthcare professional

[PN: SINGLE CODE. DO NOT ALLOW CODES 1/2/3/4 TO BE SELECTED WITH 9/10]

C3. [IF TEST NOT OFFERED [OPTION 9] FOR AGE GROUP] Which of the following best describes why you did not test these patients?

[TOP, RESPONSES, ONLY SHOW FOR EACH GROUP WHERE OPTION 5 SELECTED IN C2]

- Infants and neonates (<12 months) <5kg
- Infants and neonates (<12 months) 5kg or more
- Children (1-5 years)
- Children (6-12 years)
- Teenagers (13-17 years)
- Adults (>18 years)

[SIDE, STATEMENTS]

- I did not have a rapid diagnostic test kit in stock
- It takes too much time to do the rapid diagnostic test
- The patient’s symptoms were not sufficient for malaria
- The patient or their caregiver could not afford cost of rapid diagnostic test
- The patient or their caregiver refused the rapid diagnostic test for another reason, other than cost
- I do not see the value in testing a patient if I know that the symptoms indicate malaria
- Rapid diagnostic tests are not reliable
- I, or the test administrator, do not have sufficient qualification to perform the microscopy test
- It takes too much time to get results from a microscopy test
- I wanted to do a microscopy test but this was not available
- Other reason [FIXED]

[PN: MULTICODE, RANDOMISE]

C4. Whose responsibility is it to ensure patients receive a test to diagnose their malaria?

- It is my responsibility to ensure patients receive a test to diagnose their malaria
- It is both the patient or their caregiver and my responsibility as a healthcare provider to ensure the patient receives a test to diagnose their malaria
- It is solely the patient or their caregiver’s responsibility to ensure the patient receives a test to diagnose their malaria

[SINGLE CODE]

C5. Thinking about your usual practice, if you suspect a patient has malaria, to what extent do you agree or disagree with the following statements about the importance of malaria testing. When talking about malaria testing, we are referring to rapid diagnostic tests.

[PN: GRID, USE QUESTION LIBRARY FI011]

[PN: TOP, RESPONSES]

- Strongly Agree
- Agree
- Neither agree nor disagree
- Disagree
- Strongly Disagree
- Don’t know

[SIDE, STATEMENTS]

[PN: DO NOT SHOW TITLE] MOTIVATION – OUTCOME EXPECTATIONS

- If I am unable to confirm my patient’s diagnosis with a test, they might end up spending more on their treatment
- If I am unable to confirm my patient's diagnosis with a test, their health might be negatively impacted
- If I am unable to confirm my patient’s diagnosis with a test, it is more difficult for me to know what treatment to give them
- Rapid diagnostic tests for malaria are unreliable
- Diagnostic tests conducted in the lab are more reliable than rapid diagnostic tests
- It is important to act quickly if I suspect a patient has malaria

[PN: DO NOT SHOW TITLE] MOTIVATION – SELF EFFICACY

- I find it challenging to administer malaria rapid diagnostic tests to patients
- I need some support in interpreting the results of malaria rapid diagnostic tests

[PN: DO NOT SHOW TITLE] MOTIVATION - EMOTION

- I don’t need a rapid diagnostic test before treating a patient to know I am giving them the right treatment
- I don’t always trust the quality and reliability of the rapid test kits supplied to my facility

[PN: DO NOT SHOW TITLE] ABILITY - CAPABILITY

- I have not received training on the procedures for administering malaria rapid diagnostic tests
- I am not familiar with the different malaria test types
- I am unsure when I should use a rapid diagnostic test instead of a microscopy diagnostic test
- It can be difficult to identify when an RDT is inaccurate or inconclusive

[PN: DO NOT SHOW TITLE] ABILITY - ROUTINES

- Administering rapid diagnostic tests is something I always do with all of my patients who are experiencing malaria symptoms
- I sometimes forget to test
- When the clinic is busy, it is difficult to test before diagnosis

[PN: DO NOT SHOW TITLE] PHYSICAL

- My practice has runout of rapid diagnostic tests on occasion in the past
- The rapid diagnostic tests are not stored properly at my facility which means their accuracy and performance are not maintained
- The instructions on rapid diagnostic tests are not always clear and easy to follow

[PN: DO NOT SHOW TITLE] SOCIAL - CULTURAL

- I do not discuss testing decisions with my colleagues

[PN: DO NOT SHOW TITLE] SOCIAL - NORMS

- Other healthcare professionals in my facility do not conduct rapid diagnostic tests before treatment
- Patients in my community do not expect to be tested for malaria when presenting with symptoms
- I try to follow suggested guidelines but it is not always possible

# Section D: Treatment practices

D1. Thinking about your patients who have had malaria, what types of treatment have you prescribed?

Please select all that apply.

1. Antimalarial pills
2. Antimalarial injection
3. Paracetamol
4. Iron tablets
5. Vitamin C
6. Traditional treatments (those that might be recommended by a traditional healer e.g herbal remedies)
7. Other

[PN: MULTICODE, RANDOMISE]

We would like to learn a little bit more about your past experiences with patients.

D2. [SHOW ONLY TO THOSE SELECTING ANTIMALARIAL PILLS AND/OR ANTIMALARIAL INJECTION [OPTION 1 AND/OR 2] AT D1] Please think of the last time you saw a patient who you gave antimalarial treatment to, from each of the following age groups. During your consultation, which of the following did you do?

[TOP, RESPONSES]

- Infants and neonates (<12 months) <5kg
- Infants and neonates (<12 months) 5kg or more
- Children (1-5 years)
- Children (6-12 years)
- Teenagers (13-17 years)
- Adults (>18 years)

[SIDE, STATEMENTS]

- I gave them an antimalarial treatment after they received a positive test result
- I gave them an antimalarial treatment after they received a negative test result
- I gave them an antimalarial treatment even though they did not do a test
- Other reason [FIX]

[PN: SINGLECODE, RANDOMISE]

D4. Thinking about your usual practice, to what extent do you agree or disagree with the following statement?

[PN: GRID, USE QUESTION LIBRARY FI011]

[PN: TOP, RESPONSES]

- Strongly Agree
- Agree
- Neither agree nor disagree
- Disagree
- Strongly Disagree
- Don’t know

[PN: DO NOT SHOW TITLE] MOTIVATION – OUTCOME EXPECTATIONS

- Prescribing treatment without a test result is unlikely to negatively impact my patient’s health
- Prescribing treatment to someone who may not have malaria can impact the efficacy of a treatment if they have to take it again in the future

[PN: DO NOT SHOW TITLE] MOTIVATION – SELF EFFICACY

I have enough experience to prescribe treatment without testing

[PN: DO NOT SHOW TITLE] MOTIVATION – EMOTION

- I worry about giving antimalarials without testing for malaria first
- It frustrates me when I see colleagues prescribing malaria treatment without testing first
- It frustrates me if a patient refuses to take a test

[PN: DO NOT SHOW TITLE] ABILITY – CAPABILITY

- I know all the different types of treatment for malaria
- I know the difference between each type of treatment for malaria

[PN: DO NOT SHOW TITLE] PROCESSING

- I tend to have a go-to treatment that I use when I know a patient has malaria

[PN: DO NOT SHOW TITLE] PHYSICAL

- I don’t always have enough antimalarials in stock to give to patients

[PN: DO NOT SHOW TITLE] SOCIAL

- The healthcare professionals I know typically prescribe oral antimalarial tablets over other types of treatment
- I often discuss malaria treatment options with my colleagues

# Section E: Encouraging Adherence

E1. Please think of the last time you saw a patient who was presenting symptoms of malaria, from each of the following age groups. During your consultation which of the following topics, if any, did you discuss with the patient/caregiver?

(Please select all that apply)

[TOP, RESPONSES]

- Infants and neonates (<12 months) <5kg
- Infants and neonates (<12 months) 5kg or more
- Children (1-5 years)
- Children (6-12 years)
- Teenagers (13-17 years)
- Adults (>18 years)

[SIDE, STATEMENTS]

- Dosage of the treatment
- Need for completion of treatment
- Other preventative measures e.g., using mosquito nets
- Techniques or strategies to remember to take all of the treatment
- Other (please specify) [OPEN END]

[SINGLE CODE]

E3. In the last 3 months, how many patients have you had a follow up appointment or discussion with after giving the treatment?

- All of my patients
- Most of my patients
- Some of my patients
- A few of my patients
- None of my patients

[SINGLE CODE]

E4. Whose responsibility is it to ensure patients take the full course of their treatment?

- It is solely the patient or their caregiver’s responsibility to ensure they take the full course of treatment
- It is both the patient or their caregiver’s and my responsibility as their healthcare provider to ensure they take the full course of treatment
- It is solely my responsibility to ensure patients take their full course of treatment

[SINGLE CODE]

E5. Thinking about your usual practice, to what extent do you agree or disagree with the following statements?

[PN: TOP, RESPONSES]

- Strongly Agree
- Agree
- Neither agree nor disagree
- Disagree
- Strongly Disagree
- Don’t know

[PN: DO NOT SHOW TITLE] MOTIVATION – OUTCOME EXPECTATIONS

- My discussion with the patient about their treatment makes a difference to whether they take it correctly

[PN: DO NOT SHOW TITLE] MOTIVATION – SELF EFFICACY

- I am confident in my ability to explain treatment dosage to my patients
- I am confident in my ability to explain reasons for completing treatment to my patients
- I find it easy to respond to most patient questions and concerns on taking or completing treatment

[PN: DO NOT SHOW TITLE] ABILITY – CAPABILITY

- I know the reasons for needing to complete the full course of treatment
- It can be difficult to explain the importance of adherence to treatment to patients
- I lack the time to have conversations with my patients about the importance of taking all of their treatment

[PN: DO NOT SHOW TITLE] PHYSICAL

- I use supporting materials (e.g., leaflets) to support my conversation on adherence to treatment with patients

[PN: DO NOT SHOW TITLE] SOCIAL

- The healthcare professionals I know have conversations about treatment dosage with patients
- The healthcare professionals I know have conversations about completing treatment with patients
- My colleagues and I discuss ways to encourage more patients to adhere to treatment
- It is solely my role to encourage patients to adhere to treatment
- I have seen my patients fully recover after giving them or prescribing treatment

# [SINGLE CODE]

# Section F: Pharmacovigilance Re-contact

F1. The sponsoring company of this research has an obligation to continuously monitor the safety of their products. Although rare, their Product Safety Department may want to further investigate any issues you may have raised in this survey. If we identify any adverse event/product complaint mentioned by you during this research, are you willing to be contacted by us to provide more information regarding the adverse event/product complaint?

(Please select one answer only)

- Yes, I am willing to be contacted to provide more information regarding the adverse event/product complaint
- No, I am not willing to be contacted to provide more information regarding the adverse event/product complaint

[PN: SINGLE CODE. SHOW IF CLOSED-END AE TRIGGERED WITHIN SURVEY OR IF OPEN-ENDED QUESTIONS IN QUESTIONNAIRE. FOR OPEN-ENDED QUESTIONS WHICH REQUIRE MANUAL REVIEW FOR AEs, USE F2 FOR OFF-LINE RE-CONTACT OF RESPONDENTS TO ASK FOR CONSENT TO PASS ON PERSONAL DATA TO CLIENT IN ALL MARKETS EXCEPT GERMANY. IN GERMANY, SHOW F3 IF APPLICABLE OR END SURVEY]

F2. Would you be willing to have your personal data passed on to the sponsoring company for the purpose of obtaining more information regarding any adverse event / product complaints mentioned in this research? Please note that if you were to consent to having your personal data passed on to the sponsoring company, Novartis, such personal data will be controlled and processed by their Product Safety Department. Their privacy notice that can be reviewed at this link LINK.

(Please select one answer only)

- Yes, I consent to my personal data being passed on to Novartis.
- No, I do not consent to my personal data being passed on to Novartis.

[PN: SINGLE CODE. SHOW ONLY IF CLOSED-END AE TRIGGERED WITHIN SURVEY AND ‘YES’ AT F1; DO NOT SHOW IN GERMANY. IF NO CLOSED-END AE TRIGGER BUT WITH OPEN-ENDED QUESTIONS WHICH REQUIRE MANUAL REVIEW FOR AEs, USE FOR OFF-LINE RE-CONTACT OF RESPONDENTS TO ASK FOR CONSENT TO PASS ON PERSONAL DATA TO CLIENT AS REQUIRED]

Ipsos

3 Thomas More Square, London, E1W 1YW, UK

Tel: + 44 20 3059 5000 Fax: +44 20 3059 4998

Internal / client use only

# BeSci Malaria Pathway

# Adults Questionnaire

**Online survey**

**30 minutes**

**Version 3**

**Sample**

|  | **Uganda** | **Ivory Coast** | **Nigeria** |
| --- | --- | --- | --- |
| **Adults** | **100** | **100** | **100** |

**Quotas**

| **SDistrict** | **Uganda** | **Ivory Coast** | **Nigeria** |
| --- | --- | --- | --- |
| **Sokoto** | **N/A** | **N/A** | **50** |
| **Taraba** | **N/A** | **N/A** | **50** |
| **Acholi** | **50** | **N/A** | **N/A** |
| **East-Central Busoga** | **50** | **N/A** | **N/A** |
| **Bas-Sassandra** | **N/A** | **50** | **N/A** |
| **La Vallee Du Bandaman** | **N/A** | **50** | **N/A** |

| **SDistrict** | **Uganda** | **Ivory Coast** | **Nigeria** |
| --- | --- | --- | --- |
| **Urban** | **30** | **30** | **30** |
| **Rural** | **70** | **70** | **70** |

| **Signed off for scripting / translation by:** | |
| --- | --- |
| **AD/D** |  |
| **PM** |  |

# Screener

S0. What is your interviewer ID number?

_____________________________________

[PN: OPEN NUMERIC]

SCountry. In which country does the respondent live?

(Please select one answer only)

- Uganda
- Nigeria
- Ivory Coast
- None of these

[PN: SINGLE CODE. CLOSE IF ‘NONE OF THESE’ SELECTED. DO NOT SHOW TO RESPONDENTS]

SIntro. This survey is being conducted by Ipsos, a research agency, on behalf of a pharmaceutical company. We are conducting research to understand the access and adherence to malaria treatment. Your data will be analysed only when aggregated with other respondents’ data and will not be identifiable to you. This analysis will be used to understand more about healthcare practices in your region and to inform potential healthcare interventions. We would like to ask you some questions on this topic. The survey will take approximately 30 minutes of your time.

This research is intended to understand your experiences with and in relation to malaria. Please note that your participation is voluntary and you may stop the survey at any time and we will destroy any data collected up until that point.

With your consent, your information will only be collected and used for social research and not given to anyone else**.** Any information you give will be treated with care to make sure nobody can see the responses you gave came from you. When we share information with the company who has asked for this research, we will remove any data which could identify you. We may need to send some of your personal data outside of your country of residence. We will make sure that it is always kept secure.

As a member of the Market Research Society (MRS), Ipsos is bound by the MRS Code of Conduct and all applicable laws protecting your personal data and responses. The study is conducted in compliance with local guidelines and codes of conduct. You can decide not to take part in the study at any time. For more information about your rights and how data will be used, please see our privacy notice, it is available here [PN: SCRIPTERS TO INSERT PRIVACY POLICY LINK].

If you mention any side effects with a medicine that our client makes or any product complaints, we must report this to the client. This is to help them learn more about the safety of their products. If this happens, we will need to collect details and report the side effects or product complaint.

You will be asked if you give your permission for us to pass your details to the company’s drug safety department, but you can also choose to remain anonymous if you wish. Even if you choose to give your name to the company related to an issue with your medicine, the rest of your interview will remain confidential and anonymous.

Are you happy to participate in this research as described?

(Please select one answer only)

- Yes, I wish to continue
- No, I do not wish to continue

[PN: SINGLE CODE. CLOSE IF ‘NO’ SELECTED]

SSpecial. Do you consent to providing information relating to malaria as described above?

- Yes, I consent to providing information as described above.
- No, I do not consent to providing information as described above.

[PN: SINGLE CODE. CLOSE IF ‘NO’ SELECTED]

SDistrict. In which district do you spend most of your time?

(Please select one answer only)

**Recruit to soft quotas**

**[PN: Show if SCountry = NIGERIA]**

- Sokoto
- Taraba
- Other

**[PN: Show if SCountry = UGANDA]**

- Acholi
- East-Central Busoga
- Other

**[PN: Show if SCountry = IVORY COAST]**

- Bas-Sassandra
- La Vallee Du Bandaman
- Other

**[PN: SINGLE CODE. CLOSE IF OTHER SELECTED]**

SArea. Which of the following best describes the area where you live?

(Please select one answer only)

- Urban
- Rural
- Not sure **[ASSIGN RANDOMLY TO URBAN/RURAL SOFT QUOTA]**

[PN: RANDOMISE, RECRUIT 30% URBAN AND 70% RURAL]

SAge. What was your age at your last birthday?

(Please type in respondent’s answer)

- Range 0-100 years

[PN: OPEN NUMERIC. 0-100. CLOSE IF <18]

**SMalaria.** How many times, if at all, have you had malaria, in the last 5 years? If unsure, please provide an estimate.

_____ times

- I have not had malaria
- Don’t know **[EXCLUSIVE]**

**[PN: OPEN NUMERIC, ALLOW 1-50, CLOSE IF I HAVE NOT HAD MALARIA OR DON’T KNOW]**

**SResearch.** Have you taken part in any market or social research about malaria in the last month?

- Yes
- No

**[PN: SINGLE CODE, CLOSE IF YES SELECTED]**

# Section A: Demographics

**A1.** Which of the following best describes your gender?

- Man
- Woman
- In another way
- Prefer not to say

**[PN: SINGLE CODE]**

A2. What best describes your employment status?

(Please select one answer only)

- Full time employment (30+ hours per week)
- Part time employment (8-29 hours per week)
- Student
- Unemployed
- Retired
- Other [PN: EXCLUSIVE]
- Prefer not to say [PN: EXCLUSIVE]

[PN: SINGLE CODE]

A3. [UGANDA AND NIGERIA ONLY] What is your usual **annual** household income taken home (e.g after tax)?

[IVORY COAST ONLY] What is your usual **monthly** household income taken home (e.g after tax)?

(Please select one answer only)

[PN: SHOW IN UGANDA ONLY]

- Below UGX 500,000
- UGX 500,001 - UGX 1,000,000
- UGX 1,000,001 - UGX 1,500,000
- UGX 1,500,001 - UGX 2,000,000
- Above UGX 2,000,001
- Prefer not to say
- Don’t know

[PN: SHOW IN IVORY COAST ONLY]

- Rien/aucun revenu
- Less than 20 000 FCFA
- 20 000 - 39 999 FCFA
- 40 000 - 59 999 FCFA
- 60 000 - 99 999 FCFA
- 100 000 - 299 999 FCFA
- 300 000 - 449 999 FCFA
- 450 000 -799 999 FCFA
- 800000 FCFA and above
- Prefer not to say
- Don’t know

[PN: SHOW IN NIGERIA ONLY]

- Less than N360, 000
- N360,000 - N1,500,000
- N1,500,001 - N3,000,000
- N3,000,001 - N6,000,000
- More than N6,000,001
- Prefer not to say
- Don’t know

[PN: SINGLE CODE]

A4. Which of the following best describes your highest level of education?

- No formal education
- Completed primary education
- Completed secondary education
- Completed college or technical school
- Completed undergraduate degree
- Completed postgraduate degree (Master's, PhD, etc.)
- Prefer not to answer

[PN: SINGLE CODE]

A5. Which of the following best describes your marital status?

- Single, never married
- Married
- Living with partner
- Separated
- Divorced
- Widowed
- Prefer not to answer

[PN: SINGLE CODE]

**A6.** Do any of the following roles apply to you?

Please select as many roles that apply.

- Religious Leader
- Local Council Member
- Government Official
- Other **– OPEN END**
- None of these roles apply to me **[EXCLUSIVE]**

**[PN: MULTICODE]**

# Section B: Malaria

**B1.** How recently did you last have malaria?

- Less than a month ago
- 1-5 months ago
- 6-12 months ago
- More than 12 months ago

**[PN: SINGLE CODE]**

**B2.** Thinking about the last time you had malaria, which of the following did you do after realising your symptoms might indicate malaria?

(Please select all that apply)

- I waited a few days for the symptoms to go themselves
- I went to a healthcare facility to get medication (including antimalarials)
- I went to a healthcare facility to get a test to confirm malaria
- I went to a traditional healer for advice
- I went to a traditional healer for treatment
- I went to a community leader for advice
- I went to a community leader for treatment
- I treated my symptoms at home with paracetamol I had already
- I treated my symptoms at home with antimalarials that I had already
- I treated my symptoms at home with traditional medicine
- Other **- FIXED**
- Don’t know **– FIXED, EXCLUSIVE**

**[PN: MULTICODE, RANDOMISE]**

B3. [ASK MORE THAN 1 SELECTED AT B2] Please rank the following actions in the order you did them.

- [PIPE IN ANSWERS FROM B2]

[PN: MULTICODE, RANDOMISE]

B4. [IF WENT TO A HEALTHCARE FACILITY AT B2] When you last had malaria, which of the following healthcare facilities did you visit?

- Pharmacy
- Public clinic
- Private clinic
- Public hospital
- Private hospital
- I did not visit any of the facilities above

[PN: MULTICODE, RANDOMISE]

B5. For the healthcare facility that you visited, how long did it take you to get to there by your usual means of travel?

[PN: TOP, RESPONSES]

- [PIPE IN ANSWERS FROM B4]

[PN: SIDE, STATEMENTS]

- <30 minutes
- 30 minutes – 1 hour
- 1-2 hours
- 3-5 hours
- >5 hours
- Don’t know

[PN: SINGLE CODE]

B6. Thinking about the last time you had malaria, which of the following best describes your experience?

(Please select one answer only)

- My **healthcare professional offered** me a test that confirmed my diagnosis
- I **asked a healthcare professional** for a test to confirm my diagnosis
- My diagnosis **was not** confirmed by a test

[PN: SINGLE CODE]

**B7.** How often, if at all, do you worry about the possibility of catching malaria?

- Almost always
- Often
- Sometimes
- Never or rarely
- Don’t know

**[PN: SINGLE CODE]**

**B8.** How often, if at all, have you done each of the following in the last 6 months to prevent malaria?

[PN: TOP, RESPONSES]

- Always
- Sometimes
- Rarely
- Never
- Don’t know

[PN: SIDE, STATEMENTS]

- I use mosquito nets
- I use insect repellent
- I regularly clean my surroundings to prevent mosquito breeding
- I use air conditioning or fans
- I make sure I do not come into contact with stagnant water
- I wear long sleeves and trousers
- Other (please specify) **– OPEN END**
- None of the above **– EXCLUSIVE**
- Don’t know **– EXCLUSIVE**

[PN: SINGLE CODE PER STATEMENT]

PN: FOR OTHER PLEASE SPECIFY SHOW THE FOLLOWING TEXT: You checked 'other' in the previous question. Please clarify and share what other actions you did to prevent malaria.

# Section C: Access

**C1.** Please indicate whether the following symptoms may or do not indicate malaria.

**[PN: TOP, RESPONSES]**

- May indicate malaria
- Does not indicate malaria
- Don’t know

**[PN: SIDE, STATEMENTS]**

- High fever
- Shaking chills
- Profuse sweating
- Headache
- Abdominal pain
- Muscle pain
- Nausea
- Vomiting
- Diarrhoea
- Rash
- Cough
- Change in skin tone
- Loss of sense of taste
- Loss of sense of smell
- Other (please specify) **[OPEN TEXT]**

[PN: MULTICODE, RANDOMISE STATEMENTS]

**C2. [ONLY IF MORE THAN 3 DO INDICATE MALARIA AT C1]** Of those that you said may indicate malaria, which three symptoms are most likely to indicate malaria to you?

- **[PIPE IN RESPONSES FROM C1 THAT DO INDICATE MALARIA]**
- Other (please specify) **[OPEN TEXT]**

[PN: MULTICODE, MUST SELECT 3, RANDOMISE STATEMENTS]

**SEEKING CARE**

**C3.** There are several reasons people **may or may not seek care**, advice or treatment when they first present symptoms that might suggest malaria. Please tell me to what extent you agree or disagree with the following statements.

[PN: GRID, USE QUESTION LIBRARY FI011]

[PN: TOP, RESPONSES]

- Strongly Agree
- Agree
- Neither agree nor disagree
- Disagree
- Strongly Disagree
- Don’t know

[PN: DO NOT SHOW TITLE] MOTIVATION – EMOTION

- I feel scared if I get symptoms that could indicate malaria

[PN: DO NOT SHOW TITLE] MOTIVATION – OUTCOME EXPECTATIONS

- Paracetamol gets rid of my malaria symptoms, just as well as a different treatment I could get from a healthcare facility
- Seeking care from a healthcare professional will help me recover from malaria
- It is easy for me to get professional help for my malaria symptoms
- I am confident that a healthcare professional can give me the right advice on what to do to treat my malaria

**[PN: RANDOMISE WITHIN SUBGROUP]**

**[PN: DO NOT SHOW TITLE]** **ABILITY**

- I can easily identify early symptoms of malaria in myself
- I can easily identify early symptoms of malaria in others
- It can be difficult to distinguish the symptoms of malaria from other conditions
- I know where to go for healthcare advice when I am experiencing malaria symptoms
- It is important to seek care for my malaria symptoms early to avoid complications in the future

**[PN: RANDOMISE WITHIN SUBGROUP]**

**[PN: DO NOT SHOW TITLE]** **PHYSICAL**

- The healthcare facility I would like to go to is too far away for me to consider it
- My work and/or childcare responsibilities make it difficult for me to go to a healthcare facility quickly

**[PN: DO NOT SHOW TITLE]** **SOCIAL**

- Other people around me do not prioritize going to the clinic for advice when they experience malaria symptoms
- In my community, it is more important to seek care for other diseases instead of malaria
- In my community, seeking health advice from a traditional healer is preferred instead of going to the clinic

**[PN: RANDOMISE WITHIN SUBGROUP AND ORDER OF SUBGROUPS]**

C4. Which of the following did you do the last time a friend or someone in your community experienced symptoms of malaria.

(Please select as many that apply)

- I told them to visit a traditional healer
- I told them to use a traditional medicine
- I told them to visit a medical professional at the hospital or clinic
- I told them to visit a medical professional at the pharmacy
- I told them to take paracetamol and see if they felt better
- I did not say anything [EXCLUSIVE, FIXED]

[PN: MULTICODE, RANDOMISE]

**WHERE TO SEEK CARE**

**C5.** Please tell me to what extent you agree or disagree with the following statements when it comes to where to seek care when you might have malaria.

**[PN: TOP, RESPONSES]**

- Strongly agree
- Somewhat agree
- Neither agree nor disagree
- Somewhat disagree
- Strongly disagree
- Don’t know

**[PN: SIDE, STATEMENTS]**

**[PN: DO NOT SHOW TITLE] MOTIVATION**

- I believe the pharmacist can treat my malaria symptoms better than a healthcare professional at a hospital or clinic
- I am confident that going to a qualified healthcare professional for advice is the best option when I am experiencing malaria symptoms
- I believe it's worth the time and cost to travel to the hospital or clinic to get tested for malaria before being treated
- I believe it's worth the time and cost to travel to the hospital or clinic to get treated for malaria
- I prefer going to the pharmacy for malaria because I can get the medication I need right away without a prescription
- I trust the pharmacist's advice on which malaria medication to take, so I don't feel the need to see a doctor first

**[PN: DO NOT SHOW TITLE] ABILITY**

- I'm used to describing my malaria symptoms to the pharmacist and purchasing treatment medication without getting tested first

**[PN: DO NOT SHOW TITLE] PHYSICAL**

- It's quicker for me to visit a pharmacy than a hospital or clinic when I am experiencing malaria symptoms
- It's cheaper for me to visit a pharmacy than a hospital or clinic when I am experiencing malaria symptoms
- There is an accessible hospital/clinic close to my home that I can go to if I am experiencing malaria symptoms
- There are transport options available for me to go to the hospital or clinic if I develop malaria symptoms
- Pharmacies are much closer to my home than the nearest hospital or clinic
- I can get malaria medication at a lower cost from the pharmacy compared to the consultation and prescription fees at a hospital or clinic
- I set aside money in case I need to cover the cost of travel or treatment if I do get sick

**[PN: DO NOT SHOW TITLE] SOCIAL**

- Most people in my community self-treat at home rather than going to a hospital or clinic for malaria treatment
- I prefer to seek care from a community leader first, before seeking care from a trained healthcare professional
- Community leaders encourage us to seek professional medical care immediately if we suspect malaria rather than waiting

**[PN: DO NOT SHOW TITLE] PROCESSING**

- When I think I might have malaria symptoms, my first instinct is to go to a healthcare professional for diagnosis
- I have noticed information on the importance of seeking professional care at a hospital or clinic when I think I have malaria

**PN: RANDOMISE WITHIN SUBGROUP AND ORDER OF SUBGROUPS**

**GETTING TESTED**

**C6.** We’d like to know a bit more about your attitudes and beliefs when it comes to getting tested for malaria to confirm you have the disease. Please tell me to what extent you agree or disagree with the following statements.

**[PN: TOP, RESPONSES]**

- Strongly agree
- Somewhat agree
- Neither agree nor disagree
- Somewhat disagree
- Strongly disagree
- Don’t know

**[PN: SIDE, STATEMENTS]**

**[PN: DO NOT SHOW TITLE] MOTIVATION**

- I believe getting tested is the only way to confirm if I really have malaria
- I am confident that getting tested will help me receive the right treatment for malaria
- I believe that getting tested for malaria will save me time in the future
- I believe that getting tested for malaria will save me money in the future

**[PN: RANDOMISE CODES WITHIN SUBGROUP]**

**[PN: DO NOT SHOW TITLE] ABILITY**

- I know where to go to get a malaria test
- It is important to get tested to confirm the diagnosis

**[PN: RANDOMISE CODES WITHIN SUBGROUP]**

**[PN: DO NOT SHOW TITLE] PHYSICAL**

- I can afford the cost of a malaria test
- Malaria testing is easy to get at a healthcare facility near me
- The process of getting tested for malaria is quick

**[PN: RANDOMISE CODES WITHIN SUBGROUP AND ORDER OF SUBGROUPS]**

# Section D: Adherence

The next few questions will focus on treatments for malaria.

D1. Thinking about the last time you had malaria, which of the following treatments did you take?

Please select all that apply.

- Traditional treatments
- Antimalarial pills prescribed by a doctor
- Antimalarial pills I was given by a friend / family member
- Antimalarial pills I already had in my possession
- Antimalarials pills I bought from a shop
- Antimalarial injection
- Paracetamol
- Iron tablets
- Other

[PN: SINGLECODE, RANDOMISE STATEMENTS]

D2. Thinking about the last time you took antimalarials, which of the following best describes how you took your treatment?

- I took all of my antimalarials
- I took some of my antimalarials
- I did not take any antimalarials
- Don’t know

[PN: SINGLECODE, RANDOMISE STATEMENTS]

D3. [IF D2 = I TOOK SOME OF MY ANTIMALARIALS] Thinking about the last time you had malaria, which of the following best describes why you didn’t take all your medication?

(Please select all that apply)

- I forgot to take it
- I stopped taking it when my symptoms disappeared
- I stopped taking it because it did not help relieve my symptoms
- I stopped taking it because I didn’t like the taste
- I stopped taking it because it made me feel unwell
- I wanted to save some treatment for next time
- Don’t know - **EXCLUSIVE**

[PN: MULTICODE, RANDOMISE STATEMENTS]

D4. There are several reasons people may or may not take all their malaria treatment. Please state the extent to which you agree or disagree with the following statements. When answering these questions, please think about antimalarial pills you may take to treat your malaria.

[PN: GRID, USE QUESTION LIBRARY FI011]

[PN: TOP, RESPONSES]

- Strongly agree
- Agree
- Neither agree nor disagree
- Disagree
- Strongly disagree
- Don’t know

[PN: SIDE, STATEMENTS]

[PN: DO NOT SHOW TITLE] MOTIVATION – OUTCOME EXPECTATIONS

- Once my malaria symptoms are gone, there is nothing left for the treatment to do

[PN: DO NOT SHOW TITLE] ABILITY – KNOWLEDGE

- It is fine to stop treatment when my symptoms are gone, because it means that the disease is gone
- I know the reasons for needing to take the full course of treatment
- I know exactly how often and when I should take my antimalarial treatment

[PN: DO NOT SHOW TITLE] ABILITY – ROUTINE

- It can be difficult to remember to take all of the doses of antimalarial treatment

[PN: DO NOT SHOW TITLE] PHYSICAL – ENVIRONMENTAL

- The taste of antimalarial pills makes them difficult to take
- Malaria treatment is expensive
- Stopping malaria treatment early means that I can save money treatment for next time, which saves me money
- Stopping malaria treatment early means that I can don’t have to see a healthcare professional next time I have malaria

[PN: DO NOT SHOW TITLE] SOCIAL – SOCIAL

- People I know have stopped taking the antimalarial treatment early and still recovered
- People I know believe one should take all of their antimalarial treatment, even when their symptoms disappear

**[PN: MULTICODE, RANDOMISE STATEMENTS AND ORDER OF SUBGROUPS]**

# Section F: Pharmacovigilance contact

F1. The sponsoring company of this research has an obligation to continuously monitor the safety of their products. Although rare, their Product Safety Department may want to further investigate any issues you may have raised in this survey. If we identify any adverse event/product complaint mentioned by you during this research, are you willing to be contacted by us to provide more information regarding the adverse event/product complaint?

(Please select one answer only)

- Yes, I am willing to be contacted to provide more information regarding the adverse event/product complaint
- No, I am not willing to be contacted to provide more information regarding the adverse event/product complaint

[PN: SINGLE CODE. SHOW IF CLOSED-END AE TRIGGERED WITHIN SURVEY OR IF OPEN-ENDED QUESTIONS IN QUESTIONNAIRE. FOR OPEN-ENDED QUESTIONS WHICH REQUIRE MANUAL REVIEW FOR AEs, USE F2 FOR OFF-LINE RE-CONTACT OF RESPONDENTS TO ASK FOR CONSENT TO PASS ON PERSONAL DATA TO CLIENT IN ALL MARKETS EXCEPT GERMANY. IN GERMANY, SHOW F3 IF APPLICABLE OR END SURVEY]

F2. Would you be willing to have your personal data passed on to the sponsoring company for the purpose of obtaining more information regarding any adverse event / product complaints mentioned in this research? Please note that if you were to consent to having your personal data passed on to the sponsoring company, Novartis, such personal data will be controlled and processed by their Product Safety Department. Their privacy notice that can be reviewed at this LINK.

(Please select one answer only)

- Yes, I consent to my personal data being passed on to Novartis.
- No, I do not consent to my personal data being passed on to Novartis.

[PN: SINGLE CODE. SHOW ONLY IF CLOSED-END AE TRIGGERED WITHIN SURVEY AND ‘YES’ AT F1; DO NOT SHOW IN GERMANY. IF NO CLOSED-END AE TRIGGER BUT WITH OPEN-ENDED QUESTIONS WHICH REQUIRE MANUAL REVIEW FOR AEs, USE FOR OFF-LINE RE-CONTACT OF RESPONDENTS TO ASK FOR CONSENT TO PASS ON PERSONAL DATA TO CLIENT AS REQUIRED]
