# Supplementary material for: Understanding barriers to effective malaria diagnosis and treatment: A mixed-methods study in Uganda, Nigeria, and Ivory Coast
Source: PLOS Glob Public Health. 2026 Aug 17;6(8):e0006921. doi: 10.1371/journal.pgph.0006921 (PMC13480639; doi:10.1371/journal.pgph.0006921)
Supplement: Supplementary file 3 [file pgph.0006921.s003.docx]

**S1 Table: Testing, by HCP type**

|  | **Total**  **n= 532** | **Nurse n=196** | **Community Health Worker n=190** | **Pharmacist n=146** |
| --- | --- | --- | --- | --- |
| **Attitudes toward testing before treating**^1^ | | | | |
| It is important to act quickly if I suspect a patient has malaria | 455 (86%) | 159 (81%)* | 170 (89%) | 126 (86%) |
| Administering RDTs is something I always do with all my patients who are experiencing malaria symptoms | 358 (67%) | 131 (67%) | 140 (74%)* | 87 (60%) |
| If I am unable to confirm my patient’s diagnosis with a test, they might end up spending more on their treatment | 349 (66%) | 124 (63%) | 135 (71%) | 90 (62%) |
| Diagnostic tests conducted in the lab are more reliable than RDTs | 345 (65%) | 121 (62%) | 134 (71%) | 90 (62%) |
| If I am unable to confirm my patient’s diagnosis with a test, their health might be negatively impacted | 323 (61%) | 109 (56%) | 127 (67%) | 87 (60%) |
| If I am unable to confirm my patient’s diagnosis with a test, it is more difficult for me know what treatment to give them | 308 (58%) | 107 (55%) | 119 (63%) | 82 (56%) |
| Patients in my community do not expect to be tested for malaria when presenting with symptoms | 188 (35%) | 67 (34%) | 61 (32%) | 60 (41%) |
| I try to follow suggested guidelines, but it is not always possible | 151 (28%) | 48 (24%) | 59 (31%) | 44 (30%) |
| My practice has run out of RDTs on occasion in the past | 150 (28%) | 57 (29%) | 60 (32%) | 33 (23%) |
| Other HCPs in my facility do not conduct RDTs before prescribing treatment | 120 (23%) | 32 (16%)* | 31 (16%)* | 57 (39%)*** |
| I don’t need an RDT before treating a patient to know I am giving them the right treatment | 108 (20%) | 38 (19%) | 27 (14%)* | 43 (29%)** |
| I do not discuss testing decisions with my colleagues | 108 (20%) | 47 (24%) | 37 (19%) | 24 (16%) |
| I need some support in interpreting the results of malaria RDTs | 99 (19%) | 31 (16%) | 35 (18%) | 33 (23%) |
| I am not familiar with the different malaria test types | 91 (17%) | 30 (15%) | 40 (21%) | 21 (14%) |
| It can be difficult to identify when an RDT is inaccurate or inconclusive | 91 (17%) | 35 (18%) | 31 (16%) | 25 (17%) |
| RDTs for malaria are unreliable | 85 (16%) | 35 (18%) | 20 (14%) | 28 (13%) |
| I don’t always trust the quality and reliability of the RDT kits supplied to my facility | 84 (16%) | 30 (15%) | 23 (12%) | 31 (21%) |
| When the clinic is busy, it is difficult to test before diagnosis | 83 (16%) | 32 (16%) | 26 (14%) | 25 (17%) |
| The RDTs are not stored properly at my facility which means their accuracy and performance are not maintained | 79 (15%) | 29 (15%) | 30 (16%) | 20 (14%) |
| The instructions on RDTs are not always clear and easy to follow | 76 (14%) | 25 (13%) | 26 (14%) | 25 (17%) |
| I find it challenging to administer malaria RDTs to patients | 53 (10%) | 17 (9%) | 18 (9%) | 18 (12%) |
| I have not received training on the procedures for administering malaria RDTs | 47 (9%) | 18 (9%) | 15 (8%) | 14 (10%) |
| I sometimes forget to test | 37 (7%) | 9 (5%) | 11 (6%) | 17 (12%) |
| **Perceived responsibility to ensure patients receive a diagnostic test** | | | | |
| It is my responsibility to ensure patients receive a test to diagnose malaria | 145 (27%) | 67 (34%)* | 57 (30%) | 21 (14%)*** |
| It is both the patient/caregiver and my responsibility as a HCP to ensure the patient receives a test to diagnose malaria | 333 (63%) | 115 (59%) | 116 (61%) | 102 (70%) |
| It is solely the patient/caregiver’s responsibility to ensure the patient receives a test to diagnose malaria | 54 (10%) | 14 (7%) | 17 (9%) | 23 (16%)* |
|  |  |  |  |  |
| *Base: all HCPs who prescribe antimalarials* | **Total n=491** | **Nurse**  **n= 187** | **Community Health Worker n=166** | **Pharmacist n=138** |
| **Most recent experience testing and treating a patient** | | | | |
| ***Infants and neonates <5 kg*** | | | | |
| I gave them antimalarial treatment after they received a positive test result | 293 (60%) | 123 (66%) | 85 (51%)* | 85 (62%) |
| I gave them antimalarial treatment after they received a negative test result | 90 (18%) | 32 (17%) | 30 (18%) | 28 (20%) |
| I gave them antimalarial treatment even though they did not do a test | 80 (16%) | 23 (12%) | 35 (21%) | 22 (16%) |
| Other | 28 (6%) | 9 (5%) | 16 (10%)* | 3 (2%)* |
| ***Infants and neonates 5 kg or more*** | | | | |
| I gave them antimalarial treatment after they received a positive test result | 303 (62%) | 119 (64%) | 96 (58%) | 88 (64%) |
| I gave them antimalarial treatment after they received a negative test result | 83 (17%) | 35 (19%) | 27 (16%) | 21 (15%) |
| I gave them antimalarial treatment even though they did not do a test | 89 (18%) | 25 (13%) | 38 (23%) | 26 (19%) |
| Other | 16 (3%) | 8 (4%) | 5 (3%) | 3 (2%) |
| ***Children 1-5 years*** | | | | |
| I gave them antimalarial treatment after they received a positive test result | 318 (65%) | 127 (68%) | 109 (66%) | 82 (59%) |
| I gave them antimalarial treatment after they received a negative test result | 63 (13%) | 21 (11%) | 20 (12%) | 22 (16%) |
| I gave them antimalarial treatment even though they did not do a test | 74 (15%) | 21 (11%) | 28 (17%) | 25 (18%) |
| Other | 36 (7%) | 18 (10%) | 9 (5%) | 9 (7%) |
| ***Children 6-12 years*** | | | | |
| I gave them antimalarial treatment after they received a positive test result | 299 (61%) | 119 (64%) | 97 (58%) | 83 (60%) |
| I gave them antimalarial treatment after they received a negative test result | 59 (12%) | 22 (12%) | 19 (11%) | 18 (13%) |
| I gave them antimalarial treatment even though they did not do a test | 91 (19%) | 31 (17%) | 33 (20%) | 27 (20%) |
| Other | 42 (9%) | 15 (8%) | 17 (10%) | 10 (7%) |
| ***Teenagers 13-17 years*** | | | | |
| I gave them antimalarial treatment after they received a positive test result | 288 (59%) | 119 (64%) | 95 (57%) | 74 (54%) |
| I gave them antimalarial treatment after they received a negative test result | 74 (15%) | 31 (17%) | 21 (13%) | 22 (16%) |
| I gave them antimalarial treatment even though they did not do a test | 95 (19%) | 30 (16%) | 29 (17%) | 36 (26%)* |
| Other | 34 (7%) | 7 (4%) | 21 (13%)** | 6 (4%) |
| ***Adults >18 years*** | | | | |
| I gave them antimalarial treatment after they received a positive test result | 273 (56%) | 113 (60%) | 90 (54%) | 70 (51%) |
| I gave them antimalarial treatment after they received a negative test result | 66 (13%) | 25 (13%) | 20 (12%) | 21 (15%) |
| I gave them antimalarial treatment even though they did not do a test | 114 (23%) | 38 (20%) | 36 (22%) | 40 (29%) |
| Other | 38 (8%) | 11 (6%) | 20 (12%) | 7 (5%) |

^1 'Top 2 Box' agreement shown, e.g. % of respondents selecting 1-2 on a 1-5 scale^

***** p <.05 **p <.01 ***p <.001
