# Supplementary material for: Understanding barriers to effective malaria diagnosis and treatment: A mixed-methods study in Uganda, Nigeria, and Ivory Coast
Source: PLOS Glob Public Health. 2026 Aug 17;6(8):e0006921. doi: 10.1371/journal.pgph.0006921 (PMC13480639; doi:10.1371/journal.pgph.0006921)
Supplement: Supplementary file 4 [file pgph.0006921.s004.docx]

**S2 Table: Adherence, by HCP type**

|  | **Total**  **n= 532** | **Nurse**  **n=196** | **Community Health Worker n=190** | **Pharmacist n=146** |
| --- | --- | --- | --- | --- |
| **Perceived responsibility of patient in adherence to treatment** | | | | |
| It is solely the patient/their caregiver’s responsibility to ensure they take the full course of treatment | 97 (18%) | 35 (18%) | 24 (13%) | 38 (26%)* |
| It is both the patient/their caregiver’s and my responsibility as their HCP to ensure they take the full course of treatment | 400 (75%) | 152 (78%) | 146 (77%) | 102 (70%) |
| It is solely my responsibility to ensure patients take their full course of treatment | 35 (7%) | 9 (5%) | 20 (11%)* | 6 (4%) |
| **Attitudes toward discussing adherence with patients**^1^ | | | | |
| I know the reasons for needing to complete the full course of treatment | 502 (94%) | 185 (94%) | 183 (96%) | 134 (92%) |
| I am confident in my ability to explain reasons for completing treatment to my patients | 501 (94%) | 202 (95%) | 182 (93%) | 179 (94%) |
| I have seen my patients fully recover after giving them or prescribing treatment | 496 (93%) | 182 (93%) | 184 (97%)* | 130 (89%) |
| I am confident in my ability to explain treatment dosage to my patients | 494 (93%) | 181 (92%) | 178 (94%) | 135 (92%) |
| My discussion with the patient about their treatment makes a difference to whether they take it correctly | 482 (91%) | 177 (90%) | 175 (92%) | 130 (89%) |
| My colleagues and I discuss ways to encourage more patients to adhere to treatment | 473 (89%) | 171 (87%) | 171 (90%) | 131 (90%) |
| The HCPs I know have conversations about treatment dosage with patients | 470 (88%) | 170 (87%) | 172 (91%) | 128 (88%) |
| The HCPs I know have conversations about completing treatment with patients | 465 (87%) | 171 (87%) | 166 (87%) | 128 (88%) |
| I find it easy to respond to most patient questions and concerns on taking or completing treatment | 463 (87%) | 189 (86%) | 173 (91%) | 121 (83%) |
| It is solely my role to encourage patients to adhere to treatment | 440 (83%) | 154 (79%) | 162 (85%) | 124 (85%) |
| I use supporting materials (e.g., leaflets) to support my conversations on adherence to treatment with patients | 339 (64%) | 129 (66%) | 122 (64%) | 88 (60%) |
| It can be difficult to explain the importance of adherence to treatment to patients | 164 (31%) | 70 (36%) | 64 (34%) | 30 (21%)** |
| I lack the time to have conversations with my patients about the importance of taking all of their treatment | 95 (18%) | 44 (22%) | 32 (17%) | 19 (13%) |

^1 'Top 2 Box' agreement shown, e.g. % of respondents selecting 1-2 on a 1-5 scale^

***** p <.05 **p <.01 ***p <.001
