# Supplementary material for: Understanding barriers to effective malaria diagnosis and treatment: A mixed-methods study in Uganda, Nigeria, and Ivory Coast
Source: PLOS Glob Public Health. 2026 Aug 17;6(8):e0006921. doi: 10.1371/journal.pgph.0006921 (PMC13480639; doi:10.1371/journal.pgph.0006921)
Supplement: Supplementary file 5 [file pgph.0006921.s005.docx]

**S3 Table: Final Qualitative Sample and Qualitative Sample Quotas**

| **Final Qualitative Sample and (minimum quotas): Nurse, Community Health Worker, Pharmacist** | | | | | | | | | |
| --- | --- | --- | --- | --- | --- | --- | --- | --- | --- |
|  | **Nigeria** | | | **Ivory Coast** | | | **Uganda** | | |
|  | **Nurses** | **Community health workers** | **Pharmacists** | **Nurses** | **Community health workers** | **Pharmacists** | **Nurses** | **Community health workers** | **Pharmacists** |
| S1: [NIGERIA] Sokoto | **2 (2)** | **2 (2)** | **2 (2)** | - | - | - | - | - | - |
| S1: [NIGERIA] Taraba | **2 (2)** | **2 (2)** | **2 (2)** | - | - | - | - | - | - |
| S1: [UGANDA] Acholi | - | - | - | - | - | - | **2 (2)** | **2 (2)** | **2 (2)** |
| S1: [UGANDA] East-Central Busoga | - | - | - | - | - | - | **2 (2)** | **2 (2)** | **2 (2)** |
| S1: [IVORY COAST] Bas-Sassandra | - | - | - | **2 (2)** | **2 (2)** | **2 (2)** | - | - | - |
| S1: [IVORY COAST] La Vallee Du Bandaman | - | - | - | **2 (2)** | **2 (2)** | **2 (2)** | - | - | - |
| Total: | 4 | 4 | 4 | 4 | 4 | 4 | 4 | 4 | 4 |
|  | 12 (12) | | | 12 (12) | | | 12 (12) | | |
| **The following quotas do not sum to total sample discretely because they are intersectional** | | | | | | | | | |
| S2 Quota: Urban | 1 (2) | 1 (2) | 2 (-) | 2 (2) | 1 (2) | 4 (-) | 1 (2) | 2 (2) | 3 (-) |
| S2 Quota: Rural | 3 (4) | 3 (4) | 2 (-) | 2 (4) | 3 (4) | 0 (-) | 1 (4) | 1 (4) | 1 (-) |

| **Final Qualitative Sample and (minimum quotas): Adult and Caregiver** | | | | | | |
| --- | --- | --- | --- | --- | --- | --- |
|  | **Nigeria** | | **Ivory Coast** | | **Uganda** | |
|  | **Adults** | **Caregivers** | **Adults** | **Caregivers** | **Adults** | **Caregivers** |
| S1: [NIGERIA] Sokoto | **2 (3)** | **7 (6)** | **-** | **-** | **-** | **-** |
| S1: [NIGERIA] Taraba | **2 (3)** | **7 (6)** | **-** | **-** | **-** | **-** |
| S1: [UGANDA] Acholi | **-** | **-** | **-** | **-** | **2 (3)** | **7 (6)** |
| S1: [UGANDA] East-Central Busoga | **-** | **-** | **-** | **-** | **2 (3)** | **7 (6)** |
| S1: [IVORY COAST] Bas-Sassandra | **-** | **-** | **3 (3)** | **6 (6)** | **-** | **-** |
| S1: [IVORY COAST] La Vallee Du Bandaman | **-** | **-** | **3 (3)** | **6 (6)** | **-** | **-** |
| Total: | **4** | **14** | **6** | **12** | **4** | **14** |
|  | **18** | | **18** | | **18** | |
| **The following quotas do not sum to total sample discretely because they are intersectional** | | | | | | |
| S2 Quota: Urban | **2 (2)** | **4 (2)** | **2 (2)** | **2 (2)** | **2 (2)** | **4 (2)** |
| S2 Quota: Rural | **2 (4)** | **10 (4)** | **4 (4)** | **10 (4)** | **4 (4)** | **8 (4)** |
| S6 Quota: Parent/Primary guardian | **12 (8)** | | **8 (8)** | | **7 (8)** | |
| S6 Quota: Grandparent | **2 (2)** | | **4 (2)** | | **4 (2)** | |
| S6 Quota: Aunt / Uncle | **4 (2)** | | **4 (2)** | | **5 (2)** | |
| S6 Quota: Community leaders | **6 (4)** | | **2 (4)** | | **4 (4)** | |
