# Supplementary material for: Understanding barriers to effective malaria diagnosis and treatment: A mixed-methods study in Uganda, Nigeria, and Ivory Coast
Source: PLOS Glob Public Health. 2026 Aug 17;6(8):e0006921. doi: 10.1371/journal.pgph.0006921 (PMC13480639; doi:10.1371/journal.pgph.0006921)
Supplement: Supplementary file 6 [file pgph.0006921.s006.docx]

**S4 Table: Final Quantitative Sample**

| **Final Quantitative Sample** | | | | |
| --- | --- | --- | --- | --- |
| **Adults + Caregivers** | | | | |
|  | Total  n=769 | Uganda  n=250 | Nigeria  n=252 | Ivory Coast  n=267 |
| **Respondent type** | | | | |
| Adults | 311 (40%) | 100 (40%) | 100 (40%) | 111 (42%) |
| Caregivers | 458 (60%) | 150 (60%) | 152 (60%) | 156 (58%) |
| **Gender** | | | | |
| Male | 267 (35%) | 63 (25%) | 113 (45%) | 91 (34%) |
| Female | 502 (65%) | 187 (75%) | 139 (55%) | 176 (66%) |
| **Setting** | | | | |
| Urban | 251 (33%) | 81 (32%) | 81 (32%) | 89 (33%) |
| Rural | 518 (67%) | 169 (68%) | 171 (68%) | 178 (67%) |
| **Highest level of education** | | | | |
| No formal education | 220 (29%) | 111 (44%) | 12 (5%) | 97 (36%) |
| Primary | 201 (26%) | 93 (37%) | 28 (11%) | 80 (30%) |
| Secondary | 181 (24%) | 27 (11%) | 101 (40%) | 53 (20%) |
| College / technical school | 82 (11%) | 14 (6%) | 60 (24%) | 8 (3%) |
| Graduate | 81 (11%) | 5 (2%) | 47 (19%) | 29 (11%) |
| **Mean age** | | | | |
|  | 32.6 (10.4) | 32.9 (12.7) | 33.2 (9.3) | 31.7 (9) |
| **Healthcare Professional s** | | | | |
|  | Total  n=532 | Uganda  n=120 | Nigeria  n=200 | Ivory Coast  n=212 |
| **Specialty** | | | | |
| Nurse | 190 (36%) | 50 (42%) | 50 (25%) | 90 (42%) |
| Community Health Worker | 196 (37%) | 50 (42%) | 50 (25%) | 96 (45%) |
| Pharmacist | 146 (27%) | 20 (16%) | 100 (50%) | 26 (12%) |
| **Gender** |  |  |  |  |
| Male | 348 (65%) | 56 (47%) | 117 (58%) | 175 (83%) |
| Female | 184 (35%) | 64 (53%) | 83 (42%) | 37 (17%) |
| **Setting** |  |  |  |  |
| Urban | 219 (41%) | 67 (56%) | 60 (30%) | 92 (43%) |
| Rural | 313 (59%) | 53 (44%) | 140 (70%) | 120 (57%) |
| **Mean age** | | | | |
|  | 7.9 (5.7) | 9.2 (6.8) | 7.6 (5) | 7.5 (5.5) |
