# Supplementary material for: Understanding barriers to effective malaria diagnosis and treatment: A mixed-methods study in Uganda, Nigeria, and Ivory Coast
Source: PLOS Glob Public Health. 2026 Aug 17;6(8):e0006921. doi: 10.1371/journal.pgph.0006921 (PMC13480639; doi:10.1371/journal.pgph.0006921)
Supplement: Supplementary file 7 [file pgph.0006921.s007.docx]

**S5 Table: Quantitative Sample Quotas**

| **Quantitative Sample Quotas** | | | |
| --- | --- | --- | --- |
| **Adults** | | | |
| **Age of respondent** | | | |
| Age (18 years +) | 100% | 100% | 100% |
| **Experience with malaria in the last 5 years** | | | |
| Between 1 and 50 | 100% | 100% | 100% |
| **Setting** | | | |
| Urban | 30% | 30% | 30% |
| Rural | 70% | 70% | 70% |
| **District** | | | |
| Acholi |  | 50% |  |
| East-Central Busoga |  | 50% |  |
| Bas-Sassandra |  |  | 50% |
| La Vallee Du Bandaman |  |  | 50% |
| Sokoto | 50% |  |  |
| Taraba | 50% |  |  |
| **Caregivers** | | | |
| **Age of respondent** | | | |
| Age (18 years +) | 100% | 100% | 100% |
| **Children in their care** | | | |
| At least 1 child in their chase between 0 months and 17 years | 100% | 100% | 100% |
| **Age of child in their care** | | | |
| 0-3 months | Minimum 20% | Minimum 20% | Minimum 20% |
| 4-6 months | Minimum 20% | Minimum 20% | Minimum 20% |
| 7-12 months | Minimum 13% | Minimum 13% | Minimum 13% |
| 1-4 years | Minimum 13% | Minimum 13% | Minimum 13% |
| 5-7 years | Minimum 10% | Minimum 10% | Minimum 10% |
| 8+ years | Minimum 10% | Minimum 10% | Minimum 10% |
| **Setting** | | | |
| Urban | 30% | 30% | 30% |
| Rural | 70% | 70% | 70% |
| **District** | | | |
| Acholi |  | 50% |  |
| East-Central Busoga |  | 50% |  |
| Bas-Sassandra |  |  | 50% |
| La Vallee Du Bandaman |  |  | 50% |
| Sokoto | 50% |  |  |
| Taraba | 50% |  |  |
| **Healthcare Professional** | | | |
| **Specialty** | | | |
| Nurse | 42% | 25% | 40% |
| Community Health Worker | 42% | 25% | 40% |
| Pharmacist | 16% | 50% | 10% |
| **Years in practice** | | | |
| Between 2 and 35 years | 100% | 100% | 100% |
| **Responsibility in malaria** | | | |
| One or more of the following responsibilities:   - Discussing malaria symptoms with patients - Diagnosing patients with malaria - Recommending/prescribing medication to patients with malaria - Follow up care for patients with malaria | 100% | 100% | 100% |
| **Patient types** | | | |
| 1% of malaria patient population to be infants weighing less than 5kg and 1% of malaria patient population to be infants weighing more than 5kg | 100% | 100% | 100% |
| **Setting** | | | |
| Urban | 30% | 30% | 30% |
| Rural | 70% | 70% | 70% |
| **District** | | | |
| Acholi |  | 50% |  |
| East-Central Busoga |  | 50% |  |
| Bas-Sassandra |  |  | 50% |
| La Vallee Du Bandaman |  |  | 50% |
| Sokoto | 50% |  |  |
| Taraba | 50% |  |  |
